# Cross-cultural adaptation of mental health screening instruments for Samoan adolescents

**DOI:** 10.1371/journal.pmen.0000106

**Published:** 2025-02-11

**Authors:** Emma J. Mew, Sarah R. Lowe, Ariel Galea’i, Francine Iopu, Jean Anderson, Joshua Naseri, Leiema Hunt, Tamari Mulitalo-Cheung, Okenaisa Fauolo-Manila, Si’itia Soliai-Lemusu, Nicola L. Hawley, Jueta McCutchan-Tofaeono

**Affiliations:** 1 Department of Chronic Disease Epidemiology, Yale School of Public Health, Yale University, New Haven, Connecticut, United States of America; 2 Department of Social and Behavioral Health Sciences, Yale School of Public Health, Yale University, New Haven, Connecticut, United States of America; 3 Lyndon B. Johnson (LBJ) Tropical Medical Center, Pago Pago, American Samoa, United States of America; 4 Department of Health, American Samoa Government, Pago Pago, American Samoa, United States of America; 5 Obesity, Lifestyle and Genetic Adaptations (OLAGA) Study Group, Pago Pago, American Samoa, United States of America; 6 Samoan Studies Institute, American Samoa Community College, Pago Pago, American Samoa, United States of America; 7 Empowering Pacific Island Communities (EPIC), Pago Pago, American Samoa, United States of America; Assiut University, EGYPT

## Abstract

Mental health problems appear common among Pacific Islander adolescents. The lack of culturally adapted mental health screening instruments is a barrier to establishing prevalence estimates needed to inform resource allocation and health system budgeting. Following the Gjersing et al. (2010) guidelines, we adapted five clinical mental health screening instruments to measure symptoms of depression, anxiety, post-traumatic stress, and suicide-related indicators for Samoan adolescents. In collaboration with clinical experts and American Samoan adolescents, we employed a four-stage incremental approach: (1) establishing expert team consensus to modify items for conceptual equivalence; (2) an iterative process of group-based forward- and back-translations; (3) adolescent piloting testing using an online survey; and (4) an adolescent focus group to finalize instruments and to develop administrative guidelines. We adapted the Patient Health Questionnaire-9 Modified for Teens (PHQ-9M), the Generalized Anxiety Disorder 7 (GAD-7), Child PTSD Symptom Scale Self-Report for the Diagnostic and Statistical Manual of Mental Disorders, Fifth Edition (CPSS-5), and the CPSS-5 Trauma Screener. We also developed a deliberate self-harm questionnaire based on the Avon Longitudinal Study of Parents and Children (ALSPAC) questionnaire and the Self-Injurious Thoughts and Behaviors Interview (SITBI). The adult expert committee modified the English-language wordings in each tool to improve local relevance and comprehension; adolescent pretesting led to two minor changes to the CPSS-5. Participants reported concerns about honesty in responding to highly sensitive questions; to address this, the focus group provided additional administrative guidelines. We provide the first cross-culturally adapted mental health screening instruments for use among Samoan adolescents. These instruments provide an opportunity to measure prevalence and inform public health policy through future population-based surveys. Further research should evaluate cross-cultural validity, measurement equivalence, and concordance with clinical screening to aid in clinical diagnostics for screening programs in Samoan healthcare settings.

## Introduction

Mental health problems are common among adolescents worldwide [1]. One review identified a 13.4% global pooled prevalence of mental health disorders among children and adolescents [2]. Within the United States (US), recent data describes a marked increase in the proportion of adolescents who experience poor mental health [3–7]. Between 2011 and 2021 the *Youth Risk Behavior Survey* (YRBS) reported an approximately 40% increase in the proportion of US adolescents who reported persistent feelings of sadness and hopelessness as well as marked increases in suicidal thoughts and behaviors [8]. While these increases were present across every racial and ethnic group, there remained disparities in prevalence by both ethnicity and geography, with persistently poorer outcomes among ethnic minority groups [8]. Ethnic minority groups that have received limited attention–both in attempts to describe prevalence of poor mental health and to intervene–are those that comprise Pacific Islanders. Suicide was the leading cause of death for US Native Hawaiians/Pacific Islanders between ages 15–24 in 2019 [9]. The 2021 YRBS documents lower prevalence of poor mental health among Native Hawaiian and Pacific Islander adolescents than among those who identified as White and that suicide attempts and related injuries were greater among this group relative to White adolescents [8].

Samoan adolescents, in particular, are thought to experience a high prevalence of depression, anxiety, substance use, and suicidal thoughts and behaviors. The 2017 iteration of the *Global School-Based Student Health Survey* found that among 13-17-year-old students in the independent state of Samoa, 22.3% seriously considered attempting suicide and 21.7% attempted suicide one or more times in the 12 months preceding the survey [10]. In the 2012 *Youth2000 Survey* in New Zealand, approximately 32% of female Samoan students and 22% of male Samoan students deliberately hurt themselves and approximately 12% of girls and 4% of boys had attempted suicide in the previous 12 months [11]. This survey also found that approximately 15% of female Samoan students and 9% of male Samoan students reported significant depressive symptoms [11]. In American Samoa, an unincorporated US territory with a population of roughly 50,000 people [12], there was a cluster of 11 adolescent suicides in late 2020 [13] and recent research suggests that suicidal thoughts and behaviors, depression, anxiety, and substance use appear common among adolescents [13,14]. The 2013 iteration of the *Youth Risk Behavior Survey* (the most recent attempt to quantify the burden of mental health concerns in American Samoa) found that 23% of American Samoan students in grades 9–12 experienced suicidal ideation in the previous 12 months and 19% had attempted suicide at least once in their lifetime [15].

Beyond the age of the existing data from American Samoa [15], which was collected nearly a decade ago and limits the ability to inform current health system resource allocation, there may be concerns about validity of existing data on the prevalence of mental health concerns. More recent efforts to quantify mental health prevalence–including the 2021 iteration of the YRBS (data not yet made available) and other non-profit-led surveys [14]–have relied upon non-diagnostic measures that have yet to be validated within a Samoan context. While several self-administered screening instruments exist for adolescents, and have been well-validated for use among non-Samoan populations, it is important to examine their validity in each new context. Mental health providers have used two commonly used measures, the Patient Health Questionnaire-9 (PHQ-9) and the Generalized Anxiety Disorder 7 (GAD-7) instruments, to assist in diagnoses in American Samoa, without formal validation and have raised concerns that they may need validation for broader use.

Concerns about the use of mental health tools without contextual validation arise from the knowledge that conceptions of mental health and illness are known to differ among Samoan compared to Western populations [16]; the concept of self is considered crucial to understand Samoan conceptions of mental health, but studies suggest that Samoan conceptions of self are intrinsically relational [17–19] and role-based [14] and could be misaligned with Western psychiatric theories of self [17]. Traditional Samoan conceptions of mental illness are also unique, sometimes viewing mental illness as a “*punishment from God or an indication of a spiritual rupture*” [14]. There is also concern that operational equivalence might not be held when applying English-language instruments to Samoan-speaking adolescents. The first step towards updated, valid prevalence estimates that can better assist with resource allocation in Samoan communities is the development of cross-culturally valid instruments to measure a variety of common mental health conditions and symptoms. To our knowledge, no adolescent mental health screening tools have been cross-culturally adapted or validated for a Samoan population. The objective of this study was to take a rigorous and community-partnered approach to cross-culturally adapt several mental health screening instruments for common mental health problems among Samoan adolescents and provide preliminary assessments of face validity. The goal was to develop instruments that could be generalizable to Samoan communities in the United States and across the Samoan diaspora.

## Materials and methods

### Ethics statement

Ethical approval was obtained from the Yale University (#2000028354) and the American Samoa Department of Health (#00001249) Institutional Review Boards. Written informed consent was waived for adult participants. Adolescent participants provided written assent and one legal guardian provided written consent.

### Study overview

**Fig 1:**
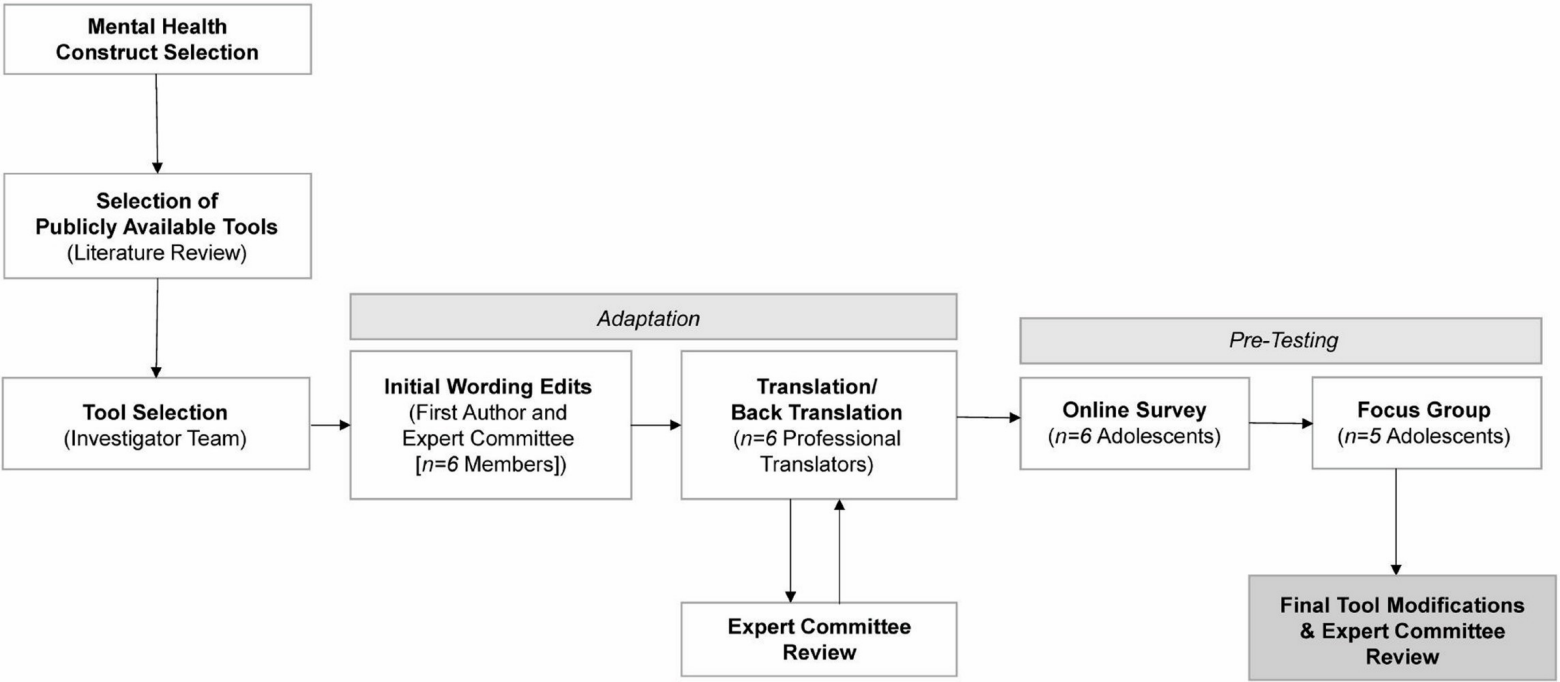
Overview of the adaptation process.

This cross-cultural adaptation process was part of a larger qualitative study in American Samoa [13,20]. We took a community-partnered approach, collaborating closely with local clinicians, adolescents, young adults, and community leaders during all stages. When possible, we followed the Gjersing et al. [21] guidelines for cross-cultural adaption but modified these principles to better align with Samoan practices for consensus-making [22,23] and for feasibility, when needed. Adult recruitment occurred between October 19 2020 to February 1 2021. Survey recruitment occurred between June 8 2022 to August 16 2022; focus group recruitment occurred between June 8 2022 to June 1 2023. Fig 1 provides an overview of the adaption process.

### Construct selection

Given our on-going research partnerships in American Samoa, instruments were selected based on local mental health needs, with the intention to inform an upcoming territorial school-based survey. To identify which constructs to measure, we analyzed results from a larger qualitative study that interviewed adult key informants (≥18 years) based in American Samoa between October 2020 and February 2021 (see Mew et al., 2023 for methodology [13]). Participants were asked open-ended questions on a broad range of topics (Table A in S1 Data), which included: “*What are the most common mental health problems among adolescents in American Samoa*, *if any at all*?” and “*What are the signs that an adolescent in American Samoa is struggling with their mental health that someone else might notice*?”. We used responses to these questions, as well as others that captured relevant information, in consultation with local clinical experts (including SLL and JM, among others), to arrive at the final list of mental health problems–or constructs–for which to select measurement instruments that would be most useful to inform local policy and practice.

### Instrument selection

After selecting the list of constructs, we then identified publicly available measurement instruments. Given our intent that these tools could be used in both surveys and clinical practice, where possible, we sought to identify tools that would meet the following criteria: (1) adolescent self-reported (rather than clinician administered); (2) currently implemented in screening and clinical settings in American Samoa; (3) validated for the Diagnostic and Statistical Manual of Mental Disorders, Fifth Edition (DSM-5); (4) sufficient validity among adolescent populations; and (5) administered in <15 minutes. To identify candidate instruments, EM systematically examined the literature and held discussions with collaborators to arrive at the list of candidate instruments for each construct. From there, NH, SRL, JM, and EM reviewed instrument candidates and selected the final instruments to adapt.

### Expert committee

We assembled an expert committee of six members to guide the cross-cultural adaptation process. This included members with scientific expertise (NH; professor of epidemiology with expertise in Samoan health research and adapting and translating research measures for use in Samoan communities), local clinical expertise (JM, JA, and AG; Samoan mental health clinicians serving adolescent populations across in- and out-patient settings), clinical expertise in trauma and stressor-related conditions (SRL; clinical psychologist and trauma researcher) and one Samoan young adult (FI) with professional experience delivering mental health content to American Samoan adolescents. Samoan members were bilingual in Samoan and English. At the time of this project, this committee included all doctoral-level clinical psychologists (JM and JA) practicing in American Samoa.

### Translation and adaptation process

The first stage of the Gjersing et al [21] process is to assess and modify instruments for conceptual equivalence (ensuring instrument domains and the emphasis placed on different domains is the same in both cultures), item equivalence (that the items are relevant and acceptable in both cultures), and semantic equivalence (the meaning of the items is the same in both cultures) [21,22,24]. Our team first modified the English wordings. Given the lack of data on Samoan conceptions of mental health problems, we leveraged secondary data from our qualitative study (as described above [13]) where adult participants explained their perspectives of the signs and symptoms of mental illness among Samoan adolescents, and to which we later broadly validated among adolescent focus groups (focus group methodology described in Mew et al., 2024 [20]). Using the qualitative data, EM modified English wordings to represent the described Samoan-specific conceptions and presentations of each mental health problem. Expert committee members (JM, FI, NH, and SRL) then made further modifications until consensus was reached.

We then forward- and back-translated the modified instruments. We deviated from the Gjersing guidelines, which recommend three translators (two to translate independently, and one to synthesize the forward translations) and three back-translators (two to back-translate independently, and one to synthesize the back-translations). Instead, we followed an approach that leveraged knowledge from a team of six translators/back-translators, which worked in the *Samoa-na-lua* style, which is a group consensus-based approach, similar to a method previously used in the independent state of Samoa [23]. The items were first forward translated by a team of five professional translators at the Samoan Studies Institute at the American Samoa Community College. This team used a locally-developed process to align with Samoan conceptions of “*soalaupule*”–meaning, “*consultation*”, or the traditional Samoan inclusive decision-making process where decisions are based on the consensus of all [25]. Specifically, four translation professionals translated each item individually, and then this group reviewed the translations as a team, with each English item and its Samoan translation displayed on screen. During this process, the team would reconcile and merge translations into one single translation. To address discrepancies, more research was done and presented to show why a particular translator believed their chosen terms should be used. When consensus was reached, the Director of the Institute (OFM) reviewed the final translations and either approved the translation or returned it to the translators for adjustments. If adjustments were minimal, OFM would make final adjustments herself. The translators were bilingual (fluent in both English and Samoan) and bicultural (familiar with both Samoan and American cultures). We then took this synthesized version of translations and conducted one back-translation, conducted by a bilingual and bicultural public health researcher (JN) with experience in adolescent mental health research.

The expert committee then took an iterative process to further modify the instruments according to cultural relevance, acceptability, and conceptual equivalence. We circulated the instruments with the original wordings, our modifications to the English wordings, the professional translations, and the back-translation for expert committee review. From here, we held three online Zoom meetings between April to June 2022 to discuss areas flagged by team members and review each instrument item in English and Samoan. During this process, we continued to modify the English and Samoan wording of the items (drawing on the final forward- and back-translations). We then returned survey items back to the translation team as needed for further adjustments until consensus was reached.

### Pretesting with adolescents through an online survey

We then pretested the tools with adolescents to assess and adapt the instruments for acceptability and operational equivalence (i.e., that the questionnaires can be used in the same way by its target population in both cultures) [22]. We did this with an online survey using Research Electronic Data Capture (REDCap) software [26] that presented the English and Samoan wordings of each instrument item side-by-side. Before implementation, we informally piloted this survey with two adults. Though the Gjersing guidelines recommend pretesting the instruments with 30 to 40 respondents, that was not feasible given resource restraints, so we instead developed an alternative approach that leveraged youth feedback through an online survey followed by a focus group. We administered the survey to six bilingual Samoan adolescents in September 2022 (n = 3 girls and n = 3 boys; average age of 16.4 years and an average of 14.2 years spent living in American Samoa; all fluent in Samoan). Participants were recruited from our broader qualitative project; specifically, they were handpicked from a pool of participants who were most engaged during a previous mental health focus group, as described elsewhere [20]. Due to technical difficulties, one participant was unable to finish all questions, which left n = 5 adolescents completing questions for most items.

The survey included each item from all included instruments. The objective of the survey was to gain feedback from adolescents as to whether they understood the wording and whether they would answer the questions honestly. For each prompt, we then asked participants to answer two questions on a Likert scale about comprehension and honesty, specifically: “*Is this question easy to understand*?” (with the options: “Strongly disagree (total nonsense)”, “Disagree”, “Neutral”, “Agree”, “Strongly agree (totally understandable)”) and “*Would adolescents answer this question honestly*?” (with the options: “Strongly disagree (totally dishonest)”, “Disagree”, “Neutral”, “Agree”, “Strongly agree (totally honest/truthful)”). When adolescents answered “Neutral”, “Disagree” or “Strongly Disagree”, they were then prompted with a qualitative text box asking for further information (specifically, either “*Why is this question hard to understand*?” or “*Why might adolescents not be honest in answering this question*?”). We then analyzed items that received at least one “disagree” or “strongly disagree” response in the surveys. These items were first reviewed by EM and NH, and additional members of the research team when needed, to identify whether further discussion was needed to prioritize items for focus group discussion (necessary given the time restraints in only having one session). Results of this pre-testing phase are presented in each of the Tables below, although no formal analysis was undertaken.

### Focus group with adolescents

After collecting survey data, we recruited adolescent participants to participate in a focus group, whose aim was to modify the instruments to improve honesty and understandability, confirm understandability of certain item concepts, and discuss specific safety concerns. We conducted a two-hour focus group in June 2023 with bilingual Samoan youth (13–19 years) who lived in American Samoa. Our sampling frame maximized diversity across gender, as the survey results led us to believe that experiences would likely differ between boys and girls. Five adolescents, recruited through our team’s networks, attended the focus group (n = 3 girls, n = 2 boys; average age of 17.3 years and an average 16.3 years living in American Samoa); one of these adolescents participated in both the survey and focus group, providing us with a sample of n = 10 adolescents who provided pretesting feedback in this study. Participants were sent the list of tools to review prior to the focus group. All participants identified as being of Samoan ethnicity; some also identified that they were multi-ethnic. Other demographic characteristics are not described to protect confidentiality (necessary given the small and close-knit community).

The focus group was led by two experienced Samoan moderators (LH and JN) with public health training (interviewer positionalities in Table B in S1 Data). EM (project principal investigator) observed the session as it encouraged participants to explain cultural nuances in explicit terms that could be understood by an outsider and also contributed to the discussion as needed. Interview prompts were read primarily in English, but the general discussion used both English and Samoan languages. The interview guide (Table C in S1 Data) built on questions generated from instances where the sample communicated strong concerns about understandability and/or honesty for a particular item. As part of this focus group, we also asked high-level questions about promoting an environment for honesty and safety. Intentional efforts were taken to breakdown hierarchies and build trust and rapport between the adults and adolescents in the focus group, such as playing ice breaker games and reaffirming the importance of each participant’s perspectives and contributions to the work. Sections of the transcript in Samoan were translated into English by a Samoan colleague. EM was responsible for reading and reviewing the focus group transcript and the excerpts from the adult semi-structured interviews to generate themes.

### Finalizing tools

Incorporating feedback from the adolescents, EM modified the instruments and sent them back to the expert committee for final signoff. TMC and OFM, experts on Samoan language and translation, also reviewed the final tools (presented in a downloadable format in S1–S4 Texts) before dissemination. We also sent the final tools to a Samoan research expert (KS) who has lived in American Samoa, the independent state of Samoa, and New Zealand, who confirmed that the instruments should be valid for use among Samoan adolescents across the Samoan diaspora.

## Results

### Construct selection

Adult participants from the qualitative semi-structured interviews identified that suicidal thoughts and behaviors (including ‘cutting’ as well as suicidal attempts), depressive disorders, trauma- and stressor-related disorders, and anxiety disorders appear to be the most common mental health conditions among adolescents in American Samoa; those involved in providing clinical services also confirmed that depression, anxiety, and post-traumatic stress disorders are most commonly observed among adolescents in their practice. Traumatic events were described as being very common, particularly sexual assault and physical discipline. Participants described other mental health conditions, but noted that they were less common: bipolar disorder, neurodevelopmental disorders (e.g. attention-deficit/hyperactivity disorder), schizophrenia, and feeding and eating disorders (with an emphasis on over-eating). Based on these results, the research team determined that the most important mental health problems to quantify among adolescents in American Samoa would be: depression, anxiety, post-traumatic stress disorder (which would include potentially traumatic experiences), and suicidality. Substance use (alcohol, methamphetamines, marijuana, and nicotine) was also reported as common, usually as a coping mechanism. We did not adapt an instrument to measure substance use, as several single-item questions involving alcohol, methamphetamine, tobacco, and marijuana use have been regularly employed in internationally deployed school-based surveys in American Samoa and the independent state of Samoa [10,15] and were developed for cross-cultural applications. The authorship team also felt that these existing measures were likely valid without additional cross-cultural adaptation, and thus felt that our resources would be better suited to adapting other instruments.

### Instrument selection

To best capture the four identified constructs, we selected the *Patient Health Questionnaire-9*: *Modified for Teens* (PHQ-9M) [27] to measure depressive symptoms and provide a clinical measurement of suicide risk; the *Generalized Anxiety Disorder-7* (GAD-7) [28] to measure anxiety symptoms; and the *Child PTSD Symptom Scale for DSM-5* (CPSS-5) [29] along with its corresponding *Trauma Screen* to measure exposure to potentially traumatic events and post-traumatic stress symptoms. Of these instruments, the *PHQ-9* (original version for adults) and *GAD-7* were reported to be used in clinical practice among adolescents in American Samoa. As a supplement to the suicide risk screener in the *PHQ-9M*, and existing measures employed in the YRBS, we developed a new questionnaire to supplement knowledge gaps with respect to self-harming behavior and intentionality, suicide-related help-seeking behaviors, social networks, and more detailed questions on individual history of suicidal thoughts and attempts. Though we identified several eligible suicide screening instruments, most were developed to be clinician administered, and those that were self-administered were developed for use among adults, making them not well suited for this context. To develop the new questionnaire, we drew questions from the Avon Longitudinal Study of Parents and Children (ALSPAC) self-harm questionnaire [30], given that it was developed to assess a variety of suicidality questions for 16 year olds in a self-administered survey format. As a supplement, we drew questions from the Self-Injurious Thoughts and Behaviors Interview (SITBI) [31] for questions related to suicide attempts and suicidal thoughts. See Table D in S1 Data for our list of candidate instruments and rationale for the selection of each instrument for each construct.

### Instrument adaptation

The majority of modifications to the English wordings came from the expert committee; the adolescent focus group only generated three minor modifications to the CPSS-5 and the CPSS-5 Trauma Screener. Before starting, given that adolescents in American Samoa speak both English and Samoan, and that our expert committee agreed that reading comprehension is limited for youth in both languages, the committee recommended that it would be important to present all instrument items in both English and Samoan, side-by-side. The committee thought this would help with understandability so that adolescents could piece together understanding based on both translations as needed. This would also then allow for increased generalizability to other Samoan populations (in both the independent state of Samoa–which is predominantly Samoan speaking–and to Samoans living in English-speaking countries, such as in the continental United States and New Zealand).

The pretesting survey included 86 survey prompts. Given that the results from the survey indicated repeated themes related to honesty that transcended one specific item, much of the adolescent focus group centered around development of administrative practices that would help improve honesty across all items. We present representative quotations from the survey and focus group throughout, but we do not present participant identifiers because the sample was so small. See Tables E, F, G, H, and I in S1 Data for a full presentation of results from the adolescent surveys and focus group decisions for each instrument.

One common recommendation from the expert committee that transcended all instruments was to present the Samoan translations using very simple language, given concerns regarding Samoan literacy. This was further validated during the focus group, as there were common concerns in understanding the Samoan translations and challenges in finding agreement for the Samoan translations. Despite this, members of the expert committee concurred that this issue is mainly due to the complexity of the Samoan language and that it would be challenging to achieve consensus in Samoan translations that everyone could follow, given the many ways to translate English text into Samoan based on the level of formality, among other factors. Given that this was the primary issue, for these instances, the focus group concluded that the tools were sufficiently understandable as long as each instrument would be presented with the English and Samoan versions side-by-side, especially since most adolescents would likely rely primarily on the English wordings. These suggestions are further supported by recent locally-conducted surveys in American Samoa, such as the 2000 Language Status survey that showed that Samoan is only used to converse 67% of the time at home and 38% at school [32], demonstrating the need for both English and Samoan translations.

### PHQ-9M

Table 1 provides the cross-culturally adapted PHQ-9M for Samoan adolescents along with changes made from the original instrument. The adult expert committee modified the English wording for six of the thirteen PHQ-9M questions (46%; items 1, 2, 6, 8, 9, and 11 in Table 1). No changes were made to the instrument prompt. The majority of changes were to simplify language and improve comprehension. One exception to this was for item 2, as the expert team expanded the item to also include little interest in “*talking to people*” as the adult stakeholder interviews suggested a major symptom of depression among youth in American Samoa is social withdrawal–a noticeable deviation from Samoan social norms–and this was not included as a concept in the original instrument. The expert committee felt this change was essential to capture symptom presentation in a Samoan context (Table E in S1 Data). The expert committee also modified items 2 and 11 to include more locally relevant examples (Table E in S1 Data). The expert committee considered adding the concept of anger or aggression, as to build on the definitions of depression from the qualitative interviews, as adult participants described that Samoan adolescents often channel their depression into fights/aggression (as one participant stated: “*it fits into the culture better*”). The committee, however, did not make this change as to be most conservative in protecting psychometric validity.

**Table 1:** Cross-culturally adapted Patient Health Questionnaire-9: Modified for Teens (PHQ-9M) for Samoan adolescents with changes from the original PHQ-9M and pretesting results for understandability and honesty among n = 6 adolescents.

	Changes from the original PHQ-9M	PHQ-9M adapted for Samoan adolescents		Pretesting	
				% Comprehension* Is this question easy to understand?	% Honesty* Would adolescents answer honestly?
0	No changes	How often have you been bothered by each of the following symptoms during the past two weeks? For each symptom put an “X” in the box beneath the answer that best describes how you have been feeling: Not at all Several days More than half the days Nearly every day	I le lua vaiaso talu ai, e fa’afia ona e a’afia i auga ta’itasi nei? Tusi se “X” i le pusa i lalo ane o le tali pito talafeagai e faamatala ai lou lagona: E leai ni auga Ni nai aso Sili atu ma le afa o aso Toeitiiti lava o aso uma	Agree: 83% (n = 5) Neutral: 17% (n = 1)	Agree: 83% (n = 5) Disagree: 17% (n = 1)

1	Feeling sad, irritable (for example, easily annoyed), or hopeless?	Feeling sad, irritable (for example, easily annoyed), or hopeless?	Lagona le faanoanoa, maitaita, (mo se faataitaiga, ita gofie) po ua leai se faamoemoe?		
2	Not really interested in doing things or talking to people (such as not wanting to spend time with friends or participate in family or church activities)?	Not really interested in doing things or talking to people (such as not wanting to spend time with friends or participate in family or church activities)?	E faalefiafia tele i mea e fai poo le talanoa foi i tagata (e pei o le lē fia mafuta ma uo pe auai i mea e fai a le aiga poo le ekalesia fo’i)?	Agree: 83% (n = 5) Neutral: 17% (n = 1)	Agree: 67% (n = 4) Neutral: 17% (n = 1) Disagree: 17% (n = 1)
3	No changes	Trouble falling asleep, staying asleep, or sleeping too much?	Faigata ona moe, faigata ona faaauau le moe, pe moe so’o?	Agree: 100% (n = 6)	Agree: 100% (n = 6)
4	No changes	Poor appetite, weight loss, or overeating?	Tau le manogi se ai, alu le tino (lusi); po’o le soona‘ai?	Agree: 67% (n = 4) Disagree: 33% (n = 2)	Agree: 100% (n = 6)
5	No changes	Feeling tired, or having little energy?	Lagona le lē lava, faapalupē/le lava le malosi?	Agree: 100% (n = 6)	Agree: 83% (n = 5) Neutral: 17% (n = 1)
6	Feeling down about yourself—or feeling that you are a failure, or that you have disappointed yourself or your family?	Feeling down about yourself–or feeling that you are a failure, or that you have disappointed yourself or your family?	Faanoanoa ona o oe lava ia–poo lagona o oe o se tagata toi’lalo, pe ua e faalumaina oe ma lou aiga?	Agree: 100% (n = 6)	Agree: 83% (n = 5) Disagree: 17% (n = 1)
7	No changes	Trouble concentrating on things like schoolwork, reading or watching TV?	Faafaigata ona tuu atoa lou mafaufau i au meaaoga, faitaugatusi poo le matamata o le TV?	Agree: 83% (n = 5) Neutral: 17% (n = 1)	Agree: 83% (n = 5) Disagree: 17% (n = 1)
8	Moving or speaking so slowly that other people could have noticed?	Moving or speaking so slowly that other people could have noticed? Or the opposite–being so fidgety (for example, can’t sit still) or restless that you were moving around a lot more than usual?	Gaoioi poo le tautala lemu lava e ono amata ai ona iloa mai e isi? Poo le lē mafai ona nofo filemu pe gaoiā ma ua fealuai solo e sili atu nai lo le mea e masani ai?	Agree: 83% (n = 5) Disagree: 17% (n = 1)	Agree: 83% (n = 5) Disagree: 17% (n = 1)
	Or the opposite–being so fidgety (for example, can’t sit still) or restless that you were moving around a lot more than usual?				
9	Thoughts or feelings that you would be better off dead, or of hurting yourself in some way?	Thoughts or feelings that you would be better off dead or hurting yourself in some way?	Mafaufauga poo faalogona e sili ai le oti poo le faia o nisi tulaga e faao’o ai le tiga o le tino ia te oe?	Agree: 100% (n = 6)	Agree: 83% (n = 5) Neutral: 17% (n = 1)
10	No changes	In the ***past year*** have you felt depressed or sad most days, even if you felt okay sometimes? Yes/No	I le ***tausaga ua mavae***, na e lagona ai le faanoanoa loloto poo le faanoanoa i le tele o aso, tusa pe na iai nisi taimi na lelei ai? Ioe/Leai	Agree: 100% (n = 6)	Agree: 100% (n = 6)

11	If you are experiencing any of the problems discussed/listed above, how difficult have these problems made it for you to do your schoolwork, take care of chores at home or get along with other people? Not difficult at all Somewhat difficult Very difficult Extremely difficult	If you are experiencing any of the problems discussed/listed above, how **difficult** have these problems made it for you to do your schoolwork, take care of chores at home or get along with other people? Not difficult at all Somewhat difficult Very difficult Extremely difficult	Afai o e lagonaina se faafitauli o fa’atalanoaina i lenei pepa, o le a se **faigata** na oo i ai i le faiga o au meaaoga i le fale, faatinoga o feau i le fale, poo le galulue faatasi ma isi? Leai se faigata Faigata laitiiti Faigata tele Matuā faigata	Agree: 100% (n = 6)	Agree: 100% (n = 6)
12	No changes	Has there been a time in the past month when you have had serious thoughts about ending your life? Yes/No	Na iai se taimi i le masina ua tuanai na e manatu toto’a ai e te pule i lou ola? Ioe/Leai	Agree: 100% (n = 6)	Agree: 67% (n = 4) Neutral: 17% (n = 1) Disagree: 17% (n = 1)
13	No changes	Have you ***ever***, in your ***whole life***, tried to kill yourself or made a suicide attempt? Yes/No	Na iai se taimi i lou **olaga atoa**, na e taumafai ai e pule i lou ola? Ioe/Leai	Agree: 100% (n = 6)	Agree: 67% (n = 4) Neutral: 17% (n = 1) Disagree: 17% (n = 1)

*We merged responses of “strongly agree” and “agree” together and responses of “strongly disagree” and “disagree” together (full results in Table E in S1 Data).

Pretesting demonstrated that this instrument would be sufficiently understandable and elicit sufficiently honest answers (Table 1). For 11 of the 13 items (85%) and for the question prompt, no adolescent raised concerns in understandability (reporting either “disagree” or “strongly disagree” to “*Is this question easy to understand*?*”*). There was unanimous agreement (reporting either ‘agree’ or ‘strongly agree’) that eight of the 13 items (62%) were understandable and there were only two items (15%) where at least one adolescent disagreed (reported ‘disagree’ or ‘strongly disagree’) (items 4 and 8; Table 1). The focus group discussed why these two items were unclear and offered suggested wording changes to improve understandability (this included suggestions to change the wording of item 4 to include “*eating disorders*”). Ultimately, the group reached consensus to leave these items unchanged as the majority of the adolescents in the group preferred the original wording (Table E in S1 Data).

For seven of the 13 items (54%), at least one adolescent reported that they disagreed (reporting answers of “disagree” or “strongly disagree”) with the question “*Would adolescents answer this question honestly*?” (items 1, 2, 6, 7, 8, 12, and 13). Respondents provided several qualitative reasonings (see Table J in S1 Data); examples included: “*they don’t want the truth to come out*”, *“they’re going to lie so that they are positive about themselves but in reality they’re not*”, “*they feel as they can’t quite express their feelings to anyone or even a stranger*”, and “*they don’t want to be seen as weak or vulnerable*” (Table J in S1 Data). Given a general theme across items, the focus group discussed general approaches to improving honesty rather than changing the wording for any specific item (see Development of Administration Instructions section).

### GAD-7

Table 2 provides the cross-culturally adapted GAD-7 for Samoan adolescents along with changes made from the original instrument. The adult expert committee modified the English wording for five of the eight questions (63%; items 1, 3, 6, 7, and 8 in Table 2). No changes were made to the instrument prompt. All changes were to simplify the language for understandability or to add in contextually- and locally-relevant examples (see Table F in S1 Data for detailed reasoning). No changes were made to the instructions or Likert Scale answers.

**Table 2:** Cross-culturally adapted Generalized Anxiety Disorder-7 (GAD-7) for Samoan adolescents with changes from the original GAD-7 and pretesting results for understandability and honesty among n = 5 adolescents.

	Changes from the original GAD-7	GAD-7 adapted for Samoan adolescents		Pretesting	
				% Comprehension* Is this question easy to understand?	% Honesty* Would adolescents answer honestly?
0	No changes	Over the last two weeks, how often have you been bothered by the following problems?	I le lua vaiaso talu ai, e fa’afia ona faapopoleina oe i fa’afitauli o loo sosoo atu?	Agree: 100% (n = 5)	Agree: 100% (n = 5)
		Not at all Several days More than half the days Nearly every day	Leai ma se taimi Mo ni nai aso Sili atu i le afa o aso atoa Toeitiiti aso uma		
1	Feeling nervous, anxious, stressed or on edge	Feeling nervous, anxious, stressed or on edge	Lagona lē to’a, popole; mamafa se mea i le mafaufau/atuatuvale		
2	No changes	Not being able to stop or control worrying	Lē mafai ona taofi pe faatonutonu le lagona popole	Agree: 100% (n = 5)	Agree: 100% (n = 5)
3	Worrying too much about different things (such as your future, disappointing your family/how other people might judge you, your responsibilities at home, school, and/or church, etc.)	Worrying too much about different things (such as your future, disappointing your family/how other people might judge you, your responsibilities at home, school, and/or church, etc.)	Soona popole i mea eseese (faapei o lou lumanai, faanoanoa i le faamasino tagata o lou aiga poo isi tagata i au matafaioi i lou aiga, aoga, ma/poo le ekalesia, ma isi.)	Agree: 100% (n = 5)	Agree: 100% (n = 5)
4	No changes	Trouble relaxing	Faafaigata ona faato’afilemu	Agree: 100% (n = 5)	Agree: 100% (n = 5)
5	No changes	Being so restless that it is hard to sit still	Ua matua lē to’afimalie ma ua i’u ina faigata ai ona mau pea le nofo	Agree: 100% (n = 5)	Agree: 80% (n = 4) Neutral: 20% (n = 1)
6	Becoming easily annoyed, irritable, or upset	Becoming easily annoyed, irritable, or upset	Ua maitaita, itagofie poo le lē fiafia	Agree: 100% (n = 5)	Agree: 100% (n = 5)
7	Feeling afraid as if something terrible might happen (for instance, to yourself, your family, or others)	Feeling afraid as if something terrible might happen (for instance, to yourself, your family, or others)	Lagona le fefe i se mea matautia e ono tula’i mai (fa’ataitaiga, ia te oe, lou aiga, po’o nisi tagata)	Agree: 100% (n = 5)	Agree: 100% (n = 5)
8	If you checked any problems discussed/listed above, how difficult have they made it for you to do your schoolwork, take care of chores at home, or get along with other people? Not difficult at all Somewhat difficult Very difficult Extremely difficult	If you checked any of the problems discussed/listed above, how difficult have they made it for you to do your schoolwork, take care of chores at home, or get along with other people? Not difficult at all Somewhat difficult Very difficult Extremely difficult	Afai o e lagonaina se faafitauli o fa’atalanoaina i lenei pepa, o le a se faigata na oo i ai i le faiga o au meaaoga i le fale, faatinoga o feau i le fale, poo le galulue faatasi ma isi? Leai se faigata Faigata laitiiti Faigata tele Matuā faigata	Agree: 80% (n = 4) Neutral: 20% (n = 1)	Agree: 80% (n = 4) Neutral: 20% (n = 1)

*We merged responses of “strongly agree” and “agree” together and responses of “strongly disagree” and “disagree” together (full results in Table F in S1 Data).

Pretesting with adolescents demonstrated that this instrument would be sufficiently understandable and would elicit sufficiently honest answers (Table 2). No participants raised concerns about understandability; seven of the nine items (78%) had all participants either “agree” or “strongly agree” that the item was understandable. Based on this, the adolescents made no further changes to either the English items or Samoan translations. For honesty, no participant reported that they “disagreed” or “strongly disagreed” with the question “*Would adolescents answer this question honestly*?”. Respondents, did however, report three qualitative reasons for items 5, 6, and 7 (see Table F in S1 Data and Table J in S1 Data). Examples include: “*they don’t want to be mean and selfish*” and “*lack to confidence to speak up*”. Similar to the PHQ-9M, we used the focus group to decide on general strategies to improve honesty, rather editing individual items. Table 3 provides the cross-culturally adapted Trauma Screen for Samoan adolescents along with the changes made from the original instrument. The adult expert committee modified the English wording for ten of the 20 question items (50%; items 1, 2, 4, 5, 6, 7, 8, 13, 15, and 16 in Table 3) and to both question prompts. Changes to the English wording of items were to accommodate the local context where some traumatic events were considered so normative that they might not be considered traumatic; for example, the committee changed item 2 to be *“seriously injured*” by a dog, since minor dog bites are common locally and not considered traumatic enough to be classified as a potentially traumatic event. Similarly, the committee changed the language to “*serious injury*” from sports to again not confuse it with minor injuries in this context. Likewise, for items 4–7, we replaced “*slapped”* with “*severely physically hurt*” to differentiate between the light slapping that remains a very common form of communicating irritation or frustration in this context [20] from intentional discipline/punishment. Given that this could potentially change the construct, we validated understanding of what “*severely physically hurt*” would mean for adolescents in the focus group, and this was interpreted as bruises on the body and “*whatever wound or the injury you have actually gets you to the hospital*. *Like you need to be treated right away*”. The remaining changes made to the English wording of items were to either simplify language for understandability or to add in contextually- and locally-relevant examples (see Table G in S1 Data for reasoning).

**Table 3:** Cross-culturally adapted Trauma Screener (as part of the CPSS-5) for Samoan adolescents with changes from the original Trauma Screener and pretesting results for understandability and honesty among n = 5 adolescents.

	Changes from the original Trauma Screen	Trauma Screen adapted for Samoan adolescents		Pretesting*	
				% Comprehension* Is this question easy to understand?	% Honesty* Would adolescents answer honestly?
0.1	Many children and adolescents go through frightening or stressful events. Below is a listed of frightening or stressful events that can happen. Mark YES if you have experienced any of these events. Mark NO if you have not experienced these events. Yes/No	Many children and adolescents go through frightening or stressful events. Below is a list of frightening or stressful events that can happen. Mark YES if you have experienced any of these events. Mark NO if you have not experienced these events. Yes/No	O le toatele o tamaiti ma talavou (laiti), e a’afia i ni tulaga e mafua ai ona fefefe ma atuatuvale e o’o i ni tulaga e fefefe ma atuatuvale ai. O loo i lalo atu le lisi o ni mea e tutupu e ono fefefe ma atuatuvale ai. Faailoga le IOE pe afai na e o’o i nisi o nei faafitauli. Faailoga le LEAI pe afai e te le’i o’o i ai. Ioe/Leai	Agree: 80% (n = 4) Neutral: 20% (n = 1)	Agree: 80% (n = 4) Neutral: 20% (n = 1)
1	A severe natural disaster such as a tsunami hurricane, or fire	A severe natural disaster such as a tsunami, hurricane, or fire	O se fa’alavelave fa’alenatura mata’utia e pei o galulolo/sunami, afā, po’o se mū		
2	Serious accident or injury caused by a car or bike crash, seriously injured by a dog, or serious injury from sports	Serious accident or injury caused by a car or bike crash, being seriously injured by a dog, or serious injury from sports	Fa’alavelave mata’utia po’o se manu’a e mafua mai i le lavea i se ta’avale po o se uila vili vae, pe se manu’a tigaina ona o se maile, pe mafua fo’i i ni ta’aloga	Agree: 80% (n = 4) Neutral: 20% (n = 1)	Agree: 80% (n = 4) Neutral: 20% (n = 1)
3	No changes	Being robbed by threat, force, or weapon	Na faoa ni au mea totino i le tau faamata’u, ave fa’amalosi pe i se auupega malosi	Agree: 80% (n = 4) Disagree: 20% (n = 1)	Agree: 80% (n = 4) Disagree: 20% (n = 1)
4	Being severely physically hurt or punished, or beaten by a relative	Being severely physically hurt or punished, or beaten by a relative	Na matuā afaina, fa’asala, pe na fasi fo’i e se isi o le aiga	Agree: 100% (n = 5)	Agree: 100% (n = 5)
5	Being severely physically hurt knifed, or beaten by a stranger	Being severely physically hurt, knifed, or beaten by a stranger	Na matuā afaina, po, afaina i se naifi/polo, pe fasi e se tagata ese	Agree: 80% (n = 4) Disagree: 20% (n = 1)	Agree: 80% (n = 4) Neutral: 20% (n = 1)
6	Seeing a relative get severely physically hurt, punished, or beaten	Seeing a relative get severely physically hurt, punished, or beaten	Vaai i se isi o lona auaiga o matuā afaina, fa’asala pe fasi	Agree: 100% (n = 5)	Agree: 100% (n = 5)
7	Seeing somebody in your community being severely physically hurt, severely physically punished, or beaten	Seeing somebody in your community being severely hurt, severely physically punished, or beaten	Vaai i se isi o lona nuu o matuā afaina, fa’asala pe fasi	Agree: 100% (n = 5)	Agree: 100% (n = 5)
8	Being touched in your sexual/private parts in a way you didn’t like or that made you feel uncomfortable by an adult/someone older who should not be touching you there	Being touched in your sexual/private parts in a way you didn’t like or that made you feel uncomfortable by an adult/someone older who should not be touching you there	Tagofia e se isi po’o se tagata matua ou itūtino sa e le tatau ona ia tagofia	Agree: 80% (n = 4) Neutral: 20% (n = 1)	Agree: 40% (n = 2) Neutral: 20% (n = 1) Disagree: 40% (n = 2)
9	No changes	Being forced/pressured to have sex at a time when you could not say no	Fa’amalosia e faia faigā aiga fa’amalosi i le taimi ua le mafai ona e fai atu i ai e leai	Agree: 80% (n = 4) Neutral: 20% (n = 1)	Agree: 60% (n = 3) Neutral: 40% (n = 2)
10	No changes	A family member or somebody close dying suddenly or in a violent way	Se isi o le auaiga ua oti fa’afuase’i pe i se auala saua	Agree: 100% (n = 5)	Agree: 100% (n = 5)
11	No changes	Being attacked, shot, stabbed, or seriously injured	Osofai’a, fana, tui po’o le manu’a tigāina	Agree: 80% (n = 4) Disagree: 20% (n = 1)	Agree: 100% (n = 5)
12	No changes	Seeing someone be attacked, shot, stabbed, or seriously injured or killed	Vaai i se isi o osofai’a, fana, tui pe manu’a tigaina pe ua fasiotia	Agree: 100% (n = 5)	Agree: 100% (n = 5)
13	Having a stressful or frightening medical procedure (e.g., heart or brain surgery)	Having a stressful or frightening medical procedure (e.g., heart or brain surgery)	Fefe po’o le atuatuvale i fa’agasologa o talavai a le falema’i (fa’ataitaiga taotoga o le fatu poo le fai’ai)	Agree: 100% (n = 5)	Agree: 100% (n = 5)
14	Being around a war, meaning a state of armed conflict that can result in death (which is not the same as inter-village conflicts)	Being around a war, meaning a state of armed conflict that can result in death (which is not the same as inter-village conflicts)	Sa e auai i se taua e pei o tulaga o feteenaiga faaauupegaina e mafai ona i’u ai i le oti (e ese mai i feeseeseaiga i totonu o nuu)	Agree: 80% (n = 4) Disagree: 20% (n = 1)**	Agree: 80% (n = 4) Neutral: 20% (n = 1)
15	Any other stressful or frightening event that has not been included above Describe:	Any other stressful or frightening event that has not been included above Describe:	So’o se isi mea na tupu e ono mafua ai le atuatuvale ma le fefe e le’o aofia i luga Fa’amatala:	Agree: 100% (n = 5)	Agree: 100% (n = 5)
16	Which of these events listed above bothers you most?	Which of these events listed above bothers you most?	le fea o vaega nei o lisi atu i luga e pito sili ona fa’alavelave ia te oe?	Agree: 80% (n = 4) Neutral: 20% (n = 1)	Agree: 80% (n = 4) Neutral: 20% (n = 1)
0.2	No changes	If you answered NO to all of the above questions, STOP. If you answered YES to any of the above questions, please answer the following questions.	Afai na e tali LEAI i fesili uma o lo’o i luga, TAOFI. Afai na e tali IOE i so’o se fesili lava o lo’o i luga, fa’amolemole tali fesili o lo’o mulimuli mai.	Agree: 100% (n = 5)	Agree: 100% (n = 5)
0.3	No changes	When the event happened, did you feel: Yes/No	I le taimi na tupu ai le fa’alavelave, na e lagonaina: Ioe/Leai		
17	No changes	Fear that you were going to die or be seriously injured?	Fefe ona o le a e oti pe manu’a tigaina?		
18	No changes	Fear that someone else was seriously hurt?	Fefe e iai se isi na manu’a tigaina?	Agree: 100% (n = 5)	Agree: 100% (n = 5)
19	No changes	Unable to help yourself?	Le mafai ona e fesoasoani ia te oe lava?	Agree: 100% (n = 5)	Agree: 100% (n = 5)
20	No changes	Shame or disgust?	Māasiasi po’o le inoino?	Agree: 100% (n = 5)	Agree: 100% (n = 5)

*We merged responses of “strongly agree” and “agree” together and responses of “strongly disagree” and “disagree” together (full results in Table G in S1 Data). **Changes were made after pretesting.

Pretesting did, however, identify several items that might be challenging for adolescents to understand (Table 3). No participants raised concerns about understandability for the three instrument prompts. Though 17 of the items (out of 20; 85%) raised no concerns about understandability, there were four items (17%) that received at least one response of “disagree” or “strongly disagree” to whether the item is sufficiently understandable (items 3, 5, 11, and 14), though no qualitative explanations were provided. Based on this, we discussed items 3, 11, and 14 in the focus group; we also discussed item 5 to validate interpretation (see last paragraph). Themes that arose were that the Samoan was too advanced to understand, but the group agreed that this would be fine if presented alongside the English translation. Adolescents also stated for item 14, that the concept of “*war*” was unclear, and questioned whether it would also apply to inter-village conflicts and disputes. Based on these discussions, in collaboration with our expert team, we modified the English and Samoan wordings to define war (meaning, armed conflict that could result in death, as this is something that would be potentially traumatic in a way to elicit post-traumatic stress symptoms).

For honesty, there were two items (10%) that received at least one response of “disagree” or “strongly disagree” to whether the item is sufficiently understandable (items 3 and 8). Five qualitative explanations were provided (Table J in S1 Data) and these responses centered around fear of exposing secrets, most notably sexual abuse, such as: “*still trying to gain the courage to speak and share their [sic] story*” and “*I think too many people would feel embarrassed or insecure if they talk about how they were sexually harassed*”. One adolescent expressed fear of repercussions from exposing domestic violence at home, such as: “*still the chances of them getting something worse or bad back*”. Based on this, similar to the PHQ-9M and GAD-7, we made effort in the focus group to discuss how to create a safe environment for adolescents to feel comfortable sharing stigmatised and sensitive family topics.

### CPSS-5

Table 4 provides the cross-culturally adapted CPSS-5 for Samoan adolescents along with the changes made from the original instrument. The adult expert committee modified the English wording for three of the 30 question items (10%; items 1, 4, 15 in Table 4) and one of the two instrument prompts. All changes made to the English wording were to either simplify the language or to add in contextually- and locally-relevant examples (see Table H in S1 Data for detailed reasoning).

**Table 4:** Cross-culturally adapted Child PTSD Symptom Scale for DSM-5 (CPSS-5) for Samoan adolescents with changes from the original CPSS-5 and pretesting results for understandability and honesty among n = 5 adolescents.

	Changes from the original CPSS-5	CPSS-5 adapted for Samoan adolescents		Pretesting*	
				% Comprehension* Is this question easy to understand?	% Honesty* Would adolescents answer honestly?
0.1	Sometimes scary or upsetting things happen in your life. It might be something like getting beaten up, living through a tsunami, witnessing violence at home, being touched in a way you didn’t like or that made you feel uncomfortable, having a parent get hurt or killed, or some other very upsetting event.	Sometimes scary or upsetting things happen in your life. It might be something like getting beaten up, living through a tsunami, witnessing violence at home, being touched in a way you didn’t like or that made you feel uncomfortable, having a parent get hurt or killed, or some other very upsetting event.	E iai taimi e tutupu ai mea e fefe ai pe e te faanoanoa ai i lou olaga. E pei o le fasi o oe, sao mai se galulolo sa tupu, molimauina o sauaga i totonu o le aiga, tagofia o oe i se auala na e le mana’o ai ma e le filemu ai, fa’amanu’alia o se matua pe ua maliu foi, poo se isi lava fa’alavelave matuia.	Agree: 80% (n = 4) Neutral: 20% (n = 1)	Agree: 60% (n = 3) Neutral: 40% (n = 2)
1	Please write down the scary or upsetting thing that bothers you the most when you think about it or the thing that you try not to think about (this should be the event you listed in the Trauma Screen):	Please write down the scary or upsetting thing that bothers you the most when you think about it or the thing that you try not to think about (this should be the event you listed in the Trauma Screen):	Fa’amolemole tusi i lalo le fa’afitauli na pito sili ona e fefe ai, pe sa tele ina fa’afatu’ulu (e.g., fa’alavelave) ia te oe, pe a e mafaufau iai, poo se mea oloo e taumafai e aua e te mafaufau iai (o le fa’alavelave lea na ‘e lisia i le Iloiloga o ni a’afiaga talu ai se fa’afitauli matuia, pe afai na faaaoga Trauma Screen):		
2	No changes	When did it happen?	O anafea na tupu ai?		
3	No changes	These questions ask about how you feel about the upsetting thing you wrote down. Read each question carefully. Then circle the number that best describes how often that problem has bothered you IN THE LAST MONTH. Not at all Once a week or less/a little 2 to 3 times a week/somewhat 4 to 5 times a week/a lot 6 or more times a week/almost always	O fesili nei o lo’o fesiligia ai ou fa’alogona ina ua e tusia le mea sa e faanoanoa ai. Faitau lelei fesili taitasi. Li’o le fuainumera e te iloa o lo’o faamatala lelei mai ai pe na faafia ona faafatu’ulu ia te oe lena faalavelave na tupu I LE MASINA UA TE’A. E leai lava E faatasi i le vaiaso pe le tele fo’i E faalua pe faatolu i le vaiaso/e feoloolo lava E faafa pe faalima i le vaiaso/e tele taimi E faaono pe sili atu i le vaiaso/toe lava o taimi uma		
4	Having upsetting* thoughts or pictures about it that came into your head when you didn’t want them to *Upsetting means that they made you feel unhappy or worried	Having upsetting* thoughts or pictures about it that came into your head when you didn’t want them to *Upsetting means that they made you feel unhappy or worried	Iai fa’alogona le fiafia* po’o ni ata fa’atatau i ia fa’alogona e o’o mai i lou mafaufau ae e te le’i mana’o ai *O faalogona le fiafia ua faauigaina na o’o ai ina e fa’anoanoa pe popole		
5	No changes	Having bad dreams or nightmares	Faia ni miti lē lelei poo miti taufaafefe	Agree: 100% (n = 5)	Agree: 100% (n = 4**)
6	No changes	Acting or feeling as if it was happening again (seeing or hearing something and feeling as if you are there again)	Fa’atinoga po’o ni fa’alogona pei ua toe tupu fo’i (vaaia pe lagona se mea ma fa’alogona pei ua toe tupu fo’i)	Agree: 100% (n = 5)	Agree: 100% (n = 5)
7	No changes	Feeling upset when you remember what happened (for example, feeling scared, angry, sad, guilty, confused)	Fa’alogona lē fiafia pe a e toe manatua se mea na tupu (fa’ata’ita’iga, fa’alogona fefe, ita, fa’anoanoa, ta’usala, lē mautonu)	Agree: 100% (n = 5)	Agree: 100% (n = 5)
8	No changes	Having feelings in your body when you remember what happened (for example, sweating, heart beating fast, stomach or head hurting)	Iai fa’alogona o lou tino pe a toe manatua se mea na tupu (fa’ata’ita’iga, afu, vave le tātā o le fatu, tigā le manava po’o le ulu)	Agree: 80% (n = 4) Neutral: 20% (n = 1)	Agree: 80% (n = 4) Neutral: 20% (n = 1)
9	No changes	Trying not to think about it or have feelings about it	O loo taumafai e aua le mafaufau i ai, pe iai ni lagona e faatatau i ai	Agree: 100% (n = 5)	Agree: 100% (n = 5)
10	No changes	Trying to stay away from anything that reminds you of what happened (for example, people, places, or conversations about it)	Taumafai e ‘alo ese mai so’o se mea e toe fa’amanatu atu ai ia te oe le mea na tupu (fa’ata’ita’iga, tagata, nofoaga, po’o se talanoaga fa’atatau iai)	Agree: 100% (n = 5)	Agree: 100% (n = 5)
11	No changes	Not being able to remember an important part of what happened	Lē mafai ona toe manatua se vaega taua o le mea na tupu	Agree: 100% (n = 5)	Agree: 100% (n = 5)
12	No changes	Having bad thoughts about yourself, other people, or the world (for example, “I can’t do anything right”, “All people are bad”, “The world is a scary place”)	Fa’alogona leaga fa’atatau ia te oe lava, isi tagata, po’o le lalolagi (fa’ata’ita’iga, “E leai se mea sa’o ou te faia”, “O tagata uma e leaga”, “O le lalolagi o se nofoaga taufa’afefe”)	Agree: 100% (n = 5)	Agree: 100% (n = 5)
13	No changes	Thinking that what happened is your fault (for example, “I should have known better”, “I shouldn’t have done that”, “I deserved it”)	Mafaufauga o oe e mafua ai le mea na tupu (fa’ata’ita’iga, “Sa tatau ona ou iloa lelei”, “Sa lē tatau ona ou faia”, “O le mea lena ou te maua”)	Agree: 100% (n = 5)	Agree: 100% (n = 5)
14	No changes	Having strong bad feelings (like fear, anger, guilt, or shame)	Malosi ni lagona lē lelei (pei o le fefe, ita, lagona le sesē, po’o le maasiasi)	Agree: 100% (n = 5)	Agree: 100% (n = 5)
15	Having much less interest in doing things you used to do (for example, spending time with friends, playing games, etc.)	Having much less interest in doing things you used to do (for example, spending time with friends, playing games, etc.)	Ua itiiti atu le fiafia e fa’atino ni galuega sa masani ai (fa’ata’itaiga: evaga ma uo, ta’aloga)	Agree: 100% (n = 5)	Agree: 100% (n = 5)
16	No changes	Not feeling close to your friends or family or not wanting to be around them	Lē lagonaina le vavalalata i uo ma aiga pe le fiafia fo’i e fa’atasi ma i latou	Agree: 100% (n = 5)	Agree: 60% (n = 3) Neutral: 20% (n = 1) Disagree: 20% (n = 1)
17	No changes	Trouble having good feelings (like happiness or love) or trouble having any feelings at all	Faigatā ona maua ni lagona lelei (pei o le fiafia po’o le alofa) pe faigatā ona maua ni lagona	Agree: 100% (n = 5)	Agree: 100% (n = 5)
18	No changes	Getting angry easily (for example, yelling, hitting others, throwing things)	Maitaita gofie (fa’ata’ita’iga, e’ē, pa’ilima i isi, taua’i solo ni mea)	Agree: 100% (n = 5)	Agree: 100% (n = 5)
19	No changes	Doing things that might hurt yourself (for example, taking drugs, drinking alcohol, running away, cutting yourself)	Faia o ni mea e ono a’afia ai oe (fa’ata’ita’iga, tagofia o fuala’au fa’asaina, inu ava malosi, sola ese, tasele pe fa’amanu’aina oe lava)	Agree: 100% (n = 5)	Agree: 80% (n = 4) Neutral: 20% (n = 1)
20	No changes	Being very careful or on the lookout for danger (for example, checking to see who is around you and what is around you)	Fa’aete’ete tele po’o le va’ava’ai toto’a pe iai se tulaga faigata poo se faalavelave (e ono tupu) (fa’ata’ita’iga, siakiina po’o ai o i ou autafa, ae po’o a ni mea o siomia ai oe)	Agree: 80% (n = 4) Neutral: 20% (n = 1)	Agree: 80% (n = 4) Neutral: 20% (n = 1)
21	No changes	Being jumpy or easily scared (for example, when someone walks up behind you, when you hear a loud noise)	Mate’ite’i pe fefe gofie (fa’ata’ita’iga, pe a savali atu i ou tua se tagata, pe a lagonaina se pa’ō leotele)	Agree: 80% (n = 4) Disagree: 20% (n = 1)	Agree: 80% (n = 4) Neutral: 20% (n = 1)
22	No changes	Having trouble paying attention (for example, losing track of a story on TV, forgetting what you read, unable to pay attention in class)	Faafaigata ona ua’i le mafaufau (fa’ata’ita’iga, lē mulimuli lelei i se tala [o matamata ai] i le TV, galo se mea na e faitau iai, le mafai ona ua’i le fa’alogo i le aoga)	Agree: 100% (n = 5)	Agree: 100% (n = 5)
23	No changes	Having trouble falling or staying asleep	Faigatā ona moe pe faaauau le moe	Agree: 100% (n = 5)	Agree: 100% (n = 5)
0.2	No changes	Have the problems above been getting in the way of these parts of your life IN THE PAST MONTH? Yes/No	Faamata na avea faafitauli (o ta’ua) i luga atu ma faalavelave i vaega nei o lou olaga i le MASINA UA TUANA’I? Ioe/Leai	Agree: 80% (n = 4) Neutral: 20% (n = 1)	Agree: 80% (n = 4) Neutral: 20% (n = 1)
24	No changes	Fun things you want to do	O mea faafiafia (loto) e te mana’o e fai		
25	No changes	Doing your chores	Faiga o au feau	Agree: 100% (n = 5)	Agree: 100% (n = 5)
26	No changes	Relationships with your friends	Va ma au uo	Agree: 80% (n = 4) Disagree: 20% (n = 1)	Agree: 80% (n = 4) Neutral: 20% (n = 1)
27	No changes	Praying	Tatalo	Agree: 100% (n = 5)	Agree: 100% (n = 5)
28	No changes	Schoolwork	Meaaoga	Agree: 100% (n = 5)	Agree: 100% (n = 5)
29	No changes	Relationships with your family	Va ma lou aiga	Agree: 100% (n = 5)	Agree: 100% (n = 5)
30	No changes	Being happy with your life	Fiafia i lou olaga	Agree: 80% (n = 4) Neutral: 20% (n = 1)	Agree: 80% (n = 4) Neutral: 20% (n = 1)

*We merged responses of “strongly agree” and “agree” together and responses of “strongly disagree” and “disagree” together (full results in Table H in S1 Data). **One participant did not respond.

Pretesting identified two items (items 21 and 26) that might be challenging for adolescents to understand (Table 4). Though for 28 of the items (out of 30; 93%), no participant raised concerns about understandability, there were two items (7%) that received at least one response of “disagree” or “strongly disagree” to whether the item is sufficiently understandable (items 21 and 26), though no qualitative explanations were provided. Based on this, we discussed items 21 and 26 in the focus group, which revealed a small grammar edit to the item 26 translation, but otherwise there was consensus to leave the items unchanged.

For pretesting whether adolescents would report honest answers, no participants reported disagreement for 29 items (out of 30; 97%). Ony one item (3%) received at least one response of “disagree” or “strongly disagree” to whether adolescents would report honest answers (item 16). Two qualitative responses were provided with further reasoning (Table J in S1 Data), one of which involved fear of sharing negative information about their families: “*I feel like most teens would lie so that others would think that they have a healthy relationship with their families*”. This issue was discussed further in the focus group.

### Deliberate self-harm questionnaire

Table 5 outlines the suicide questionnaire developed for Samoan adolescents. The majority (67%) of questions were copied verbatim from the ALSPAC questionnaire (12 questions and the instrument prompt; items 1, 2, 6–8, 10–13, 16–18); three items (17%; items 3–5) were copied from the SITBI and one question was adapted from it (item 9; Table I in S1 Data). We developed two new questions to capture history of suicide attempts (items 14 and 15), as we were interested in developing instruments that could measure frequency of attempts and age of first attempt; a measure that was not included in existing instruments. See Table I in S1 Data for detailed breakdown of question adaptations.

**Table 5:** Cross-culturally developed deliberate self-harm questionnaire for Samoan adolescents (based on the ALSPAC and SITBI questionnaires) with pretesting results for understandability and honesty among n = 5 adolescents.

	Suicide Questionnaire Developed For Samoan Adolescents				Pretesting*	
					% Comprehension Is this question easy to understand?	% Honesty Would adolescents answer honestly?
0	Life has many ups and downs. Sometimes people feel upset. These feelings can be so bad that people who have them may feel suicidal or want to self-harm. The following questions ask you about your feelings and the feelings of people close to you. We know this is a sensitive subject, but it is important to ask about it now, as it is not uncommon. By finding out about self-harm we can find ways of helping people.		E tele taimi e lūga lālo ai le olaga. O isi taimi e lagona ai e tagata le lē fiafia. O nei lagona e iai taimi e matuā tugā ai, ma o’o ai i se tulaga e lagona ai e e ua aafia ai, le fia pule i le ola, pe faamanu’alia o ia lava. O fesili o i lalo, o le a fesiligia ai ou faalagona, ma lagona o tagata e latalata ia te oe. Matou te iloa e ma’ale’ale lenei mataupu ae taua pe a tatou fa’asoa i ai,āua e le o se mea fou. I le suesueina o le mataupu i le faamanu’alia o lou lava tagata, e mafai ai ona tatou maua ni auala e fesoasoani ai i ia tagata.		Agree: 80% (n = 4) Neutral: 20% (n = 1)	Agree: 100% (n = 5)
*SOCIAL NETWORKS*						
1	Has anyone in your family (not including yourself) ever hurt themselves on purpose (e.g. by taking an overdose of pills, or by cutting themselves)? Yes/No		Sa iai se isi o le tou aiga (e le faitauina ai oe) na faamanu’alia e ia ia lava (faataitaiga, so’ona inu fuala’au po o le selei/tatipi o le tino)? Ioe/Leai			
2	Have any of your close friends ever hurt themselves on purpose? Yes/No		E iai ni au uo mamae na faamanu’alia ma le mautinoa ia latou lava? Ioe/Leai		Agree: 100% (n = 5)	Agree: 100% (n = 5)
3	Has anyone you have ever known died by suicide? If so, how many?		E iai se isi e te iloa na oti ona o le pule i le ola? Afai e iai, e to’afia?		Agree: 100% (n = 5)	Agree: 100% (n = 5)
*SUICIDAL THOUGHTS*						
4	How old were you the first time you had thoughts of killing yourself?		E fia ou tausaga i le uluai taimi na e mafaufau ai e te pule i lou ola?		Agree: 100% (n = 5)	Agree: 100% (n = 5)
5	How old were you the last time?		E fia ou tausaga i le taimi mulimuli na tupu ai?			
*SELF-HARMING BEHAVIORS*						
6	Have you ever hurt yourself on purpose in any way (e.g. by taking an overdose of pills, or by cutting yourself)? Yes/No		Na iai se taimi na e faamanu’alia ai ma le mautinoa oe lava i so’o se auala (pei o le so’ona inu o fualaau po’o le tatipi o lou tino)? Ioe/Leai		Agree: 80% (n = 4) Disagree: 20% (n = 1)	Agree: 80% (n = 4) Disagree: 20% (n = 1)
7	*If yes*:		*Afai e ioe*:		Agree: 80% (n = 4) Disagree: 20% (n = 1)	Agree: 80% (n = 4) Disagree: 20% (n = 1)
		How many times have you done this in the last year? Please mark one box only. Once 2–5 times 6–10 times More than 10 times		E faafia ona e faia lea tulaga i le tausaga ua tuana’i? Faamolemole, maka na’o le pusa e tasi. Faatasi 2–5 6–10 Sili atu ma le 10		
8		When was the last time you hurt yourself on purpose? Please mark one box only. In the last week More than a week ago but in the last year More than a year ago		O le a le taimi mulimuli na e faamanu’alia ai ma le mautinoa oe lava? Faamolemole, maka na’o le pusa e tasi. Vaiaso ua te’a Ova atu ma le vaiaso ua tuana’i Sili atu ma le tausaga	Agree: 80% (n = 4) Disagree: 20% (n = 1)	Agree: 60% (n = 3) Neutral: 20% (n = 1) Disagree: 20% (n = 1)
9		How old were you the last time you hurt yourself on purpose?		E fia ou tausaga i le taimi mulimuli na e fuafuaina ai e faamanu’alia oe lava?	Agree: 80% (n = 4) Neutral: 20% (n = 1)	Agree: 60% (n = 3) Neutral: 20% (n = 1) Disagree: 20% (n = 1)
10		The last time you hurt yourself on purpose, which of the actions below best describes what you did? Please mark all boxes that apply. Swallowed pills or something poisonous Cut or carved skin Burned your skin (i.e., with a cigarette, match or other hot object) Inserted sharp objects into your skin or nails Picked areas of your body to the point of drawing blood Hit yourself on purpose Scraped your skin to the point of drawing blood Something else > Please say what		I le taimi mulimuli na e faamanu’alia ai ma le mautinoa oe lava, o a gaoioiga o loo lisi atu i lalo e faamatalā lelei le mea sa e faia? Faamolemole maka uma pusa e fetaui. Inu ni fualaau poo se mea oona Tatipi le pa’u o le tino Susunu lou tino (i se sikaleti, afitusi, poo se isi mea vevela) Sulu ni mea maai i le pa’u o lou tino poo atigilima Iini vaega o lou tino se’ia maligi le toto Ta oe lava ma le mautinoa Vavalu ese le pa’u o le tino se’ia maligi le toto Se isi mea > Faamolemole faailoa mai poo le a	Agree: 80% (n = 4) Disagree: 20% (n = 1)	Agree: 80% (n = 4) Disagree: 20% (n = 1)
11		Do any of the following reasons help to explain why you hurt yourself on that occasion? Please mark all boxes that apply. I wanted to show how desperate I was feeling I wanted to die I wanted to punish myself I wanted to frighten someone I wanted to get relief from a terrible state of mind Some other reason > Please say what		E iai se mafuaaga o ta’ua i lalo e fesoasoani e faamatala le mafuaaga na e faamanu’alia ai oe lava i lena taimi? Faamolemole maka le pusa e talafeagai. Na ou mana’o e faaali o’u lagona fia maua se lavea’i Na ou fia oti Na ou mana’o e fa’asala a’u Na ou mana’o e faafefe se isi Na ou mana’o ou te mapu mai mafaufauga le lelei Nisi mafuaaga > Faamolemole faailoa mai	Agree: 80% (n = 4) Neutral: 20% (n = 1)	Agree: 80% (n = 4) Neutral: 20% (n = 1)
12		After you had hurt yourself on that occasion, how did you feel? Please mark one box only. Better than before The same as before Worse than before		Ina ua mae’a ona faamanu’alia oe lava i lena taimi, o le a sou lagona na i ai? Faamolemole maka le pusa e tasi. Sili atu nai lo le taimi muamua Tutusa ma le taimi muamua Leaga atu i lo le taimi muamua	Agree: 80% (n = 4) Neutral: 20% (n = 1)	Agree: 60% (n = 3) Neutral: 20% (n = 1) Disagree: 20% (n = 1)
*SUICIDE ATTEMPTS*						
13	Have you ever made plans to kill yourself (for example, a specific plan towards where, when, and how)?		Na iai se taimi na e fuafua ai e te pule i lou ola (mo se faataitaiga, o le a sau fuafuaga na tapena i le nofoaga, le taimi ma le aso, ma e faapefea fo’i ona faataunuu)?		Agree: 100% (n = 5)	Agree: 100% (n = 5)
14	How many times did you try to kill yourself or make a suicide attempt?		E faafia ona e taumafai e fasioti oe, pe taumafai foi e pule i lou ola?		Agree: 80% (n = 4) Neutral: 20% (n = 1)	Agree: 80% (n = 4) Neutral: 20% (n = 1)
15	What age(s) were you when you made your suicide attempt(s)?		O le fia o ou tausaga na e taumafai ai e te pule i lou ola?		Agree: 80% (n = 4) Neutral: 20% (n = 1)	Agree: 80% (n = 4) Neutral: 20% (n = 1)
*HELP-SEEKING BEHAVIORS*						
16	The last time you hurt yourself in any way (e.g. by taking an overdose of pills, or by cutting yourself) did you seek medical help/first aid from any of the following? Please mark all boxes that apply. Crisis phone line Reaching out to a friend Reaching out to a trusted adult GP (family doctor) Mental health professional Hospital emergency department Other > Please say who this was		I le taimi mulimuli na e faamanu’alia ai oe lava i soo se auala (faataitaiga soona inu fualaau, poo le tatipi o lou tino, na e saili mo se fesoasoani i le falema’i, poo se isi lava pei ona lisi atu i lalo? Faamolemole maka pusa e talafeagai. Telefoni laina fesoasoani Fesoota’i i se uo Fesootai i se tagata matua faatuatuaina Foma’i (a le aiga) Tagata tomai i le maloloina o le mafaufau Matagaluega o ma’i faafuase’i i le Falema’i Isi > Faamolemole fa’ailoa mai poo ai lea		Agree: 80% (n = 4) Neutral: 20% (n = 1)	Agree: 60% (n = 3) Neutral: 40% (n = 2)
17	Have you ever tried to get help from someone or somewhere about hurting yourself on purpose, or about wanting to kill yourself? Yes/No		Na iai se taimi na e taumafai e saili se fesoasoani mai se isi po o se nofoaga e uiga i lou faamanu’alia o oe lava, poo le fia pule fo’i i le ola? Ioe/Leai		Agree: 100% (n = 5)	Agree: 100% (n = 5)
18	*If yes*:		*Afai e ioe*:		Agree: 100% (n = 5)	Agree: 100% (n = 5)
		Who have you been to for help? Please mark all boxes that apply. Mother Father Brother Sister Someone else in your family A friend A teacher High school counselor Peer mentor A GP (family doctor) A social worker A psychologist or psychiatrist A telephone crisis line Drug dealer Social media Sports coach Somewhere else (e.g. internet, book, magazine, other person, etc.) Other trusted adult > Please say what or who		O ai na e o’o i ai mo se fesoasoani? Faamolemole maka uma pusa e talafeagai. Tina Tama Tuagane Tuafafine Se isi o lou aiga Uo Faia’oga Faufautua Aoga Maualuga Fesoasoani augatupulaga Foma’i a le aiga Tagata i le galuega tauagafesootai Tagata tomai tau saikolo ma le foma’i o le mafaufau Laina telefoni Fesoasoani Tagata faatau fualaau faasaina Ala o faasalalauga a tagata faitele Faiaoga tau taaloga Se isi fesoasoani (faataitaiga Initaneti, tusi, mekasini, isi) Se isi tagata matua Faatuatuaina > Faamolemole faailoa mai poo le a poo ai		

*We merged responses of “strongly agree” and “agree” together and responses of “strongly disagree” and “disagree” together (full results are in Table I in S1 Data).

Pretesting with adolescents identified four items that might be challenging to understand (Table 5). Although no participants raised concerns regarding understandability for 14 of the items (out of 18; 78%), four items (22%) received at least one response of “disagree” or “strongly disagree” as to whether the item was sufficiently understandable (items 6, 7, 8, and 10). Based on this, we discussed items 6, 7, 8, and 10 in the focus group (Table I in S1 Data); adolescents expressed that the Samoan is harder to understand than the English, but after attempts to make it clearer, the group decided that no further changes would need to be made.

For honesty, no participants disagreed that adolescents would respond honestly for 11 of the items (out of 18; 61%). Six items (22%) received at least one response of “disagree” or “strongly disagree” to whether adolescents would be honest (items 6–10, 12). Specifically, the questions related to social networks, suicidal thoughts, suicide attempts, and help-seeking behaviors had complete agreement that adolescents would be honest; but nearly all of the questions related to self-harming behaviors (6/7; 86%) had at least one adolescent report concerns about honesty. Only one qualitative reason was provided (Table J in S1 Data): “*Scared of expressing themselves*”. Based on this, we took the same approach as other instruments and centered focus group discussions around how to make adolescents feel safe being honest.

### Development of administrative instructions

Adolescents in the focus group corroborated the pretesting survey data, expressing that adolescents would be less honest for a variety of sensitive questions concerning sexual trauma, physical punishment, violence at home, and communicating stigmatized feelings such as sadness, irritation, and shame. Adolescents provided suggestions to administer the survey in a way to promote honesty. Two major recommendations arose: (1) build a safe environment to make the adolescent feel comfortable; and (2) explain and communicate intentions behind the questionnaire. To better promote adolescent comfort, we developed instructions for survey administrators to implement prior to delivering the questionnaires (Table 6; also in S1–S4 Texts).

**Table 6:** Evidence-based administrative instructions to promote honesty developed for the PHQ-9M, GAD-7, CPSS-5, Trauma Screen, and Deliberate Self-Harm Questionnaire for Samoan adolescents.

Samoan adolescents might not answer sensitive topics in questionnaires honestly. To promote honesty, before administering this questionnaire, please make an effort to build trust and create a safe space for the adolescent. Suggestions include: • It is ideal if the questionnaire is administered by a person the adolescent does not already know (such as a stranger) and also speaks and understands the questionnaire in both English and Samoan to assist the adolescent to answer completely and truthfully. Administer the questionnaire in a private space, with either just you and the adolescent, or the adolescent alone. • Before administering the questionnaire, take time to build trust and connection. This could include: ⚬ Asking the adolescent questions about their interests and actively listening to their answers ⚬ Employing a non-judgmental and warm demeanor ⚬ Being present, which includes giving the adolescent your full attention ⚬ Explaining that any information shared will be held confidential, and clearly communicating which instances under mandatory reporting requirements (if any) would require reporting information to their families • Clearly communicate the intention behind the questionnaire (such as, to understand how common certain mental health problems are or to understand what you are going through to help you feel better). Clearly communicate that the goal of asking them these questions is not to get them or anyone they know in trouble. • Provide the adolescent the opportunity to ask questions before they begin the questionnaire. E ono lē tali sa’o e tupulaga Samoa ni mataupu ma’ale’ale i pepa fesili. Ina ia u’unaia ona tali mai ma le faamaoni, faamolemole taumafai i se faiga e faatuatuaina ai ma iloa ai e tupulaga e saogalemu a latou faamatalaga ia te oe. E mafai ona aofia ai fautuaga nei: • E pito sili pe afai e faatautaia le pepa fesili e se isi latou te lē iloa (e pei o se tagata ese), ma e tautala ma malamalama i le Pepa Fesili i le Igilisi ma le faa-Samoa, ina ia fesoasoani i le talavou ia atoatoa ma faamaoni a latou tali. • Ia faatautaia le taliga o le pepa fesili i se nofoaga e le o tatalaina i le lautele, e na ‘o oulua ma le talavou, pe na o ia fo’i. • A o le’i faatumua le pepa fesili, fai se lua taimi ia tupu ai lona faatuatuaina o oe ma fesoota’i lelei atu. E mafai ona aofia ai: ⚬ Lou fesili i ai i mea latou te fiafia i ai ma matuā faalologo lelei i a latou tali ⚬ Ia faaalia lou lē faamasino tagata ma ni ou uiga mafanafana ⚬ Ia iai ma latou, e aofia ai ma le tuu atoa i ai o lou loto i lou taimi ma le talavou ⚬ Faamalamalama i ai o soo se faamatalaga e tuu atu e le faailoā i se isi, ma ia manino lelei ni taimi (pe a iai) e ono lipoti ai ia faamatalaga i lona aiga • Ia faailoa manino le mafuaaga o le pepa fesili (e pei o le fia malamalama poo le a le taatele o nisi o faafitauli tau le maloloina o le mafaufau o alia’e, poo le malamalama i se tulaga o e iai ina ia iloa le auala sili e fesoasoani atu ai ia suia i le lelei ou lagona). Ia manino ona faailoa atu, o le faamoemoe o fesili e lē ina ia aafia ai ia poo se isi latou te iloa. • Tuu se avanoa i le talavou e fai mai ni fesili ae le’i amata ona tali le pepa fesili.

Participants recommended administering the questionnaire in a private space: “*maybe he or she will feel comfortable telling only one person*, *like [translation*: *should be one person to ask the question in private]*”. Participants also recommended that the administrator should attempt to build trust before administering the survey, as: “*maybe before giving out the surveys*, *maybe try to earn their trust first*, *like try to get to them [to] feel safe to give out those kinds of personal information*”. This could include the adult administrator asking the adolescent questions about their interests, employing a non-judgmental, warm, and present demeanour, and actively listening. Some adolescent participants gave specific demographic examples for administrators who would naturally elicit more trust, including someone who is an outsider, such as ‘*not the teacher*’ or someone who identifies as Fa’afine (meaning, a person who identifies as non-binary) “*[because] they make us very feel very comfortable*”. Participants also stated the importance of building trust. Participants also recommended that administrators clearly communicate that the information gathered would be confidential: “*maybe kind of telling them you guys will keep that as a secret [and] not telling anyone about what [their] answer is*”. This included clearly explaining what circumstances or situations would require sharing disclosed information with their families.

Participants also recommended clearly communicating the purpose of the questionnaire so that adolescents understand why it is important to answer honestly, as: “*translation*: *understand and know what the main purpose of the survey [is]*”. Adolescents also recommended that administrators communicate that the purpose of the questionnaire is not to get them or someone they know in trouble. This was especially applicable for the questions concerning sexual trauma, as there was concern that the goal of the questionnaires would be to reprimand someone in the adolescent’s network. Participants also shared that the mode of delivery (paper versus electronic) would not make a difference in the level of honesty.

## Discussion

This paper presents the cross-cultural adaptation process for five mental health screening instruments for Samoan adolescents. We employed a rigorous, comprehensive process involving an expert committee, group-based professional forward translation, back-translation, and pretesting with adolescents using an online survey and focus group. Though many changes were made to the original wording, these changes were largely clarifications to help with understandability and provide locally relevant examples. We do not believe that our changes compromise the original intent of the instruments.

We were surprised at the degree of trouble among the adolescents to achieve consensus on the Samoan translations across instruments. We feel this is likely an artifact of the ongoing development of the Samoan language and increased reliance on English for this generation of adolescents, especially given the rapid exposure of social media use exposing adolescents to content created in English in the continental United States or elsewhere. Based on this, we feel that maintaining the professional translations would be sufficient in protecting cross-cultural validity.

Though these instruments were developed in partnership with adolescents and experts living in the US territory of American Samoa, we believe that the English language edits made should maintain continuity in other Samoan populations. This should include adolescents living in the independent state of Samoa and in other parts of the global Samoan diaspora. It is possible, however, that the Samoan translations might not generalize to other geographic locations with other external influences, and so further adaptation of the Samoan translations might be needed in other settings. One additional benefit of adapting these instruments in American Samoa is that our collaborators and participants had a firm grasp of both English and Samoan languages, as well as Samoan and continental United States cultures (as having an adaptation team that is bilingual and bicultural is most advantageous for cross-cultural adaptation [22]). American Samoa might therefore serve as a natural middle ground between Samoan and Western cultures, making the instruments adapted in this context to be most generalizable to other Samoan communities globally.

The back-translation process was critical to our efforts and informed several modifications, likely due to the subjective nature of the Samoan language. Though back-translation helps researchers make inferences on the quality of the translation by amplifying any mistakes present in the forward translation [22], several guidelines for cross-cultural instrument adaptation do not require this step [22]. In fact, one experimental study demonstrated that the addition of a back-translation generated moderate additional impact to an expert committee, making it a methodologically less critical [33]. Some even argue that back-translation might even negatively impact the translation quality, as the forward translators might opt to produce translations that would be better suited for back-translation, but this could be at the expense of using optimal wording in the target language [22,34]. Back-translation could also prevent the use of meaningful and appropriate modifications of content for cultural reasons [34]. For cross-cultural adaptation of questionnaire tools in Samoan, and possibly other Polynesian languages, back-translation might provide additional benefit compared to other language contexts. Our results also demonstrated the critical need to present both English and Samoan side-by-side for cross-culturally adapted instruments for Samoan contexts, given the subjective interpretations of the language alongside challenges for adolescents in reading more formal and advanced presentations of the language.

We did not completely follow the Gjersing recommendations [21] and have some steps remaining to complete (such as investigations of operational equivalence and exploratory and confirmatory analyses). For example, we did not involve a third translator to synthesize the forward translations from two independent translators, but instead used a locally developed consensus- and group-based synthesis method. We felt this would be acceptable given that previous research has recommended that authors should adapt the process to what works best for the specific context [22]. We also did not synthesize our back-translation from two different back-translators, but instead only visually compared back-translations from one colleague. One review article demonstrated that there is no real consensus on methods for cross-cultural adaptation of questionnaires, but did conclude that the expert committee and multiple translators to be the most prominent factors in contributing to comparable results [22]. Taken together, we feel that our deviations from these guidelines would not greatly impact the psychometric properties of the instruments.

The next steps for these instruments should include an assessment of cross-cultural validity, such as measurement equivalence [22]. This could include piloting the instruments through large-scale surveys to examine internal consistency, construct validity, and responsiveness, and additionally conducting assessments of clinical utility to assess diagnostic accuracy, including identifying optimal cut-off scores, instrument responsiveness, and clinically meaningful changes, which might vary on the patient population [35]. Since we did not remove any items from the instruments, but just adapted them to be more suitable for this context, we would recommend applying the same numerical thresholds for clinical decision making; though future research is needed to reassess if the validity of these thresholds remain. Further work should include evaluations on the validity and reliability of the final instruments, which could include factor analyses to identify if certain items could be removed for parsimony (for example, certain CPSS-5 Trauma Screener items might be so uncommon in this context–such as the item “*being around a war*”–that they might not be worthwhile to collect). Similarly, it would be important to understand whether, in lowering the screening threshold for some items like physical trauma to try and account for normative practices, we are inadvertently underestimating the mental health impact of such practices.

Our methodological approach had several strengths and weaknesses. Strengths include that we took a careful, consensus-based, and community-partnered approach in the adaptation of these instruments, which aligns with recommendations for cross-cultural adaptation [22] and from other researchers conducting instrument adaptations for the Samoan language [23]. We also drew from a team of six translators for the forward and back-translation process, which is said to prevent bias and help achieve equivalence [22]. In addition to pretesting concerns with comprehension, we also systematically pretested honesty to assess the degree of underreporting for a variety of sensitive topics. Our data suggests that we should expect the underreporting of items related to particularly sensitive and stigmatized subject matter in this setting (especially for self-harming behavior, sexual abuse, domestic violence, and feelings of sadness, irritation, and shame), and this information can add value to aid in the interpretation of how underreporting could potentially bias future data.

There were several weaknesses, however. Our pretesting only included a small sample of adolescents (n = 6 for the survey–but only n = 5 for most items due to technical difficulties, and n = 5 for the focus group). While we did not record demographic characteristics due to confidentiality concerns, these participants may have included those of higher socioeconomic status, given that access to a computer and internet access was required to participate (although both have become more widespread in American Samoa since COVID-19-related online learning has been introduced), and might not be representative of the adolescent population in American Samoa. We were also explicit in communicating to the participants that a team of adult experts generated an initial draft of the instruments and their translations; however, given that the focus group was conducted by adult team members, it is possible that some adolescents were shy to object to instrument wordings, given the elements of age hierarchies and power dynamics at play, as it is custom for American Samoan adolescents to defer decision-making to people who are older than them as a form of respect. We feel this was unlikely, however, as the use of adult facilitators was considered the most culturally appropriate method and intentional efforts were taken to breakdown hierarchies–such as through ice breaker games.

## Conclusions

This paper provides five cross-culturally adapted self-administered screening instruments for common mental health conditions for Samoan. Following additional testing, these instruments may have utility for screening and research purposes. This could include implementation within clinical in- and out-patient services, school-based screening programs, or future school-based surveys to identify prevalence estimates to inform policy and practice. Further research is needed to quantitatively assess the validity and reliability of the application of these tools, as well as evidence of generalizability for use among other Samoan populations globally.

## Supporting information

S1 Data Supplemental tables. This file contains additional study information presented in detailed tables.
(PDF)

S1 Text PHQ-9M for Samoan adolescents. This file presents the adapted PHQ-9M questionnaire for Samoan adolescents, reformatted to align with the original screening instrument to facilitate administration.
(PDF)

S2 Text GAD-7 for Samoan adolescents. This file presents the adapted GAD-7 questionnaire for Samoan adolescents, reformatted to align with the original screening instrument to facilitate administration.
(PDF)

S3 Text CPSS-5 and Trauma Screen for Samoan adolescents. This file presents the adapted CPSS-5 and Trauma Screen questionnaire for Samoan adolescents, reformatted to align with the original screening instrument to facilitate administration.
(PDF)

S4 Text Deliberate self-harm questionnaire for Samoan adolescents. This file presents the adapted deliberate self-harm questionnaire for Samoan adolescents, reformatted to align with the original screening instrument to facilitate administration.
(PDF)
