# Supplementary material for: Cross-cultural adaptation of mental health screening instruments for Samoan adolescents
Source: PLOS Ment Health. 2025 Feb 11;2(2):e0000106. doi: 10.1371/journal.pmen.0000106 (PMC12798219; doi:10.1371/journal.pmen.0000106)
Supplement: S1 Data — This file contains additional study information presented in detailed tables. (PDF) [file pmen.0000106.s001.pdf]

S1 SUPPLEMENTAL MATERIALS

	Topic	Example sub-questions
1	Opening question	Can you tell me a little bit about your thoughts on the state of mental health among adolescents in American Samoa?
2	Status of mental health	What are the most common mental health problems among adolescents in American Samoa, if any at all?
3	Signs and symptoms of mental distress	What are the signs that an adolescent in American Samoa is struggling with mental health that someone else might notice?
4	Predictors of mental health and illness	What do you think is causing adolescent mental health problems in American Samoa? What would make an adolescent have good mental health?
5	Initial point(s) of contact and care pathways	What paths do adolescents in American Samoa struggling with mental health problems follow in the course of their search for help?
6	Coping mechanisms for mental distress	Would you know of any coping mechanisms that adolescents struggling with mental health problems in American Samoa use to feel better?
7	Existing infrastructure	What services are available to help adolescents living with mental health problems on Island?
8	Barriers and facilitators to care	What might prevent an adolescent from using these resources? What might make it easier for an adolescent to use these resources?
9	Potential interventions	If you could create a program or a change of some kind, what do you think would most improve adolescent mental health in American Samoa, if any at all?
10	Session closing	Are there things you think we didn't cover today that are important about adolescent mental health that we should know about?

Table A. Semi-structured interview guide topics and sub-questions used in the adult qualitative interviews, as published in Mew et al., 2023 (1). Prompts were often not used verbatim and interviews were conversational in style. We used open-ended probes to increase the breadth and depth of responses.

Name	Gender	Positionality	Occupation	Credentials and Training
Joshua Naseri	Fa‘afafine	Samoan person raised in Independent Samoa and American Samoa. Lived in American Samoa for 10+ years at the time of facilitation.	Program Manager for the Obesity, Lifestyle and Genetic Adaptations Research Center in American Samoa	Held a Bachelor’s of Science with a major in Health Science and a minor in Health Systems Management at the time of the focus group.
Leiema Hunt	Female	Samoan woman raised in American Samoa. Lived in American Samoa for 25+ years at the time of facilitation.	Public Health Advisor, Office of Island Affairs, Division of Partnership Support, Centers for Disease Control and Prevention	Held a Master of Public Health and is certified in Public Health (CPH) at the time of the focus group.

Table B. Focus group facilitator positionalities.

	Topic	Example sub-questions
1	Understandability	• How can we make the survey questions easier to understand?<br>• Would reading the questions aloud help understanding?<br>• *(After presenting the item on a PowerPoint presentation – with the area of concern in bold)*<br>○ Sub-prompts: Any issue understanding the bolded question? Any issue understanding the question stem? How would you interpret this question?
2	Honesty	• How can we make adolescents feel more comfortable being honest? How can we create an environment so that adolescents will answer the survey questions honestly?<br>○ Sub-prompts included: Who should administer the survey? How many people should administer the survey? Should we write participant names on the survey? Who should collect the survey? Should teachers be in the room when the survey is administered? Should we have a person/people from off-island (ex: someone completely unconnected to you or your family) collect the surveys? Should we have an opening introduction to tell adolescents why it is important to be honest?<br>• Would reading the questions aloud influence honesty?<br>• *(After presenting the item on a PowerPoint presentation – with the area of concern in bold)*<br>○ Sub-prompts included: Can we reword the bolded question to encourage adolescents to answer more honestly? Would this question cause adolescents emotional distress while reading this in a survey?<br>• What are the kind(s) of questions that adolescents wouldn't be honest for?
3	Safety	• *(After presenting the item on a PowerPoint presentation – with the area of concern in bold)*<br>○ Would this question cause adolescents emotional distress while reading this in a survey?
4	Session closing	Would you have other suggestions as to how we should create or deliver these questions?

Table C. Adolescent focus group semi-structured interview guide topics and sub-questions. Prompts were often not used verbatim and interviews were conversational in style.

Instrument	Diagnostic capabilities	Ages	Evidence for validity and reliability among adolescents	Evidence for low resource applications	Justification for selection of instrument for Samoan context
PHQ-9M (2)	Diagnostic aid for Major Depressive Disorder and Dysthymia (3). Screener for depression and suicide risk (3).	Middle and high school students (4)	Moderate and good convergent validity with other well-established tools (5) and appears to be valid and reliable in a clinical setting (5).	Used in school-based surveys among adolescents with low mental health literacy (6).	We selected this tool because American Samoan health practitioners most commonly use the PHQ-9 to screen depression in adolescents and adults. Several clinicians requested that this tool be validated. This instrument also only takes less than five minutes to administer which makes it feasible given resource constraints (for instance, administration time and printing costs).
GAD-7 (7)	Diagnostic aid for Generalized Anxiety Disorder	Originally developed for adult populations (7) but also validated among adolescents (8)	Validated to show optimum sensitivity and specificity with good differentiation for mild and moderate cases (9).	Used for survey administration in low resource contexts (10).	We selected this tool because it is already used in clinical practice with adolescents at the American Samoa Department of Health Behavioral Health Services.
CPSS-V (and Trauma Screen) (11)	Diagnostic aid for Post-traumatic Stress Disorder	Ages 8-18 years (11)	“Excellent internal consistencies, good to excellent test–retest reliability, and good convergent validity and discriminant validity” (11)	Adapted in other languages including Korean, Russian, Indonesian and Nepalese (12, 13).	We selected this tool because it is validated for the DSM-5 and is relatively quick to administer (about 10 minutes). We also selected it because it came with a potentially traumatic event screener to measure childhood trauma.

Table D. Selected instruments with information on original authors, development history, diagnostic capabilities, and justification for use in a Samoan context.

	PHQ-9M for Samoan Adolescents				Justification and deliberation of English wording changes by the expert committee	Samoan translation and back-translation notes	Adolescent Pretesting		
							Survey (n=6)		Focus group (n=5)
	Original English	Tracked Changes	Final English	Final Samoan			*Is this question easy to understand?*	*Would adolescents answer this question honestly?*	
0	How often have you been bothered by each of the following symptoms during the past two weeks?<br><br>For each symptom put an “X” in the box beneath the answer that best describes how you have been feeling:<br><br>(0) Not at all<br><br>(1) Several days<br><br>(2) More than half the days<br><br>(3) Nearly every day	No changes.<br><br>No changes.<br><br>No changes.	How often have you been bothered by each of the following symptoms during the past two weeks?<br><br>For each symptom put an “X” in the box beneath the answer that best describes how you have been feeling:<br><br>(0) Not at all<br><br>(1) Several days<br><br>(2) More than half the days<br><br>(3) Nearly every day	I le lua vaiaso talu ai, e fa'afia ona e a'afia i auga ta’itasi nei?<br><br>Tusi se “X” i le pusa i lalo ane o le tali pito talafeagai e faamatala ai lou lagona:<br><br>(0) E leai ni auga<br><br>(1) Ni nai aso<br><br>(2) Sili atu ma le afa o aso<br><br>(3) Toeitiiti lava o aso uma	No changes made.	Back-translations aligned; no changes made to the professional translation.	Answer: n<br>Strongly disagree (total nonsense): 0<br>Disagree: 0<br>Neutral: 1<br>Agree: 1<br>Strongly agree (totally understandable): 4	Answer: n<br>Strongly disagree (totally dishonest): 0<br>Disagree: 1<br>Neutral: 0<br>Agree: 2<br>Strongly agree (totally honest/truthful): 3<br><br>Why might adolescents not be honest in answering this question?<br>*“They feel as they can't quite express their feelings to anyone or even a stranger.”*	Not discussed in focus group.
1	Feeling down, depressed, irritable, or hopeless?	Feeling sad~~down~~, ~~depressed,~~ irritable (for example, easily annoyed), or hopeless?	Feeling sad, irritable (for example, easily annoyed), or hopeless?	Lagona le faanoanoa, maitaita, (mo se faataitaiga, ita gofie) po ua leai se faamoemoe?	Replaced “down, depressed” with “sad’. Adult key informants in qualitative interviews (1) stated that many adolescents would not know what the word ‘depressed’ means and recommended using the term ‘sad’ instead.	Back-translations aligned; no changes made to the professional translation.<br><br>No equivalent word for ‘irritable’ in Samoan, just			Not discussed in the focus group.

					Major theme from qualitative data that adolescents channel their depression into fights/aggression, since reported to be more aligned with Samoan social norms: "*it fits into the culture better*." We considered replacing irritable with "irritable/angry"; based on this, we consulted an expert on cross-cultural tool adaptation who recommended only adding if it is a clinical feature of depression. Since we weren't sure, and this would deviate from other PHQ-9 adaptations, we decided to not change it as to not threaten the psychometric validity of the construct Instead, we added a definition for "irritable" to aid understandability for adolescents.	'easily angry', but expert committee concluded that this is sufficient (especially given that the English and Samoan will be presented side-by-side).			
2	Little interest or pleasure in doing things?	~~Little interest or pleasure in~~Not really interested in doing things or talking to people (such as not wanting to spend time with friends or participate in family or church activities)?	Not really interested in doing things or talking to people (such as not wanting to spend time with friends or participate in family or church activities)?	E faalefiafia tele i mea e fai poo le talanoa foi i tagata (e pei o le lē fia mafuta ma uo pe auai i mea e fai a le aiga poo le ekalesia fo'i)?	We reworded original prompt to: "Not really interested in doing things" to keep the English wording language as simple as possible.<br><br>Qualitative interviews with adult key informants (1) identified that signs and symptoms for adolescents undergoing depressive episodes are socially withdrawn, isolated, and suddenly quiet/reserved, which is considered highly non-normative within Samoan social norms. We modified this item to capture the concept of social withdrawal and added context-specific examples. We added a new component "or talking to people (such as not wanting to spend time with friends or	No changes made to the professional translation.	Answer: N<br>Strongly disagree (total nonsense): 0<br>Disagree: 0<br>Neutral: 1<br>Agree: 3<br>Strongly agree (totally understandable): 2	Answer: n<br>Strongly disagree (totally dishonest): 0<br>Disagree: 1<br>Neutral: 1<br>Agree: 2<br>Strongly agree (totally honest/truthful): 2<br><br>Why might adolescents not be honest in answering this question?<br>*"Some of them have gone through in those kind of matters but doesn't have the confident to share about it."*<br>*"They don't want to be seen as weak or vulnerable"*	Not discussed in the focus group.

					participate in family or church activities)" as these were examples of common signs of depression in our data.				
3	Trouble falling asleep, staying asleep, or sleeping too much?	No changes.	Trouble falling asleep, staying asleep, or sleeping too much?	Faigata ona moe, faigata ona faaauau le moe, pe moe so'o?	No changes made.	Removed brackets suggested by translation team; otherwise, identical to professional translations.	Answer | n<br>Strongly disagree (total nonsense) | 0<br>Disagree | 0<br>Neutral | 0<br>Agree | 3<br>Strongly agree (totally understandable) | 3	Answer | n<br>Strongly disagree (totally dishonest) | 0<br>Disagree | 0<br>Neutral | 0<br>Agree | 3<br>Strongly agree (totally honest/truthful) | 3	Not discussed in the focus group.
4	Poor appetite, weight loss, or overeating?	No changes.	Poor appetite, weight loss, or overeating?	Tau le manogi se ai, alu le tino (lusi); po'o le soonaʻai.	No changes made.	Translation for "poor appetite" translated to "no appetite" or "food doesn't smell good anymore". Samoan team members said it is the best translation without using a sentence to describe, but we added "tau" to the translation to qualify.	Answer | n<br>Strongly disagree (total nonsense) | 1<br>Disagree | 1<br>Neutral | 0<br>Agree | 1<br>Strongly agree (totally understandable) | 3	Answer | n<br>Strongly disagree (totally dishonest) | 0<br>Disagree | 0<br>Neutral | 0<br>Agree | 2<br>Strongly agree (totally honest/truthful) | 4	Discussed; asked adolescents: "*Any issue in understanding this question?*"<br><br>One response: "*I also like the questions but there are some kids who aren't really able to speak English properly here. So I think it's easier for them…I prefer eating disorders and appetite.*"<br><br>After discussion, consensus from the adolescents that the wording is fine as is.
5	Feeling tired, or having little energy?	No changes.	Feeling tired, or having little energy?	Lagona le lē lava, faapalupē/le lava le malosi?	No changes made.	Added additional piece ("/le lava le malosi") to the professional translation to simplify Samoan language, since adolescents might not know this word.	Answer | n<br>Strongly disagree (total nonsense) | 0<br>Disagree | 0<br>Neutral | 0<br>Agree | 3<br>Strongly agree (totally understandable) | 3	Answer | n<br>Strongly disagree (totally dishonest) | 0<br>Disagree | 0<br>Neutral | 1<br>Agree | 2<br>Strongly agree (totally honest/truthful) | 3	We did not discuss honestly for this specific item in the focus group; rather, we asked the group questions to more generally improve honesty across all items.
6	Feeling bad about yourself — or feeling that you are a failure, or that you have let	Feeling ~~bad~~down about yourself — or feeling that you are a failure, or	Feeling down about yourself — or feeling that you are a failure, or that you have	Faanoanoa ona o oe lava ia — poo lagona o oe o se tagata toi'lalo,	Reworded original prompt to "down" to simplify language and improve understandability.	Some minor modifications to the professional translation, based	Answer | n<br>Strongly disagree (total nonsense) | 0<br>Disagree | 0<br>Neutral | 0<br>Agree | 4	Answer | n<br>Strongly disagree (totally dishonest) | 0<br>Disagree | 1<br>Neutral | 0<br>Agree | 2	We did not discuss honestly for this specific item in the focus group;

	yourself or your family down?	that you have ~~let yourself or your family down~~disappointed yourself or your family?	disappointed yourself or your family?	pe ua e faalumaina oe ma lou aiga?	 Reworded original prompt to “disappointed yourself or your family”.	on the back-translation.	Strongly agree (totally understandable): 2	Strongly agree (totally honest/truthful): 3<br><br>**Why might adolescents not be honest in answering this question?**<br>*“They're going to lie so that they are positive about themselves but in reality they're not.”*<br><br>*“Sometimes they can feel very embarrassed that they are telling people how they are feeling very insecure about themselves.”*	rather, we asked the group questions to more generally improve honesty across all items.
7	Trouble concentrating on things like schoolwork, reading or watching TV?	No changes.	Trouble concentrating on things like schoolwork, reading or watching TV?	Faafaigata ona tuu atoa lou mafaufau i au meaaoga, faitaugatusi poo le matamata o le TV?	No changes made.	No changes made to the professional translation.	Answer: n<br>Strongly disagree (total nonsense): 0<br>Disagree: 0<br>Neutral: 1<br>Agree: 2<br>Strongly agree (totally understandable): 3	Answer: n<br>Strongly disagree (totally dishonest): 0<br>Disagree: 1<br>Neutral: 0<br>Agree: 2<br>Strongly agree (totally honest/truthful): 3<br><br>**Why might adolescents not be honest in answering this question?**<br>*“Sometimes they don't want to say the truth about the things that are going on.”*	We did not discuss honestly for this specific item in the focus group; rather, we asked the group questions to more generally improve honesty across all items.
8	Moving or speaking so slowly that other people could have noticed?<br><br>Or the opposite — being so fidgety or restless that you were moving around a lot more than usual?	No changes.<br><br>Or the opposite — being so fidgety (for example, can't sit still) or restless that you were moving around a lot more than usual?	Moving or speaking so slowly that other people could have noticed?<br><br>Or the opposite — being so fidgety (for example, can't sit still) or restless that you were moving around a lot more than usual?	Gaoioi poo le tautala lemu lava e ono amata ai ona iloa mai e isi?<br><br>Poo le lē mafai ona nofo filemu pe gaoiā ma ua fealuai solo e sili atu nai lo le mea e masani ai?	Group discussed removing “fidgety” since adolescents might not understand this word, but left it in given that it might seem to capture the psychomotor aspect of the symptom. Instead, we decided to added an example to define fidgety “(for example, can't sit still)”.	Made a few minor changes to the professional translation based on the back-translation results.	Answer: n<br>Strongly disagree (total nonsense): 1<br>Disagree: 0<br>Neutral: 0<br>Agree: 3<br>Strongly agree (totally understandable): 2	Answer: n<br>Strongly disagree (totally dishonest): 0<br>Disagree: 1<br>Neutral: 0<br>Agree: 1<br>Strongly agree (totally honest/truthful): 4	Discussed; asked adolescents: “*Any issue in understanding this question?*”<br><br>All adolescents voted to show they are happy with the wording as is.<br><br>We did not discuss honestly for this specific item in the focus group; rather, we asked the group questions to more generally improve honesty across all items.
9	Thoughts that you would be better off dead, or of hurting	Thoughts or feelings that you would be better off dead~~,~~ or ~~of~~ hurting	Thoughts or feelings that you would be better off dead or	Mafaufauga poo faalogona e sili ai le oti poo le faia o nisi tulaga	Added “or feelings” to the original question to make it more easy for adolescents	Modified professional translation because original	Answer: n<br>Strongly disagree (total nonsense): 0<br>Disagree: 0<br>Neutral: 0<br>Agree: 3	Answer: n<br>Strongly disagree (totally dishonest): 0<br>Disagree: 0<br>Neutral: 1<br>Agree: 1	Not discussed in the focus group.

	yourself in some way?	yourself in some way?	hurting yourself in some way?	e faao'o ai le tiga o le tino ia te oe?	to understand; also minor grammar edits.	back-translation of "things to cause you pain" was too vague to match the original construct.	Strongly agree (totally understandable): 3	Strongly agree (totally honest/truthful): 4<br><br>**Why might adolescents not be honest in answering this question?**<br>*"Adolescents have a tendency of trying to hide the pain or hide their feelings"*<br><br>*"They feel as there is nothing they can do or there is nothing that person could do to fix what is wrong with them at all"*	
10	In the ***past year*** have you felt depressed or sad most days, even if you felt okay sometimes?<br><br>Yes/No	No changes.<br><br>No changes.	In the ***past year*** have you felt depressed or sad most days, even if you felt okay sometimes?<br><br>Yes/No	I le ***tausaga ua mavae***, na e lagona ai le faanoanoa loloto poo le faanoanoa i le tele o aso, tusa pe na iai nisi taimi na lelei ai.<br><br>Ioe/Leai	No changes made.	No changes made to the professional translation.	Answer: n<br>Strongly disagree (total nonsense): 0<br>Disagree: 0<br>Neutral: 0<br>Agree: 3<br>Strongly agree (totally understandable): 3	Answer: n<br>Strongly disagree (totally dishonest): 0<br>Disagree: 0<br>Neutral: 0<br>Agree: 3<br>Strongly agree (totally honest/truthful): 3<br><br>**Why might adolescents not be honest in answering this question?**<br>*"They don't want to be bother at times"*<br><br>(this response was from an adolescent who completed the survey twice; this was from their first submission)	Not discussed in the focus group.
11	If you are experiencing any of the problems on this form, how **difficult** have these problems made it for you to do your work, take care of things at home or get along with other people?<br><br>Not difficult at all<br><br>Somewhat difficult<br><br>Very difficult<br><br>Extremely difficult	If you are experiencing any of the problems ~~on this form~~ discussed/listed above, how difficult have these problems made it for you to do your schoolwork, take care of ~~things~~chores at home or get along with other people?<br>No changes.	If you are experiencing any of the problems discussed/listed above, how **difficult** have these problems made it for you to do your schoolwork, take care of chores at home or get along with other people?<br><br>Not difficult at all<br><br>Somewhat difficult<br><br>Very difficult	Afai o e lagonaina se faafitauli o fa'atalanoaina i lenei pepa, o le a se **faigata** na oo i ai i le faiga o au meaaoga i le fale, faatinoga o feau i le fale, poo le galulue faatasi ma isi?<br><br>Leai se faigata<br><br>Faigata laitiiti<br><br>Faigata tele<br><br>Matuā faigata	Simplified question stem language (replaced "on this form" with "discussed/listed above") to help understandability, as the Samoan translation of this makes it too ambiguous otherwise; replaced "work" with "schoolwork" and "things" with "chores" to use more contextually relevant examples from the qualitative interview data and to simplify language to aid comprehension.	Modified the professional translations as "difficult" translated to "very difficult" before (as Likert scales are new concepts in the Samoan language).	Answer: n<br>Strongly disagree (total nonsense): 0<br>Disagree: 0<br>Neutral: 0<br>Agree: 4<br>Strongly agree (totally understandable): 2	Answer: n<br>Strongly disagree (totally dishonest): 0<br>Disagree: 0<br>Neutral: 0<br>Agree: 2<br>Strongly agree (totally honest/truthful): 4	Not discussed in the focus group.

			Extremely difficult						
12	Has there been a time in the past month when you have had serious thoughts about ending your life?<br><br>Yes/No	No changes.<br><br>No changes.	Has there been a time in the past month when you have had serious thoughts about ending your life?<br><br>Yes/No	Na iai se taimi i le masina ua tuanai na e manatu toto'a ai e te pule i lou ola?<br><br>Ioe/Leai	We discussed adding a "decline to answer" option, given how stigmatized this question is in this context, but the expert committee decided not to, as participants have the option to leave their answer blank.	No changes made to the professional translation.	Answer / n<br>Strongly disagree (total nonsense): 0<br>Disagree: 0<br>Neutral: 0<br>Agree: 2<br>Strongly agree (totally understandable): 4	Answer / n<br>Strongly disagree (totally dishonest): 0<br>Disagree: 1<br>Neutral: 1<br>Agree: 1<br>Strongly agree (totally honest/truthful): 3<br><br>Why might adolescents not be honest in answering this question?<br>*"Again they don't want the truth to come out"*<br><br>*"I don't think it's an easy thing to talk about so I believe most teens would just keep it to themselves"*	We did not discuss honestly for this specific item in the focus group; rather, we asked the group questions to more generally improve honesty across all items.
13	Have you ***ever***, in your ***whole life***, tried to kill yourself or made a suicide attempt?<br><br>Yes/No	No changes.<br><br>No changes.	Have you ***ever***, in your ***whole life***, tried to kill yourself or made a suicide attempt?<br><br>Yes/No	Na iai se taimi i lou **olaga atoa**, na e taumafai ai e pule i lou ola?<br><br>Ioe/Leai	No changes made.	Modified professional Samoan translation to make sure concept of making a suicide plan (i.e., concrete thought/plan of how, when, and why – like writing a suicide note) was captured versus only thoughts of suicide; professional translation team modified the wording to reflect this.	Answer / n<br>Strongly disagree (total nonsense): 0<br>Disagree: 0<br>Neutral: 0<br>Agree: 4<br>Strongly agree (totally understandable): 2	Answer / n<br>1 Strongly disagree (totally dishonest): 1<br>Disagree: 0<br>Neutral: 1<br>Agree: 1<br>5 Strongly agree (totally honest/truthful): 3<br><br>Why might adolescents not be honest in answering this question?<br>*"They are scared to admit that they have had a thought"*<br><br>*"It could really go either way that teens might lie or tell the truth"*	We did not discuss honestly for this specific item in the focus group; rather, we asked the group questions to more generally improve honesty across all items.

Table E. Summary of the cross-culturally adapted version of the PHQ-9M for Samoan adolescents, along with summary of changes to the English language wording. When adolescents completed multiple entries for the same answer (for example, if the adolescent completed the survey twice due to technical issues), we reported the Likert Scale answer for their most recent survey attempt but reported the qualitative quotations across all attempts.

	GAD-7 for Samoan Adolescents				Justification and deliberation of English wording changes by the expert committee	Samoan translation and back-translation notes	Adolescent Pretesting		
							Survey (n=6)		Focus group (n=5)
	Original English	Tracked Changes	Final English	Final Samoan			*Is this question easy to understand?*	*Would adolescents answer this question honestly?*	
0	Over the last two weeks, how often have you been bothered by the following problems?<br><br>Not at all<br><br>Several days<br><br>More than half the days<br><br>Nearly every day	No changes.<br><br>No changes.	Over the last two weeks, how often have you been bothered by the following problems?<br><br>Not at all<br><br>Several days<br><br>More than half the days<br><br>Nearly every day	I le lua vaiaso talu ai, e fa'afia ona faapopoleina oe i fa'afitauli o loo sosoo atu?<br><br>Leai ma se taimi<br><br>Mo ni nai aso<br><br>Sili atu i le afa o aso atoa<br><br>Toeitiiti aso uma	No changes made.	Back-translations aligned; no changes made to the professional translation.	Answer: n<br>Strongly disagree (total nonsense): 0<br>Disagree: 0<br>Neutral: 0<br>Agree: 2<br>Strongly agree (totally understandable): 3<br>Did not answer due to technical issues: 1	Answer: n<br>Strongly disagree (totally dishonest): 0<br>Disagree: 0<br>Neutral: 0<br>Agree: 2<br>Strongly agree (totally honest/truthful): 3<br>Did not answer due to technical issues: 1	Discussed question stem only; asked adolescents: "*Any issue in understanding this question?*"<br><br>All adolescents voted to show they are happy with the wording as is.
1	Feeling nervous, anxious or on edge	Feeling nervous, anxious, stressed or on edge	Feeling nervous, anxious, stressed or on edge	Lagona lē to'a, popole; mamafa se mea i le mafaufau/atuatuvale	Themes arose from qualitative interviews that adolescents might not know what the word 'anxiety' means; data suggested that adolescents use the word "stressed" in addition to anxious.	Deliberations about whether to change translation for back-translation that read "something heavy on the mind" but after discussion, decided to leave as is. Otherwise, a few other minor wording changes from the professional translation to better capture concept of "stress".			
2	Not being able to stop or control worrying	No changes.	Not being able to stop or control worrying	Lē mafai ona taofi pe faatonutonu le lagona popole	No changes made.	Back-translations aligned; no changes made to the professional translation.	Answer: n<br>Strongly disagree (total nonsense): 0<br>Disagree: 0<br>Neutral: 0<br>Agree: 1<br>Strongly agree (totally understandable): 4<br>Did not answer due to technical issues: 1	Answer: n<br>Strongly disagree (totally dishonest): 0<br>Disagree: 0<br>Neutral: 0<br>Agree: 3<br>Strongly agree (totally honest/truthful): 2<br>Did not answer due to technical issues: 1	Not discussed in the focus group.
3	Worrying too much about different things	Worrying too much about different things (such as your future, disappointing your family/how other people might judge you, your	Worrying too much about different things (such as your future, disappointing your family/how other people might judge you, your responsibilities at	Soona popole i mea eseese (faapei o lou lumanai, faanoanoa i le faamasino tagata o lou aiga poo isi tagata i au matafaioi i lou aiga,	Samoan member of expert committee recommended that we add in more Samoan-specific examples.	Back-translations aligned; no changes made to the professional translation.	Answer: n<br>Strongly disagree (total nonsense): 0<br>Disagree: 0<br>Neutral: 0<br>Agree: 1<br>Strongly agree (totally understandable): 4<br>Did not answer due to technical issues: 1	Answer: n<br>Strongly disagree (totally dishonest): 0<br>Disagree: 0<br>Neutral: 0<br>Agree: 2<br>Strongly agree (totally honest/truthful): 3<br>Did not answer due to technical issues: 1	Not discussed in the focus group.

<table>
<tr><td></td><td></td><td>responsibilities at home, school, and/or church, etc.)</td><td>home, school, and/or church, etc.)</td><td>aoga, ma/poo le ekalesia, ma isi.)</td><td></td><td></td><td></td><td></td><td></td></tr>
<tr><td>4</td><td>Trouble relaxing</td><td>No changes.</td><td>Trouble relaxing</td><td>Faafaigata ona faato’afilemu</td><td>No changes made.</td><td>Changed Samoan version after professional translation as back-translation read “hard to get a break” but this would imply adolescents would have no opportunity for a break; professional translation team adjusted the language to better reflect this.</td><td><table><tr><th>Answer</th><th>n</th></tr><tr><td>Strongly disagree (total nonsense)</td><td>0</td></tr><tr><td>Disagree</td><td>0</td></tr><tr><td>Neutral</td><td>0</td></tr><tr><td>Agree</td><td>3</td></tr><tr><td>Strongly agree (totally understandable)</td><td>2</td></tr><tr><td>Did not answer due to technical issues</td><td>1</td></tr></table></td><td><table><tr><th>Answer</th><th>n</th></tr><tr><td>Strongly disagree (totally dishonest)</td><td>0</td></tr><tr><td>Disagree</td><td>0</td></tr><tr><td>Neutral</td><td>0</td></tr><tr><td>Agree</td><td>3</td></tr><tr><td>Strongly agree (totally honest/truthful)</td><td>2</td></tr><tr><td>Did not answer due to technical issues</td><td>1</td></tr></table></td><td>Not discussed in the focus group.</td></tr>
<tr><td>5</td><td>Being so restless that it is hard to sit still</td><td>No changes.</td><td>Being so restless that it is hard to sit still</td><td>Ua matua lē to’afimalie ma ua i’u ina faigata ai ona mau pea le nofo</td><td>No changes made.</td><td>No changes to professional translation, though deliberations as back-translation read “uncomfortable” instead of “restless”.</td><td><table><tr><th>Answer</th><th>n</th></tr><tr><td>Strongly disagree (total nonsense)</td><td>0</td></tr><tr><td>Disagree</td><td>0</td></tr><tr><td>Neutral</td><td>0</td></tr><tr><td>Agree</td><td>2</td></tr><tr><td>Strongly agree (totally understandable)</td><td>3</td></tr><tr><td>Did not answer due to technical issues</td><td>1</td></tr></table></td><td><table><tr><th>Answer</th><th>n</th></tr><tr><td>Strongly disagree (totally dishonest)</td><td>0</td></tr><tr><td>Disagree</td><td>0</td></tr><tr><td>Neutral</td><td>1</td></tr><tr><td>Agree</td><td>1</td></tr><tr><td>Strongly agree (totally honest/truthful)</td><td>3</td></tr><tr><td>Did not answer due to technical issues</td><td>1</td></tr></table>
<table><tr><th>Why might adolescents not be honest in answering this question?</th></tr><tr><td>“I think teens would just overlook this question by they way it looks simple”</td></tr></table></td><td>Discussed; asked adolescents: “Any issue in understanding this question?”

All adolescents voted to show they are happy with the wording as is; some adolescents suggested the Samoan is not very understandable, but okay to leave as is since it will be presented with the English as well.</td></tr>
<tr><td>6</td><td>Becoming easily annoyed or irritable</td><td>Becoming easily annoyed, <s>or</s> irritable, or upset</td><td>Becoming easily annoyed, irritable, or upset</td><td>Ua maitaita, itagofie poo le lē fiafia</td><td>Added "upset" since concern that adolescents wouldn’t understand what the word “irritable” means.</td><td>No changes to professional translation.</td><td><table><tr><th>Answer</th><th>n</th></tr><tr><td>Strongly disagree (total nonsense)</td><td>0</td></tr><tr><td>Disagree</td><td>0</td></tr><tr><td>Neutral</td><td>0</td></tr><tr><td>Agree</td><td>1</td></tr><tr><td>Strongly agree (totally understandable)</td><td>4</td></tr><tr><td>Did not answer due to technical issues</td><td>1</td></tr></table></td><td><table><tr><th>Answer</th><th>n</th></tr><tr><td>Strongly disagree (totally dishonest)</td><td>0</td></tr><tr><td>Disagree</td><td>0</td></tr><tr><td>Neutral</td><td>0</td></tr><tr><td>Agree</td><td>1</td></tr><tr><td>Strongly agree (totally honest/truthful)</td><td>4</td></tr><tr><td>Did not answer due to technical issues</td><td>1</td></tr></table></td><td>Not discussed in the focus group.</td></tr>
</table>

								Why might adolescents not be honest in answering this question?<br>*"They don't want to be mean and selfish"*	
7	Feeling afraid as if something awful might happen	Feeling afraid as if something ~~awful~~terrible might happen (for instance, to yourself, your family, or others)	Feeling afraid as if something terrible might happen (for instance, to yourself, your family, or others)	Lagona le fefe i se mea matautia e ono tula'i mai (fa'ataitaiga, ia te oe, lou aiga, po'o nisi tagata)	Modified language to be more clear and simple and also added in locally-relevant examples.	No changes to professional translation.	Answer: n<br>Strongly disagree (total nonsense): 0<br>Disagree: 0<br>Neutral: 0<br>Agree: 1<br>Strongly agree (totally understandable): 4<br>Did not answer due to technical issues: 1	Answer: n<br>Strongly disagree (totally dishonest): 0<br>Disagree: 0<br>Neutral: 0<br>Agree: 2<br>Strongly agree (totally honest/truthful): 3<br>Did not answer due to technical issues: 1<br><br>Why might adolescents not be honest in answering this question?<br>*"Also the lack to confidence to speak up"*	Not discussed in the focus group.
8	If you checked any problems, how difficult have they made it for you to do your work, take care of things at home, or get along with other people?<br><br>Not difficult at all<br>Somewhat difficult<br>Very difficult<br>Extremely difficult	If you checked any problems discussed/listed above, how difficult have they made it for you to do your schoolwork, take care of ~~things~~chores at home, or get along with other people?<br><br>No changes.	If you checked any of the problems discussed/listed above, how difficult have they made it for you to do your schoolwork, take care of chores at home, or get along with other people?<br><br>Not difficult at all<br>Somewhat difficult<br>Very difficult<br>Extremely difficult	Afai o e lagonaina se faafitauli o fa'atalanoaina i lenei pepa, o le a se faigata na oo i ai i le faiga o au meaaoga i le fale, faatinoga o feau i le fale, poo le galulue faatasi ma isi?<br><br>Leai se faigata<br>Faigata laitiiti<br>Faigata tele<br>Matuā faigata	Simplified question stem language (added "discussed/listed above") to help understandability, as the Samoan translation of this makes it too ambiguous otherwise; replaced "work" with "schoolwork" and "things" with "chores" to use more contextually relevant examples from the qualitative interview data and to simplify language to aid comprehension.	Modified the professional translations as "difficult" translated to "very difficult" before (as Likert scales are new concepts in the Samoan language).	Answer: n<br>Strongly disagree (total nonsense): 0<br>Disagree: 0<br>Neutral: 1<br>Agree: 2<br>Strongly agree (totally understandable): 2<br>Did not answer due to technical issues: 1	Answer: n<br>Strongly disagree (totally dishonest): 0<br>Disagree: 0<br>Neutral: 1<br>Agree: 1<br>Strongly agree (totally honest/truthful): 3<br>Did not answer due to technical issues: 1	Not discussed in the focus group.

Table F. Summary of the cross-culturally adapted version of the GAD-7 for Samoan adolescents, along with summary of changes to the English language wording. When adolescents completed multiple entries for the same answer (for example, if the adolescent completed the survey twice due to technical issues), we reported the Likert Scale answer for their most recent survey attempt but reported the qualitative quotations across all attempts.

	Trauma Screen for Samoan Adolescents				Justification and deliberation of English wording changes by the expert committee	Samoan translation and back-translation notes	Adolescent Pretesting		
							Survey (n=6)		
	Original English	Tracked Changes	Final English	Final Samoan			*Is this question easy to understand?*	*Would adolescents answer this question honestly?*	Focus group (n=5)
0	Many children go through frightening or stressful events. Below is a listed of frightening or stressful events that can happen. Mark YES if you have experienced any of these events. Mark NO if you have not experienced these events.<br><br>Yes/No	Many children and adolescents go through frightening or stressful events. Below is a listed of frightening or stressful events that can happen. Mark YES if you have experienced any of these events. Mark NO if you have not experienced these events.<br><br>No changes.	Many children and adolescents go through frightening or stressful events. Below is a list of frightening or stressful events that can happen. Mark YES if you have experienced any of these events. Mark NO if you have not experienced these events.<br><br>Yes/No	O le toatele o tamaiti ma talavou (laiti), e a'afia i ni tulaga e mafua ai ona fefefe ma atuatuvale e o’o i ni tulaga e fefefe ma atuatuvale ai. O loo i lalo atu le lisi o ni mea e tutupu e ono fefefe ma atuatuvale ai. Faailoga le IOE pe afai na e o’o i nisi o nei faafitauli. Faailoga le LEAI pe afai e te le’i o’o i ai.<br><br>Ioe/Leai	We added adolescents to the question stem.	Made a few changes to the professional translations specifically since one component of the back-translation could be interpreted different ways (interpreted as stressful situations or a state of being) so w adjusted the Samoan to be more clear to instead read “affected by situations that can cause fear or anxiety”.	Answer: n<br>Strongly disagree (total nonsense): 0<br>Disagree: 0<br>Neutral: 1<br>Agree: 1<br>Strongly agree (totally understandable): 3<br>Did not answer due to technical issues: 1	Answer: n<br>Strongly disagree (totally dishonest): 0<br>Disagree: 0<br>Neutral: 1<br>Agree: 1<br>Strongly agree (totally honest/truthful): 3<br>Did not answer due to technical issues: 1	Not discussed in the focus group.
1	A severe natural disaster such as a flood, tornado, hurricane, earthquake, or fire	A severe natural disaster such as a tsunami~~flood, tornado,~~ hurricane, ~~earthquake~~, or fire	A severe natural disaster such as a tsunami, hurricane, or fire	O se fa’alavelave fa’alenatura mata’utia e pei o galulolo/sunami, afā, po’o se mū	Modified so examples were more locally-relevant.	No changes made to the professional translation.			
2	Serious accident or injury caused by a car or bike crash, being bitten by a dog, or caused by playing sports	Serious accident or injury caused by a car or bike crash, ~~being bitten~~seriously injured by a dog, or serious injury from~~caused by playing~~ sports	Serious accident or injury caused by a car or bike crash, being seriously injured by a dog, or serious injury from sports	Fa’alavelave mata’utia po’o se manu’a e mafua mai i le lavea i se ta’avale po o se uila vili vae, pe se manu'a tigaina ona o se maile, pe mafua fo’i i ni ta’aloga	Changed to “seriously injured” by a dog, since minor dog bites are common in this setting and not perceived to be traumatic. We also changed the wording to “serious injury” from sports, again to not confuse it with minor injuries in this context.	A few minor changes made to the professional translations based on back-translations.	Answer: n<br>Strongly disagree (total nonsense): 0<br>Disagree: 0<br>Neutral: 1<br>Agree: 1<br>Strongly agree (totally understandable): 3<br>Did not answer due to technical issues: 1	Answer: n<br>Strongly disagree (totally dishonest): 0<br>Disagree: 0<br>Neutral: 1<br>Agree: 2<br>Strongly agree (totally honest/truthful): 2<br>Did not answer due to technical issues: 1	Not discussed in the focus group.
3	Being robbed by threat, force, or weapon	No changes.	Being robbed by threat, force, or weapon	Na faoa ni au mea totino i le tau faamata’u, ave fa’amalosi pe i se auupega malosi	No changes made.	No changes made to the professional translation.	Answer: n<br>Strongly disagree (total nonsense): 0<br>Disagree: 1<br>Neutral: 0<br>Agree: 1<br>Strongly agree (totally understandable): 3<br>Did not answer due to technical issues: 1	Answer: n<br>Strongly disagree (totally dishonest): 0<br>Disagree: 1<br>Neutral: 0<br>Agree: 1<br>Strongly agree (totally honest/truthful): 3<br>Did not answer due to technical issues: 1	Discussed; asked adolescents: “*Any issue in understanding this question?*”

<table>
<tr><td></td><td></td><td></td><td></td><td></td><td></td><td></td><td></td><td></td><td>Two adolescents expressed concerns. One adolescent who has strong Samoan reading skills said the Samoan was very clear, but some adolescents might not understand it. They also said that the word “threat” doesn’t apply – but otherwise recommended to leave the wording as is. No adolescents had any issues with the English and since adolescents didn’t have specific suggestions to change the Samoan, we decided to leave it as is.</td></tr>
<tr><td>4</td><td>Being slapped, punished, or beaten by a relative</td><td>Being <u>severely physically hurt</u><s>slapped,</s><u> or</u> punished, or beaten by a relative</td><td>Being severely physically hurt or punished, or beaten by a relative</td><td>Na matuā afaina, fa’asala, pe na fasi fo’i e se isi o le aiga</td><td>Replaced ‘slapped’ with ‘severely physically hurt’ so that it is not mistaken for normative occurrences/punishment that happens in a usual context (to allow for more variability in responses).</td><td>No changes made to the professional translation.</td><td><table><tr><th>Answer</th><th>n</th></tr><tr><td>Strongly disagree (total nonsense)</td><td>0</td></tr><tr><td>Disagree</td><td>0</td></tr><tr><td>Neutral</td><td>0</td></tr><tr><td>Agree</td><td>2</td></tr><tr><td>Strongly agree (totally understandable)</td><td>3</td></tr><tr><td>Did not answer due to technical issues</td><td>1</td></tr></table></td><td><table><tr><th>Answer</th><th>n</th></tr><tr><td>Strongly disagree (totally dishonest)</td><td>0</td></tr><tr><td>Disagree</td><td>0</td></tr><tr><td>Neutral</td><td>0</td></tr><tr><td>Agree</td><td>2</td></tr><tr><td>Strongly agree (totally honest/truthful)</td><td>3</td></tr><tr><td>Did not answer due to technical issues</td><td>1</td></tr></table></td><td>Discussed in focus group as part of a quality assessment to validate understanding .</td></tr>
<tr><td>5</td><td>Being slapped, knifed, or beaten by a stranger</td><td>Being <u>severely physically hurt</u><s>slapped,</s></td><td>Being severely physically hurt,</td><td>Na matuā afaina, po, afaina i se naifi/polo, pe fasi e se tagata ese</td><td>Replaced ‘slapped’ with ‘severely physically hurt’ so that it is not mistaken for normative</td><td>Minor changes made to the professional translation to use</td><td><table><tr><th>Answer</th><th>n</th></tr><tr><td>Strongly disagree (total nonsense)</td><td>0</td></tr><tr><td>Disagree</td><td>1</td></tr><tr><td>Neutral</td><td>0</td></tr><tr><td>Agree</td><td>1</td></tr></table></td><td><table><tr><th>Answer</th><th>n</th></tr><tr><td>Strongly disagree (totally dishonest)</td><td>0</td></tr><tr><td>Disagree</td><td>0</td></tr><tr><td>Neutral</td><td>1</td></tr><tr><td>Agree</td><td>1</td></tr></table></td><td>Discussed in focus group as part of a quality</td></tr>
</table>

		knifed, or beaten by a stranger	knifed, or beaten by a stranger		occurrences/punishment that happens in a usual context (to allow for more variability in responses).	more simple/informal language for “knife”.	Strongly agree (totally understandable): 3<br>Did not answer due to technical issues: 1	Strongly agree (totally honest/truthful): 3<br>Did not answer due to technical issues: 1	assessment to validate understanding .
6	Seeing a relative get slapped, punished, or beaten	Seeing a relative get severely physically hurt~~slapped~~, punished, or beaten	Seeing a relative get severely physically hurt, punished, or beaten	Vaai i se isi o lona auaiga o matuā afaina, fa’asala pe fasi	Replaced ‘slapped’ with ‘severely physically hurt’ so that is not mistaken for normative occurrences/punishment that happens in a usual context (to allow for more variability in responses).	Modified professional translation so that translation makes clear that the question is asking for physical punishment and not verbal punishment as well.	Answer: n<br>Strongly disagree (total nonsense): 0<br>Disagree: 0<br>Neutral: 0<br>Agree: 0<br>Strongly agree (totally understandable): 5<br>Did not answer due to technical issues: 1	Answer: n<br>Strongly disagree (totally dishonest): 0<br>Disagree: 0<br>Neutral: 0<br>Agree: 1<br>Strongly agree (totally honest/truthful): 4<br>Did not answer due to technical issues: 1<br><br>Why might adolescents not be honest in answering this question?<br>*“Still the chances of them getting something worse or bad back”*	Discussed in focus group as part of a quality assessment to validate understanding .
7	Seeing somebody in your community being slapped, punished, or beaten	Seeing somebody in your community being severely physically hurt~~slapped~~, severely physically punished, or beaten	Seeing somebody in your community being severely hurt, severely physically punished, or beaten	Vaai i se isi o lona nuu o matuā afaina, fa’asala pe fasi	Replaced ‘slapped’ with ‘severely physically hurt’ so that is not mistaken for normative occurrences/punishment that happens in a usual context (to allow for more variability in responses).	No changes made to the professional translation.	Answer: n<br>Strongly disagree (total nonsense): 0<br>Disagree: 0<br>Neutral: 0<br>Agree: 1<br>Strongly agree (totally understandable): 4<br>Did not answer due to technical issues: 1	Answer: n<br>Strongly disagree (totally dishonest): 0<br>Disagree: 0<br>Neutral: 0<br>Agree: 1<br>Strongly agree (totally honest/truthful): 4<br>Did not answer due to technical issues: 1	Not discussed in the focus group.
8	Being touched in your sexual/private parts by an adult/someone older who should not be touching you there	Being touched in your sexual/private parts in a way you didn’t like or that made you feel uncomfortable by an adult/someone older who should not be touching you there	Being touched in your sexual/private parts in a way you didn’t like or that made you feel uncomfortable by an adult/someone older who should not be touching you there	Tagofia e se isi po’o se tagata matua ou itūtino sa e le tatau ona ia tagofia	Updated the language to be more simplified for adolescents.	No changes made to the professional translation.	Answer: n<br>Strongly disagree (total nonsense): 0<br>Disagree: 0<br>Neutral: 1<br>Agree: 2<br>Strongly agree (totally understandable): 2<br>Did not answer due to technical issues: 1	Answer: n<br>Strongly disagree (totally dishonest): 0<br>Disagree: 2<br>Neutral: 1<br>Agree: 0<br>Strongly agree (totally honest/truthful): 2<br>Did not answer due to technical issues: 1<br><br>Why might adolescents not be honest in answering this question?<br>*“Still trying to gain the courage to speak and share there story”*<br><br>*“It can be hard for some to make sure to speak the truth about things”*<br><br>*“I think too many people would feel embarrassed or insecure if they talk about how they were sexually harassed”*	We did not discuss honestly for this specific item in the focus group; rather, we asked the group questions to more generally improve honesty across all items.

9	Being forced/pressured to have sex at a time when you could not say no	No changes.	Being forced/pressured to have sex at a time when you could not say no	Fa’amalosia e faia faigā aiga fa’amalosi i le taimi ua le mafai ona e fai atu i ai e leai	No changes made.	No changes made to the professional translation.	Answer: n<br>Strongly disagree (total nonsense): 0<br>Disagree: 0<br>Neutral: 1<br>Agree: 1<br>Strongly agree (totally understandable): 3<br>Did not answer due to technical issues: 1	Answer: n<br>Strongly disagree (totally dishonest): 0<br>Disagree: 0<br>Neutral: 2<br>Agree: 1<br>Strongly agree (totally honest/truthful): 2<br>Did not answer due to technical issues: 1<br><br>Why might adolescents not be honest in answering this question?<br>*“Again the feeling of being afraid or fearing something will come back for her”*	Not discussed in the focus group.
10	A family member or somebody close dying suddenly or in a violent way	No changes.	A family member or somebody close dying suddenly or in a violent way	Se isi o le auaiga ua oti fa’afuase’i pe i se auala saua	No changes made.	Modified professional translation to replace “faavalemalosi” given that adolescents might not understand this word and that it might come ascross as a “force of nature” which deviates from the construct.<br><br>We also made one minor modification to fix an error in the proofing stage after the focus group.	Answer: n<br>Strongly disagree (total nonsense): 0<br>Disagree: 0<br>Neutral: 0<br>Agree: 1<br>Strongly agree (totally understandable): 4<br>Did not answer due to technical issues: 1	Answer: n<br>Strongly disagree (totally dishonest): 0<br>Disagree: 0<br>Neutral: 0<br>Agree: 1<br>Strongly agree (totally honest/truthful): 4<br>Did not answer due to technical issues: 1	Not discussed in the focus group.
11	Being attacked, shot, stabbed, or seriously injured	No changes.	Being attacked, shot, stabbed, or seriously injured	Osofai’a, fana, tui po’o le manu’a tigāina	No changes made.	No changes made to the professional translation.	Answer: n<br>Strongly disagree (total nonsense): 0<br>Disagree: 1<br>Neutral: 0<br>Agree: 1<br>Strongly agree (totally understandable): 3<br>Did not answer due to technical issues: 1	Answer: n<br>Strongly disagree (totally dishonest): 0<br>Disagree: 0<br>Neutral: 0<br>Agree: 3<br>Strongly agree (totally honest/truthful): 2<br>Did not answer due to technical issues: 1	Discussed; asked adolescents: “*Any issue in understanding this question?*”<br><br>Consensus among adolescents in voting that this item is understandable; no changes recommended.

12	Seeing someone be attacked, shot, stabbed, or seriously injured or killed	No changes.	Seeing someone be attacked, shot, stabbed, or seriously injured or killed	Vaai i se isi o osofai'a, fana, tui pe manu'a tigaina pe ua fasiotia	No changes made.	No changes made to the professional translation.	Answer: n<br>Strongly disagree (total nonsense): 0<br>Disagree: 0<br>Neutral: 0<br>Agree: 1<br>Strongly agree (totally understandable): 4<br>Did not answer due to technical issues: 1	Answer: n<br>Strongly disagree (totally dishonest): 0<br>Disagree: 0<br>Neutral: 0<br>Agree: 2<br>Strongly agree (totally honest/truthful): 3<br>Did not answer due to technical issues: 1	Not discussed in the focus group.
13	Having a stressful or frightening medical procedure	Having a stressful or frightening medical procedure (e.g., heart or brain surgery)	Having a stressful or frightening medical procedure (e.g., heart or brain surgery)	Fefe po'o le atuatuvale i fa'agasologa o talavai a le falema'i (fa'ataitaiga taotoga o le fatu poo le fai'ai)	Added example because the original back-translation did not capture the concept well enough – and then selected these two examples because they seemed more life-threatening.	No changes made to the professional translation.	Answer: n<br>Strongly disagree (total nonsense): 0<br>Disagree: 0<br>Neutral: 0<br>Agree: 2<br>Strongly agree (totally understandable): 3<br>Did not answer due to technical issues: 1	Answer: n<br>Strongly disagree (totally dishonest): 0<br>Disagree: 0<br>Neutral: 0<br>Agree: 1<br>Strongly agree (totally honest/truthful): 4<br>Did not answer due to technical issues: 1	Not discussed in the focus group.
14	Being around a war	Being around a war, meaning a state of armed conflict that can result in death (which is not the same as inter-village conflicts)	Being around a war, meaning a state of armed conflict that can result in death (which is not the same as inter-village conflicts)	Sa e auai i se taua e pei o tulaga o feteenaiga faaauupegaina e mafai ona i'u ai i le oti (e ese mai i feeseeseaiga i totonu o nuu)	We added a few words to define "war", as some adolescents in the focus groups were not sure if inter-village conflicts (for example, challenges that come up between families regarding land or titles, but doesn't necessarily result in death) would also qualify as a "war".	No changes made to the professional translation.	Answer: n<br>Strongly disagree (total nonsense): 1<br>Disagree: 0<br>Neutral: 0<br>Agree: 2<br>Strongly agree (totally understandable): 2<br>Did not answer due to technical issues: 1	Answer: n<br>Strongly disagree (totally dishonest): 0<br>Disagree: 0<br>Neutral: 1<br>Agree: 2<br>Strongly agree (totally honest/truthful): 2<br>Did not answer due to technical issues: 1	Discussed; asked adolescents: "*Any issue in understanding this question?*"<br><br>There was lack of clarity in how war was defined (for example, would battles between villages qualify as there is no local context for this). Based on this, we modified the wording of the item.
15	Any other stressful or frightening event<br><br>Describe:	Any other stressful or frightening event that has not been included above<br><br>Describe:	Any other stressful or frightening event that has not been included above<br><br>Describe:	So'o se isi mea na tupu e ono mafua ai le atuatuvale ma le fefe e le'o aofia i luga<br><br>Faamatala:	Modified for clarity.	No changes made to the professional translation.	Answer: N<br>Strongly disagree (total nonsense): 0<br>Disagree: 0<br>Neutral: 0<br>Agree: 3<br>Strongly agree (totally understandable): 2<br>Did not answer due to technical issues: 1	Answer: n<br>Strongly disagree (totally dishonest): 0<br>Disagree: 0<br>Neutral: 0<br>Agree: 3<br>Strongly agree (totally honest/truthful): 2<br>Did not answer due to technical issues: 1	Not discussed in focus groups.
16	Which of these events bothers you most?	Which of these events listed above bothers you most?	Which of these events listed above bothers you most?	le fea o vaega nei o lisi atu i luga e pito sili ona fa'alavelave ia te oe?	Modified for clarity.	No changes made to the professional translation.	Answer: n<br>Strongly disagree (total nonsense): 0<br>Disagree: 0<br>Neutral: 1<br>Agree: 2	Answer: n<br>Strongly disagree (totally dishonest): 0<br>Disagree: 0<br>Neutral: 1<br>Agree: 2	Not discussed in the focus group.

							Strongly agree (totally understandable): 2<br>Did not answer due to technical issues: 1	Strongly agree (totally honest/truthful): 2<br>Did not answer due to technical issues: 1	
17	If you answered NO to all of the above questions, STOP. If you answered YES to any of the above questions, please answer the following questions.	No changes.	If you answered NO to all of the above questions, STOP. If you answered YES to any of the above questions, please answer the following questions.	Afai na e tali LEAI i fesili uma o lo'o i luga, TAOFI. Afai na e tali IOE i so'o se fesili lava o lo'o i luga, fa'amolemole tali fesili o lo'o mulimuli mai.	Modified for clarity.	No changes made to the professional translation.	Answer: n<br>Strongly disagree (total nonsense): 0<br>Disagree: 0<br>Neutral: 0<br>Agree: 2<br>Strongly agree (totally understandable): 3<br>Did not answer due to technical issues: 1	Answer: n<br>Strongly disagree (totally dishonest): 0<br>Disagree: 0<br>Neutral: 0<br>Agree: 2<br>Strongly agree (totally honest/truthful): 3<br>Did not answer due to technical issues: 1	Not discussed in the focus group.
18	When the event happened, did you feel:<br><br>Yes/No	No changes.	When the event happened, did you feel:<br><br>Yes/No	I le taimi na tupu ai le fa'alavelave, na e lagonaina:<br><br>Ioe/Leai	No changes made.	No changes made to the professional translation.			
19	Fear that you were going to die or be seriously injured?	No changes.	Fear that you were going to die or be seriously injured?	Fefe ona o le a e oti pe manu'a tigaina?	No changes made.	No changes made to the professional translation.			
20	Fear that someone else was seriously hurt?	No changes.	Fear that someone else was seriously hurt?	Fefe e iai se isi na manu'a tigaina?	No changes made.	No changes made to the professional translation.	Answer: n<br>Strongly disagree (total nonsense): 0<br>Disagree: 0<br>Neutral: 0<br>Agree: 1<br>Strongly agree (totally understandable): 4<br>Did not answer due to technical issues: 1	Answer: n<br>Strongly disagree (totally dishonest): 0<br>Disagree: 0<br>Neutral: 0<br>Agree: 2<br>Strongly agree (totally honest/truthful): 3<br>Did not answer due to technical issues: 1	Not discussed in the focus group.
21	Unable to help yourself?	No changes.	Unable to help yourself?	Le mafai ona e fesoasoani ia te oe lava?	No changes made.	No changes made to the professional translation.	Answer: n<br>Strongly disagree (total nonsense): 0<br>Disagree: 0<br>Neutral: 0<br>Agree: 2<br>Strongly agree (totally understandable): 3<br>Did not answer due to technical issues: 1	Answer: n<br>Strongly disagree (totally dishonest): 0<br>Disagree: 0<br>Neutral: 0<br>Agree: 2<br>Strongly agree (totally honest/truthful): 3<br>Did not answer due to technical issues: 1	Not discussed in the focus group.
22	Shame or disgust?	No changes.	Shame or disgust?	Māasiasi po'o le inoino?	No changes made.	No changes made to the professional translation.	Answer: n<br>Strongly disagree (total nonsense): 0<br>Disagree: 0<br>Neutral: 0<br>Agree: 2<br>Strongly agree (totally understandable): 3<br>Did not answer due to technical issues: 1	Answer: n<br>Strongly disagree (totally dishonest): 0<br>Disagree: 0<br>Neutral: 0<br>Agree: 2<br>Strongly agree (totally honest/truthful): 3<br>Did not answer due to technical issues: 1	Not discussed in the focus group.

Table G. Summary of the cross-culturally adapted version of the Trauma Screener for Samoan adolescents, along with summary of changes to the English language wording. When adolescents completed multiple entries for the same answer (for example, if the adolescent completed the survey twice due to technical issues), we reported the Likert Scale answer for their most recent survey attempt but reported the qualitative quotations across all attempts.

	CPSS-V for Samoan Adolescents				Justification and deliberation of English wording changes by the expert committee	Samoan translation and back-translation notes	Adolescent Pretesting		
							Survey (n=6)		
	Original English	Tracked Changes	Final English	Final Samoan			*Is this question easy to understand?*	*Would adolescents answer this question honestly?*	Focus group (n=5)
0	Sometimes scary or upsetting things happen to kids. It might be something like a car accident, getting beaten up, living through an earthquake, being robbed, being touched in a way you didn't like, having a parent get hurt or killed, or some other very upsetting event.	Sometimes scary or upsetting things happen in your life~~to kids~~. It might be something like ~~a car accident,~~ getting beaten up, living through ~~an earthquake~~tsunami, ~~being robbed~~ witnessing violence at home, being touched in a way you didn't like or that made you feel uncomfortable, having a parent get hurt or killed, or some other very upsetting event.	Sometimes scary or upsetting things happen in your life. It might be something like getting beaten up, living through a tsunami, witnessing violence at home, being touched in a way you didn't like or that made you feel uncomfortable, having a parent get hurt or killed, or some other very upsetting event.	E iai taimi e tutupu ai mea e fefe ai pe e te faanoanoa ai i lou olaga. E pei o le fasi o oe, sao mai se galulolo sa tupu, molimauina o sauaga i totonu o le aiga, tagofia o oe i se auala na e le mana'o ai ma e le filemu ai, fa'amanu'alia o se matua pe ua maliu foi, poo se isi lava fa'alavelave matuia.	Updated the examples to be more locally relevant (including most commonly described traumatic events in the adult qualitative interviews (1)). The language was also simplified.	Several modifications based on back-translation. For example, the back-translation was "distractions" for "upsetting things", and "sickness" for "get hurt"; both of which deviated too far from the original construct. The Samoan wording was also simplified for an adolescent reader.	Answer: n<br>Strongly disagree (total nonsense): 0<br>Disagree: 0<br>Neutral: 1<br>Agree: 2<br>Strongly agree (totally understandable): 2<br>Did not answer due to technical issues: 1	Answer: n<br>Strongly disagree (totally dishonest): 0<br>Disagree: 0<br>Neutral: 2<br>Agree: 1<br>Strongly agree (totally honest/truthful): 2<br>Did not answer due to technical issues: 1<br><br>Why might adolescents not be honest in answering this question?<br>*"Too scared to admit the truth"*	Not discussed in the focus group.
1	Please write down the scary or upsetting thing that bothers you the most when you think about it (this should be the event you listed in the Trauma Screen, if the Trauma Screen was used):	Please write down the scary or upsetting thing that bothers you the most when you think about it or the thing that you try not to think about (this should be the event you listed in the Trauma Screen~~, if the Trauma Screen was used~~):	Please write down the scary or upsetting thing that bothers you the most when you think about it or the thing that you try not to think about (this should be the event you listed in the Trauma Screen):	Fa'amolemole tusi i lalo le fa'afitauli na pito sili ona e fefe ai, pe sa tele ina fa'afatu'ulu (e.g., fa'alavelave) ia te oe, pe a e mafaufau iai, poo se mea oloo e taumafai e aua e te mafaufau iai (o le fa'alavelave lea na 'e lisia i le Iloiloga o ni a'afiaga talu ai se fa'afitauli matuia, pe afai na faaaoga Trauma Screen):	Added content to simplify language.	Several changes to the professional translation to simplify the language for adolescents.			
2	When did it happen?<br>________________	No changes.	When did it happen?<br>________________	O anafea na tupu ai?<br>________________	No changes made.	No changes made to the professional translation.			
3	These questions ask about how you feel about the upsetting thing you wrote down. Read each question carefully.	No changes.	These questions ask about how you feel about the upsetting thing you wrote down. Read each question carefully.	O fesili nei o lo'o fesiligia ai ou fa'alogona ina ua e tusia le mea sa e faanoanoa ai. Faitau lelei fesili taitasi. Li'o le	No changes made.	Change to professional translation as concept for "upsetting" was	Answer: n<br>Strongly disagree (total nonsense): 0<br>Disagree: 0<br>Neutral: 1<br>Agree: 2	Answer: n<br>Strongly disagree (totally dishonest): 0<br>Disagree: 0<br>Neutral: 1<br>Agree: 2	Not discussed in the focus group.

<table>
<tr><th></th><th></th><th></th><th></th><th></th><th></th><th></th><th></th><th></th><th></th></tr>
<tr>
<td></td>
<td>Then circle the number (0-4) that best describes how often that problem has bothered you IN THE LAST MONTH.

Not at all

Once a week or less/ a little

2 to 3 times a week/ somewhat

4 to 5 times a week/ a lot

6 or more times a week/almost always</td>
<td>No changes.</td>
<td>Then circle the number (0-4) that best describes how often that problem has bothered you IN THE LAST MONTH.

Not at all

Once a week or less/ a little

2 to 3 times a week/ somewhat

4 to 5 times a week/ a lot

6 or more times a week/almost always</td>
<td>fuainumera (0-4) e te iloa o lo’o faamatala lelei mai ai pe na faafia ona faafatu’ulu ia te oe lena faalavelave na tupu I LE MASINA UA TE’A.

E leai lava

E faatasi i le vaiaso pe le tele fo’i

E faalua pe faatolu i le vaiaso/e feoloolo lava

E faafa pe faalima i le vaiaso/e tele taimi

E faaono pe sili atu i le vaiaso/toe lava o taimi uma</td>
<td></td>
<td>back-translated to “sad”.</td>
<td><table><tr><td>Strongly agree (totally understandable)</td><td>2</td></tr><tr><td>Did not answer due to technical issues</td><td>1</td></tr></table></td>
<td><table><tr><td>Strongly agree (totally honest/truthful)</td><td>2</td></tr><tr><td>Did not answer due to technical issues</td><td>1</td></tr></table></td>
<td></td>
</tr>
<tr>
<td>4</td>
<td>Having upsetting thoughts or pictures about it that came into your head when you didn’t want them to</td>
<td>Having upsetting* thoughts or pictures about it that came into your head when you didn’t want them to

*Upsetting means that they made you feel unhappy or worried</td>
<td>Having upsetting* thoughts or pictures about it that came into your head when you didn’t want them to

*Upsetting means that they made you feel unhappy or worried</td>
<td>Iai fa’alogona le fiafia* po’o ni ata fa’atatau i ia fa’alogona e o’o mai i lou mafaufau ae e te le’i mana’o ai

*O faalogona le fiafia ua faauigaina na o’o ai ina e fa'anoanoa pe popole</td>
<td>Added a definition of upsetting to simplify language.</td>
<td>Modified slightly based on back-translations, as concept of ‘upsetting’ back-translated to ‘unhappiness’ and ‘disappointment’.</td>
<td></td>
<td></td>
<td></td>
</tr>
<tr>
<td>5</td>
<td>Having bad dreams or nightmares</td>
<td>No changes.</td>
<td>Having bad dreams or nightmares</td>
<td>Faia ni miti lē lelei poo miti taufaafefe</td>
<td>No changes made.</td>
<td>No changes made to the professional translation.</td>
<td><table><tr><th>Answer</th><th>n</th></tr><tr><td>Strongly disagree (total nonsense)</td><td>0</td></tr><tr><td>Disagree</td><td>0</td></tr><tr><td>Neutral</td><td>0</td></tr><tr><td>Agree</td><td>2</td></tr><tr><td>Strongly agree (totally understandable)</td><td>3</td></tr><tr><td>Did not answer due to technical issues</td><td>1</td></tr></table></td>
<td><table><tr><th>Answer</th><th>n</th></tr><tr><td>Strongly disagree (totally dishonest)</td><td>0</td></tr><tr><td>Disagree</td><td>0</td></tr><tr><td>Neutral</td><td>0</td></tr><tr><td>Agree</td><td>2</td></tr><tr><td>Strongly agree (totally honest/truthful)</td><td>2</td></tr><tr><td>Did not answer due to technical issues</td><td>1</td></tr><tr><td>Did not answer; reason unknown</td><td>1</td></tr></table></td>
<td>Not discussed in the focus group.</td>
</tr>
<tr>
<td>6</td>
<td>Acting or feeling as if it was happening again (seeing or hearing something and feeling as if you are there again)</td>
<td>No changes.</td>
<td>Acting or feeling as if it was happening again (seeing or hearing something and feeling as if you are there again)</td>
<td>Fa’atinoga po’o ni fa’alogona pei ua toe tupu fo’i (vaaia pe lagona se mea ma fa’alogona pei ua toe tupu fo’i)</td>
<td>No changes made.</td>
<td>No changes made to the professional translation.</td>
<td><table><tr><th>Answer</th><th>n</th></tr><tr><td>Strongly disagree (total nonsense)</td><td>0</td></tr><tr><td>Disagree</td><td>0</td></tr><tr><td>Neutral</td><td>0</td></tr><tr><td>Agree</td><td>2</td></tr><tr><td>Strongly agree (totally understandable)</td><td>3</td></tr></table></td>
<td><table><tr><th>Answer</th><th>n</th></tr><tr><td>Strongly disagree (totally dishonest)</td><td>0</td></tr><tr><td>Disagree</td><td>0</td></tr><tr><td>Neutral</td><td>0</td></tr><tr><td>Agree</td><td>2</td></tr><tr><td>Strongly agree (totally honest/truthful)</td><td>3</td></tr></table></td>
<td>Not discussed in the focus group.</td>
</tr>
</table>

							Did not answer due to technical issues: 1	Did not answer due to technical issues: 1	
7	Feeling upset when you remember what happened (for example, feeling scared, angry, sad, guilty, confused)	No changes.	Feeling upset when you remember what happened (for example, feeling scared, angry, sad, guilty, confused)	Fa'alogona lē fiafia pe a e toe manatua se mea na tupu (fa'ata'ita'iga, fa'alogona fefe, ita, fa'anoanoa, ta'usala, lē mautonu)	No changes made.	No changes made to the professional translation.	Answer: n<br>Strongly disagree (total nonsense): 0<br>Disagree: 0<br>Neutral: 0<br>Agree: 3<br>Strongly agree (totally understandable): 2<br>Did not answer due to technical issues: 1	Answer: n<br>Strongly disagree (totally dishonest): 0<br>Disagree: 0<br>Neutral: 0<br>Agree: 3<br>Strongly agree (totally honest/truthful): 2<br>Did not answer due to technical issues: 1	Not discussed in focus groups.
8	Having feelings in your body when you remember what happened (for example, sweating, heart beating fast, stomach or head hurting)	No changes.	Having feelings in your body when you remember what happened (for example, sweating, heart beating fast, stomach or head hurting)	Iai fa'alogona o lou tino pe a toe manatua se mea na tupu (fa'ata'ita'iga, afu, vave le tātā o le fatu, tigā le manava po'o le ulu)	No changes made.	No changes made to the professional translation.	Answer: n<br>Strongly disagree (total nonsense): 0<br>Disagree: 0<br>Neutral: 1<br>Agree: 2<br>Strongly agree (totally understandable): 2<br>Did not answer due to technical issues: 1	Answer: n<br>Strongly disagree (totally dishonest): 0<br>Disagree: 0<br>Neutral: 1<br>Agree: 2<br>Strongly agree (totally honest/truthful): 2<br>Did not answer due to technical issues: 1	Not discussed in the focus group.
9	Trying not to think about it or have feelings about it	No changes.	Trying not to think about it or have feelings about it	O loo taumafai e aua le mafaufau i ai, pe iai ni lagona e faatatau i ai	No changes made.	No changes made to the professional translation.	Answer: n<br>Strongly disagree (total nonsense): 0<br>Disagree: 0<br>Neutral: 0<br>Agree: 2<br>Strongly agree (totally understandable): 3<br>Did not answer due to technical issues: 1	Answer: n<br>Strongly disagree (totally dishonest): 0<br>Disagree: 0<br>Neutral: 0<br>Agree: 2<br>Strongly agree (totally honest/truthful): 3<br>Did not answer due to technical issues: 1	Not discussed in the focus group.
10	Trying to stay away from anything that reminds you of what happened (for example, people, places, or conversations about it)	No changes.	Trying to stay away from anything that reminds you of what happened (for example, people, places, or conversations about it)	Taumafai e 'alo ese mai so'o se mea e toe fa'amanatu atu ai ia te oe le mea na tupu (fa'ata'ita'iga, tagata, nofoaga, po'o se talanoaga fa'atatau iai)	No changes made.	No changes made to the professional translation.	Answer: n<br>Strongly disagree (total nonsense): 0<br>Disagree: 0<br>Neutral: 0<br>Agree: 3<br>Strongly agree (totally understandable): 2<br>Did not answer due to technical issues: 1	Answer: n<br>Strongly disagree (totally dishonest): 0<br>Disagree: 0<br>Neutral: 0<br>Agree: 3<br>Strongly agree (totally honest/truthful): 2<br>Did not answer due to technical issues: 1	Not discussed in the focus group.
11	Not being able to remember an important part of what happened	No changes.	Not being able to remember an important part of what happened	Lē mafai ona toe manatua se vaega taua o le mea na tupu	No changes made.	No changes made to the professional translation.	Answer: n<br>Strongly disagree (total nonsense): 0<br>Disagree: 0<br>Neutral: 0<br>Agree: 3<br>Strongly agree (totally understandable): 2<br>Did not answer due to technical issues: 1	Answer: n<br>Strongly disagree (totally dishonest): 0<br>Disagree: 0<br>Neutral: 0<br>Agree: 3<br>Strongly agree (totally honest/truthful): 2<br>Did not answer due to technical issues: 1	Not discussed in the focus group.
12	Having bad thoughts about yourself, other people, or the world (for example, "I can't do anything right", "All people are bad",	No changes.	Having bad thoughts about yourself, other people, or the world (for example, "I can't do anything right", "All people are bad",	Fa'alogona leaga fa'atatau ia te oe lava, isi tagata, po'o le lalolagi (fa'ata'ita'iga, "E leai se mea sa'o ou te faia", "O tagata uma e	No changes made.	No changes made to the professional translation.	Answer: n<br>Strongly disagree (total nonsense): 0<br>Disagree: 0<br>Neutral: 0<br>Agree: 2<br>Strongly agree (totally understandable): 3	Answer: n<br>Strongly disagree (totally dishonest): 0<br>Disagree: 0<br>Neutral: 0<br>Agree: 2<br>Strongly agree (totally honest/truthful): 3	Not discussed in the focus group.

	"The world is a scary place")		"The world is a scary place")	leaga", "O le lalolagi o se nofoaga taufa'afefe")			Did not answer due to technical issues: 1	Did not answer due to technical issues: 1	
13	Thinking that what happened is your fault (for example, "I should have known better", "I shouldn't have done that", "I deserved it")	No changes.	Thinking that what happened is your fault (for example, "I should have known better", "I shouldn't have done that", "I deserved it")	Mafaufauga o oe e mafua ai le mea na tupu (fa'ata'ita'iga, "Sa tatau ona ou iloa lelei", "Sa lē tatau ona ou faia ", "O le mea lena ou te maua")	No changes made.	A few modifications to the professional translation, as back-translation of quotes didn't match the original construct.	Answer: n<br>Strongly disagree (total nonsense): 0<br>Disagree: 0<br>Neutral: 0<br>Agree: 1<br>Strongly agree (totally understandable): 4<br>Did not answer due to technical issues: 1	Answer: n<br>Strongly disagree (totally dishonest): 0<br>Disagree: 0<br>Neutral: 0<br>Agree: 1<br>Strongly agree (totally honest/truthful): 4<br>Did not answer due to technical issues: 1	Not discussed in the focus group.
14	Having strong bad feelings (like fear, anger, guilt, or shame)	No changes.	Having strong bad feelings (like fear, anger, guilt, or shame)	Malosi ni lagona lē lelei (pei o le fefe, ita, lagona le sesē, po'o le maasiasi)	No changes made.	No changes made to the professional translation.	Answer: n<br>Strongly disagree (total nonsense): 0<br>Disagree: 0<br>Neutral: 0<br>Agree: 2<br>Strongly agree (totally understandable): 3<br>Did not answer due to technical issues: 1	Answer: n<br>Strongly disagree (totally dishonest): 0<br>Disagree: 0<br>Neutral: 0<br>Agree: 1<br>Strongly agree (totally honest/truthful): 4<br>Did not answer due to technical issues: 1	Not discussed in the focus group.
15	Having much less interest in doing things you used to do	Having much less interest in doing things you used to do (for example, spending time with friends, playing games, etc.)	Having much less interest in doing things you used to do (for example, spending time with friends, playing games, etc.)	Ua itiiti atu le fiafia e fa'atino ni galuega sa masani ai (fa'ata'itaiga: evaga ma uo, ta'aloga)	Added in some examples since original back-translation wasn't specific enough to match the construct.	No changes made to the professional translation.	Answer: n<br>Strongly disagree (total nonsense): 0<br>Disagree: 0<br>Neutral: 0<br>Agree: 3<br>Strongly agree (totally understandable): 2<br>Did not answer due to technical issues: 1	Answer: n<br>Strongly disagree (totally dishonest): 0<br>Disagree: 0<br>Neutral: 0<br>Agree: 3<br>Strongly agree (totally honest/truthful): 2<br>Did not answer due to technical issues: 1	Not discussed in the focus group.
16	Not feeling close to your friends or family or not wanting to be around them	No changes.	Not feeling close to your friends or family or not wanting to be around them	Lē lagonaina le vavalalata i uo ma aiga pe le fiafia fo'i e fa'atasi ma i latou	No changes made.	No changes made to the professional translation.	Answer: n<br>Strongly disagree (total nonsense): 0<br>Disagree: 0<br>Neutral: 0<br>Agree: 3<br>Strongly agree (totally understandable): 2<br>Did not answer due to technical issues: 1	Answer: n<br>Strongly disagree (totally dishonest): 0<br>Disagree: 1<br>Neutral: 1<br>Agree: 1<br>Strongly agree (totally honest/truthful): 2<br>Did not answer due to technical issues: 1<br><br>Why might adolescents not be honest in answering this question?<br>*"I feel like most teens would lie so that others would think that they have a healthy relationship with their families"*	Discussed, as preliminary testing with adult colleagues questioned understandability; some discussion to change the Samoan translation, but decided to keep the existing translation the same because it will be presented with the English version side-by-side.<br><br>There was consensus

									that adolescents would answer this question honestly.
17	Trouble having good feelings (like happiness or love) or trouble having any feelings at all	No changes.	Trouble having good feelings (like happiness or love) or trouble having any feelings at all	Faigatā ona maua ni lagona lelei (pei o le fiafia po’o le alofa) pe faigatā ona maua ni lagona	No changes made.	No changes made to the professional translation.	Answer – n<br>Strongly disagree (total nonsense) – 0<br>Disagree – 0<br>Neutral – 0<br>Agree – 3<br>Strongly agree (totally understandable) – 2<br>Did not answer due to technical issues – 1	Answer – n<br>Strongly disagree (totally dishonest) – 0<br>Disagree – 0<br>Neutral – 0<br>Agree – 3<br>Strongly agree (totally honest/truthful) – 2<br>Did not answer due to technical issues – 1	Not discussed in the focus group.
18	Getting angry easily (for example, yelling, hitting others, throwing things)	No changes.	Getting angry easily (for example, yelling, hitting others, throwing things)	Maitaita gofie (fa’ata’ita’iga, e’ē, pa’ilima i isi, taua’i solo ni mea)	No changes made.	No changes made to the professional translation.	Answer – n<br>Strongly disagree (total nonsense) – 0<br>Disagree – 0<br>Neutral – 0<br>Agree – 2<br>Strongly agree (totally understandable) – 3<br>Did not answer due to technical issues – 1	Answer – n<br>Strongly disagree (totally dishonest) – 0<br>Disagree – 0<br>Neutral – 0<br>Agree – 2<br>Strongly agree (totally honest/truthful) – 3<br>Did not answer due to technical issues – 1	Not discussed in the focus group.
19	Doing things that might hurt yourself (for example, taking drugs, drinking alcohol, running away, cutting yourself)	No changes.	Doing things that might hurt yourself (for example, taking drugs, drinking alcohol, running away, cutting yourself)	Faia o ni mea e ono a’afia ai oe (fa’ata’ita’iga, tagofia o fuala’au fa’asaina, inu ava malosi, sola ese, tasele pe fa’amanu’aina oe lava)	No changes made.	No changes made to the professional translation.	Answer – n<br>Strongly disagree (total nonsense) – 0<br>Disagree – 0<br>Neutral – 0<br>Agree – 3<br>Strongly agree (totally understandable) – 2<br>Did not answer due to technical issues – 1	Answer – n<br>Strongly disagree (totally dishonest) – 0<br>Disagree – 0<br>Neutral – 1<br>Agree – 2<br>Strongly agree (totally honest/truthful) – 2<br>Did not answer due to technical issues – 1	Not discussed in the focus group.
20	Being very careful or on the lookout for danger (for example, checking to see who is around you and what is around you)	No changes.	Being very careful or on the lookout for danger (for example, checking to see who is around you and what is around you)	Fa’aete’ete tele po’o le va’ava’ai toto’a pe iai se tulaga faigata poo se faalavelave (e ono tupu) (fa’ata’ita’iga, siakiina po’o ai o i ou autafa, ae po’o a ni mea o siomia ai oe)	No changes made.	No changes made to the professional translation.	Answer – n<br>Strongly disagree (total nonsense) – 0<br>Disagree – 0<br>Neutral – 1<br>Agree – 1<br>Strongly agree (totally understandable) – 3<br>Did not answer due to technical issues – 1	Answer – n<br>Strongly disagree (totally dishonest) – 0<br>Disagree – 0<br>Neutral – 1<br>Agree – 1<br>Strongly agree (totally honest/truthful) – 3<br>Did not answer due to technical issues – 1	Not discussed in the focus group.
21	Being jumpy or easily scared (for example, when someone walks up behind you, when you hear a loud noise)	No changes.	Being jumpy or easily scared (for example, when someone walks up behind you, when you hear a loud noise)	Mate’ite’i pe fefe gofie (fa’ata’ita’iga, pe a savali atu i ou tua se tagata, pe a lagonaina se pa’ō leotele)	No changes made.	No changes made to the professional translation.	Answer – n<br>Strongly disagree (total nonsense) – 0<br>Disagree – 1<br>Neutral – 0<br>Agree – 2<br>Strongly agree (totally understandable) – 2<br>Did not answer due to technical issues – 1	Answer – n<br>Strongly disagree (totally dishonest) – 0<br>Disagree – 0<br>Neutral – 1<br>Agree – 2<br>Strongly agree (totally honest/truthful) – 2<br>Did not answer due to technical issues – 1	Discussed; asked adolescents: “*Any issue in understanding this question?*”<br><br>Consensus that the English and Samoan was clear. No changes need to be made.

22	Having trouble paying attention (for example, losing track of a story on TV, forgetting what you read, unable to pay attention in class)	No changes.	Having trouble paying attention (for example, losing track of a story on TV, forgetting what you read, unable to pay attention in class)	Faafaigata ona ua'i le mafaufau (fa'ata'ita'iga, lē mulimuli lelei i se tala [o matamata ai] i le TV, galo se mea na e faitau iai, le mafai ona ua'i le fa'alogo i le aoga)	No changes made.	No changes made to the professional translation.	Answer: n<br>Strongly disagree (total nonsense): 0<br>Disagree: 0<br>Neutral: 0<br>Agree: 1<br>Strongly agree (totally understandable): 4<br>Did not answer due to technical issues: 1	Answer: n<br>Strongly disagree (totally dishonest): 0<br>Disagree: 0<br>Neutral: 0<br>Agree: 1<br>Strongly agree (totally honest/truthful): 4<br>Did not answer due to technical issues: 1	Not discussed in the focus group.
23	Having trouble falling or staying asleep	No changes.	Having trouble falling or staying asleep	Faigatā ona moe pe faaauau le moe	No changes made.	No changes made to the professional translation.	Answer: n<br>Strongly disagree (total nonsense): 0<br>Disagree: 0<br>Neutral: 0<br>Agree: 1<br>Strongly agree (totally understandable): 4<br>Did not answer due to technical issues: 1	Answer: n<br>Strongly disagree (totally dishonest): 0<br>Disagree: 0<br>Neutral: 0<br>Agree: 2<br>Strongly agree (totally honest/truthful): 3<br>Did not answer due to technical issues: 1	Not discussed in the focus group.
24	Have the problems above been getting in the way of these parts of your life IN THE PAST MONTH?<br><br>Yes/No	No changes.<br><br>No changes.	Have the problems above been getting in the way of these parts of your life IN THE PAST MONTH?<br><br>Yes/No	Faamata na avea faafitauli (o ta'ua) i luga atu ma faalavelave i vaega nei o lou olaga i le MASINA UA TUANA'I?<br><br>Ioe/Leai	No changes made.	No changes made to the professional translation.	Answer: n<br>Strongly disagree (total nonsense): 0<br>Disagree: 0<br>Neutral: 1<br>Agree: 2<br>Strongly agree (totally understandable): 2<br>Did not answer due to technical issues: 1	Answer: n<br>Strongly disagree (totally dishonest): 0<br>Disagree: 0<br>Neutral: 1<br>Agree: 2<br>Strongly agree (totally honest/truthful): 2<br>Did not answer due to technical issues: 1	Not discussed in the focus group.
25	Fun things you want to do	No changes.	Fun things you want to do	O mea faafiafia (loto) e te mana'o e fai	No changes made.	No changes made to the professional translation.			
26	Doing your chores	No changes.	Doing your chores	Faiga o au feau	No changes made.	No changes made to the professional translation.	Answer: n<br>Strongly disagree (total nonsense): 0<br>Disagree: 0<br>Neutral: 0<br>Agree: 2<br>Strongly agree (totally understandable): 3<br>Did not answer due to technical issues: 1	Answer: n<br>Strongly disagree (totally dishonest): 0<br>Disagree: 0<br>Neutral: 0<br>Agree: 0<br>Strongly agree (totally honest/truthful): 5<br>Did not answer due to technical issues: 1	Not discussed in the focus group.
27	Relationships with your friends	No changes.	Relationships with your friends	Va ma au uo	No changes made.	We added the word "au" based on feedback from the adolescents during the focus group.	Answer: n<br>Strongly disagree (total nonsense): 0<br>Disagree: 1<br>Neutral: 0<br>Agree: 2<br>Strongly agree (totally understandable): 2<br>Did not answer due to technical issues: 1	Answer: n<br>Strongly disagree (totally dishonest): 0<br>Disagree: 0<br>Neutral: 1<br>Agree: 2<br>Strongly agree (totally honest/truthful): 2<br>Did not answer due to technical issues: 1	Discussed; asked adolescents: "*Any issue in understanding this question?*"<br><br>Adolescents identified a typo in the Samoan translation; our team made this

									correction for the final tool.
28	Praying	No changes.	Praying	Tatalo	No changes made.	No changes made to the professional translation.	Answer: n<br>Strongly disagree (total nonsense): 0<br>Disagree: 0<br>Neutral: 0<br>Agree: 3<br>Strongly agree (totally understandable): 2<br>Did not answer due to technical issues: 1	Answer: n<br>Strongly disagree (totally dishonest): 0<br>Disagree: 0<br>Neutral: 0<br>Agree: 3<br>Strongly agree (totally honest/truthful): 2<br>Did not answer due to technical issues: 1	Not discussed in the focus group.
29	Schoolwork	No changes.	Schoolwork	Meaaoga	No changes made.	No changes made to the professional translation.	Answer: n<br>Strongly disagree (total nonsense): 0<br>Disagree: 0<br>Neutral: 0<br>Agree: 2<br>Strongly agree (totally understandable): 3<br>Did not answer due to technical issues: 1	Answer: n<br>Strongly disagree (totally dishonest): 0<br>Disagree: 0<br>Neutral: 0<br>Agree: 2<br>Strongly agree (totally honest/truthful): 3<br>Did not answer due to technical issues: 1	Not discussed in the focus group.
30	Relationships with your family	No changes.	Relationships with your family	Va ma lou aiga	No changes made.	No changes made to the professional translation.	Answer: n<br>Strongly disagree (total nonsense): 0<br>Disagree: 0<br>Neutral: 0<br>Agree: 3<br>Strongly agree (totally understandable): 2<br>Did not answer due to technical issues: 1	Answer: n<br>Strongly disagree (totally dishonest): 0<br>Disagree: 0<br>Neutral: 0<br>Agree: 2<br>Strongly agree (totally honest/truthful): 3<br>Did not answer due to technical issues: 1	Not discussed in the focus group.
31	Being happy with your life	No changes.	Being happy with your life	Fiafia i lou olaga	No changes made.	No changes made to the professional translation.	Answer: n<br>Strongly disagree (total nonsense): 0<br>Disagree: 0<br>Neutral: 1<br>Agree: 1<br>Strongly agree (totally understandable): 3<br>Did not answer due to technical issues: 1	Answer: n<br>Strongly disagree (totally dishonest): 0<br>Disagree: 0<br>Neutral: 1<br>Agree: 0<br>Strongly agree (totally honest/truthful): 4<br>Did not answer due to technical issues: 1	Not discussed in the focus group.

Table H. Summary of the cross-culturally adapted version of the CPSS-V for Samoan adolescents, along with summary of changes to the English language wording. When adolescents completed multiple entries for the same answer (for example, if the adolescent completed the survey twice due to technical issues), we reported the Likert Scale answer for their most recent survey attempt but reported the qualitative quotations across all attempts.

	Suicide Questions for Samoan Adolescents					Justification and deliberation of English wording changes by the expert committee	Samoan translation and back-translation notes	Adolescent Pretesting		
								Survey (n=6)		Focus group (n=5)
	Original English	Source	Tracked Changes	Final English	Final Samoan			*Is this question easy to understand?*	*Would adolescents answer this question honestly?*	
0	Life has many ups and downs. Sometimes people feel upset. These feelings can be so bad that people who have them may feel suicidal or want to self-harm. The following questions ask you about your feelings and the feelings of people close to you. We know this is a sensitive subject, but it is important to ask about it now, as it is not uncommon. By finding out about self-harm we can find ways of helping people.	ALSPAC (14)	No changes.	Life has many ups and downs. Sometimes people feel upset. These feelings can be so bad that people who have them may feel suicidal or want to self-harm. The following questions ask you about your feelings and the feelings of people close to you. We know this is a sensitive subject, but it is important to ask about it now, as it is not uncommon. By finding out about self-harm we can find ways of helping people.	E tele taimi e lūga lālo ai le olaga. O isi taimi e lagona ai e tagata le lē fiafia. O nei lagona e iai taimi e matuā tugā ai, ma o'o ai i se tulaga e lagona ai e e ua aafia ai, le fia pule i le ola, pe faamanu'alia o ia lava. O fesili o i lalo, o le a fesiligia ai ou faalagona, ma lagona o tagata e latalata ia te oe. Matou te iloa e ma'ale'ale lenei mataupu ae taua pe a tatou fa'asoa i ai,āua e le o se mea fou. I le suesueina o le mataupu i le faamanu'alia o lou lava tagata, e mafai ai ona tatou maua ni auala e fesoasoani ai i ia tagata.	No changes made.	A few minor grammar edits to the professional translation.	Answer: n<br>Strongly disagree (total nonsense): 0<br>Disagree: 0<br>Neutral: 1<br>Agree: 2<br>Strongly agree (totally understandable): 2<br>Did not answer due to technical issues: 1	Answer: n<br>Strongly disagree (totally dishonest): 0<br>Disagree: 0<br>Neutral: 0<br>Agree: 2<br>Strongly agree (totally honest/truthful): 3<br>Did not answer due to technical issues: 1	Not discussed in the focus group.
SOCIAL NETWORKS										
1	Has anyone in your family (not including yourself) ever hurt themselves on purpose (e.g. by taking an overdose of pills, or by cutting themselves)?<br><br>Yes/No	ALSPAC (14)	No changes.	Has anyone in your family (not including yourself) ever hurt themselves on purpose (e.g. by taking an overdose of pills, or by cutting themselves)?<br><br>Yes/No	Sa iai se isi o le tou aiga (e le faitauina ai oe) na faamanu'alia e ia ia lava (faataitaiga, so'ona inu fuala'au po o le selei/tatipi o le tino)?<br><br>Ioe/Leai	No changes made.	No changes made to the professional translation.			
2	Have any of your close friends ever hurt themselves on purpose?<br><br>Yes/No	ALSPAC (14)	No changes.	Have any of your close friends ever hurt themselves on purpose?<br><br>Yes/No	E iai ni au uo mamae na faamanu'alia ma le mautinoa ia latou lava?<br><br>Ioe/Leai	No changes made.	No changes made to the professional translation.	Answer: n<br>Strongly disagree (total nonsense): 0<br>Disagree: 0<br>Neutral: 0<br>Agree: 3<br>Strongly agree (totally understandable): 2<br>Did not answer due to technical issues: 1	Answer: n<br>Strongly disagree (totally dishonest): 0<br>Disagree: 0<br>Neutral: 0<br>Agree: 3<br>Strongly agree (totally honest/truthful): 2<br>Did not answer due to technical issues: 1	Not discussed in the focus group.
3	Has anyone you have ever known died by suicide? If so, how many?	SITBI (15)	No changes.	Has anyone you have ever known died by suicide? If so, how many?	E iai se isi e te iloa na oti ona o le pule i le ola? Afai e iai, e to'afia?	No changes made.	No changes made to the professional translation.	Answer: n<br>Strongly disagree (total nonsense): 0<br>Disagree: 0<br>Neutral: 0<br>Agree: 1<br>Strongly agree (totally understandable): 4	Answer: n<br>Strongly disagree (totally dishonest): 0<br>Disagree: 0<br>Neutral: 0<br>Agree: 1<br>Strongly agree (totally honest/truthful): 4	Not discussed in the focus group.

								Did not answer due to technical issues: 1	Did not answer due to technical issues: 1	
SUICIDAL THOUGHTS										
4	How old were you the first time you had thoughts of killing yourself?	SITBI (15)	No changes.	How old were you the first time you had thoughts of killing yourself?	E fia ou tausaga i le uluai taimi na e mafaufau ai e te pule i lou ola?	No changes made.	No changes made to the professional translation.	Answer: n<br>Strongly disagree (total nonsense): 0<br>Disagree: 0<br>Neutral: 0<br>Agree: 2<br>Strongly agree (totally understandable): 3<br>Did not answer due to technical issues: 1	Answer: n<br>Strongly disagree (totally dishonest): 0<br>Disagree: 0<br>Neutral: 0<br>Agree: 2<br>Strongly agree (totally honest/truthful): 3<br>Did not answer due to technical issues: 1	Not discussed in the focus group.
5	How old were you the last time?	SITBI (15)	No changes.	How old were you the last time?	E fia ou tausaga i le taimi mulimuli na tupu ai?	No changes made.	No changes made to the professional translation.			
SELF-HARMING BEHAVIORS										
6	Have you ever hurt yourself on purpose in any way (e.g. by taking an overdose of pills, or by cutting yourself)?<br><br>Yes/No	ALSPAC (14)	No changes.	Have you ever hurt yourself on purpose in any way (e.g. by taking an overdose of pills, or by cutting yourself)?<br><br>Yes/No	Na iai se taimi na e faamanu'alia ai ma le mautinoa oe lava i so'o se auala (pei o le so'ona inu o fualaau po'o le tatipi o lou tino)?<br><br>Ioe/Leai	No changes made.	No changes made to the professional translation.	Answer: n<br>Strongly disagree (total nonsense): 0<br>Disagree: 1<br>Neutral: 0<br>Agree: 2<br>Strongly agree (totally understandable): 2<br>Did not answer due to technical issues: 1	Answer: n<br>Strongly disagree (totally dishonest): 0<br>Disagree: 1<br>Neutral: 0<br>Agree: 2<br>Strongly agree (totally honest/truthful): 2<br>Did not answer due to technical issues: 1	Discussed; asked adolescents: "*Any issue in understanding this question?*"<br><br>Consensus that it is understandable; no changes made.<br><br>We did not discuss honestly for this specific item in the focus group; rather, we asked the group questions to more generally improve honesty across all items.
7	If yes: How many times have you done this in the last year?<br><br>Please mark one box only.<br><br>Once/2-5 times/6-10 times/More than 10 times	ALSPAC (14)	No changes.	If yes: How many times have you done this in the last year?<br><br>Please mark one box only.<br><br>Once/2-5 times/6-10 times/More than 10 times	Afai e ioe: E faafia ona e faia lea tulaga i le tausaga ua tuana'i?<br><br>Faamolemole, maka na'o le pusa e tasi.<br><br>Faatasi/2-5/6-10/Sili atu ma le 10	No changes made.	No changes made to the professional translation.	Answer: n<br>Strongly disagree (total nonsense): 0<br>Disagree: 1<br>Neutral: 0<br>Agree: 2<br>Strongly agree (totally understandable): 2<br>Did not answer due to technical issues: 1	Answer: n<br>Strongly disagree (totally dishonest): 0<br>Disagree: 1<br>Neutral: 0<br>Agree: 2<br>Strongly agree (totally honest/truthful): 2<br>Did not answer due to technical issues: 1	Discussed; asked adolescents: "*Any issue in understanding this question?*"<br><br>Consensus that it is understandable; no changes made.<br><br>We did not discuss honestly for this specific item in the focus group; rather, we asked the group questions to more generally improve honesty across all items.

8	When was the last time you hurt yourself on purpose?<br><br>Please mark one box only.<br><br>In the last week/More than a week ago but in the last year/More than a year ago	ALSPAC (14)	No changes.	When was the last time you hurt yourself on purpose?<br><br>Please mark one box only.<br><br>In the last week/More than a week ago but in the last year/More than a year ago	O le a le taimi mulimuli na e faamanu'alia ai ma le mautinoa oe lava?<br><br>Faamolemole, maka na'o le pusa e tasi.<br><br>Vaiaso ua te'a/Ova atu ma le vaiaso ua tuana'i/Sili atu ma le tausaga	No changes made.	No changes made to the professional translation.	Answer: n<br>Strongly disagree (total nonsense): 0<br>Disagree: 1<br>Neutral: 0<br>Agree: 2<br>Strongly agree (totally understandable): 2<br>Did not answer due to technical issues: 1	Answer: n<br>Strongly disagree (totally dishonest): 1<br>Disagree: 0<br>Neutral: 1<br>Agree: 1<br>Strongly agree (totally honest/truthful): 2<br>Did not answer due to technical issues: 1<br><br>Why might adolescents not be honest in answering this question?<br>*"Scared of expressing themselves"*	Discussed; asked adolescents: "*Any issue in understanding this question?*"<br><br>Consensus that it is understandable; no changes made.
9	How old were you the last time you hurt yourself on purpose?	None, but adapted language from SITBI (15)	Not applicable.	How old were you the last time you hurt yourself on purpose?	E fia ou tausaga i le taimi mulimuli na e fuafuaina ai e faamanu'alia oe lava?	No changes made.	No changes made to the professional translation.	Answer: n<br>Strongly disagree (total nonsense): 0<br>Disagree: 0<br>Neutral: 1<br>Agree: 1<br>Strongly agree (totally understandable): 3<br>Did not answer due to technical issues: 1	Answer: n<br>Strongly disagree (totally dishonest): 0<br>Disagree: 1<br>Neutral: 1<br>Agree: 1<br>Strongly agree (totally honest/truthful): 2<br>Did not answer due to technical issues: 1	We did not discuss honestly for this specific item in the focus group; rather, we asked the group questions to more generally improve honesty across all items.
10	The last time you hurt yourself on purpose, which of the actions below best describes what you did?<br><br>Please mark all boxes that apply.<br><br>Swallowed pills or something poisonous/Cut yourself/Burnt yourself e.g. with cigarette/Somethin g else -> please say what:	ALSPAC (14) and SITBI (15)	The last time you hurt yourself on purpose, which of the actions below best describes what you did?<br><br>Please mark all boxes that apply.<br><br>Swallowed pills or something poisonous/Cut ~~yourself~~or carved skin/Burned ~~yourself~~your skin (i.e., with a cigarette, match or other hot object)/Inserted sharp objects into your skin or nails/Picked areas of your body to the point of drawing blood/Hit yourself on purpose/Scraped your skin to the point of drawing blood/Something else -> please say what:	The last time you hurt yourself on purpose, which of the actions below best describes what you did?<br><br>Please mark all boxes that apply.<br><br>Swallowed pills or something poisonous/Cut or carved skin/Burned your skin (i.e., with a cigarette, match or other hot object)/Inserted sharp objects into your skin or nails/Picked areas of your body to the point of drawing blood/Hit yourself on purpose/Scraped your skin to the point of drawing blood/Something else -> please say what:	I le taimi mulimuli na e faamanu'alia ai ma le mautinoa oe lava, o a gaoioiga o loo lisi atu i lalo e faamatalā lelei le mea sa e faia?<br><br>Faamolemole maka uma pusa e fetaui.<br><br>Inu ni fualaau poo se mea oona/Tatipi le pa'u o le tino/ Susunu lou tino (i se sikaleti, afitusi, poo se isi mea vevela)/Sulu ni mea maai i le pa'u o lou tino poo atigilima/Iini vaega o lou tino se'ia maligi le toto/Ta oe lava ma le mautinoa/Vavalu ese le pa'u o le tino se'ia maligi le toto/Se isi mea -> faamolemole faailoa mai poo le a:	Added additional options to align with Nock SITBI Short Form because it is more detailed and would better tease apart cutting, a common method of self-harm in this population.	No changes made to the professional translation.	Answer: n<br>Strongly disagree (total nonsense): 1<br>Disagree: 0<br>Neutral: 0<br>Agree: 2<br>Strongly agree (totally understandable): 2<br>Did not answer due to technical issues: 1	Answer: n<br>Strongly disagree (totally dishonest): 0<br>Disagree: 1<br>Neutral: 0<br>Agree: 2<br>Strongly agree (totally honest/truthful): 2<br>Did not answer due to technical issues: 1	Discussed; asked adolescents: "*Any issue in understanding this question?*"<br><br>Samoan is less clear but consensus is that it is okay because it will be presented with the English.<br><br>We did not discuss honestly for this specific item in the focus group; rather, we asked the group questions to more generally improve honesty across all items.

11	Do any of the following reasons help to explain why you hurt yourself on that occasion?<br><br>Please mark all boxes that apply.<br><br>I wanted to show how desperate I was feeling/I wanted to die/I wanted to punish myself/I wanted to frighten someone/I wanted to get relief from a terrible state of mind/Some other reason -> Please say what	ALSPAC (14)	No changes.	Do any of the following reasons help to explain why you hurt yourself on that occasion?<br><br>Please mark all boxes that apply.<br><br>I wanted to show how desperate I was feeling/I wanted to die/I wanted to punish myself/I wanted to frighten someone/I wanted to get relief from a terrible state of mind/Some other reason -> Please say what	E iai se mafuaaga o ta'ua i lalo e fesoasoani e faamatala le mafuaaga na e faamanu'alia ai oe lava i lena taimi?<br><br>Faamolemole maka le pusa e talafeagai.<br><br>Na ou mana'o e faaali o'u lagona fia maua se lavea'i/Na ou fia oti/Na ou mana'o e fa'asala a'u/Na ou mana'o e faafefe se isi/Na ou mana'o ou te mapu mai mafaufauga le lelei/Nisi mafuaaga -> faamolemole faailoa mai	No changes made.	No changes made to the professional translation.	Answer: n<br>Strongly disagree (total nonsense): 0<br>Disagree: 0<br>Neutral: 1<br>Agree: 1<br>Strongly agree (totally understandable): 3<br>Did not answer due to technical issues: 1	Answer: n<br>Strongly disagree (totally dishonest): 0<br>Disagree: 0<br>Neutral: 1<br>Agree: 1<br>Strongly agree (totally honest/truthful): 3<br>Did not answer due to technical issues: 1	Not discussed in focus groups.
12	After you had hurt yourself on that occasion, how did you feel?<br><br>Please mark one box only.<br><br>Better than before/The same as before/Worse than before	ALSPAC (14)	No changes.	After you had hurt yourself on that occasion, how did you feel?<br><br>Please mark one box only.<br><br>Better than before/The same as before/Worse than before	Ina ua mae'a ona faamanu'alia oe lava i lena taimi, o le a sou lagona na i ai?<br><br>Faamolemole maka le pusa e tasi.<br><br>Sili atu nai lo le taimi muamua/Tutusa ma le taimi muamua/Leaga atu i lo le taimi muamua	No changes made.	No changes made to the professional translation.	Answer: n<br>Strongly disagree (total nonsense): 0<br>Disagree: 0<br>Neutral: 1<br>Agree: 2<br>Strongly agree (totally understandable): 2<br>Did not answer due to technical issues: 1	Answer: n<br>Strongly disagree (totally dishonest): 0<br>Disagree: 1<br>Neutral: 1<br>Agree: 1<br>Strongly agree (totally honest/truthful): 2<br>Did not answer due to technical issues: 1	We did not discuss honestly for this specific item in the focus group; rather, we asked the group questions to more generally improve honesty across all items.
SUICIDE ATTEMPTS										
13	Have you ever made plans to kill yourself?	ALSPAC (14)	Have you ever made plans to kill yourself (for example, a specific plan towards where, when, and how)?	Have you ever made plans to kill yourself (for example, a specific plan towards where, when, and how)?	Na iai se taimi na e fuafua ai e te pule i lou ola (mo se faataitaiga, o le a sau fuafuaga na tapena i le nofoaga, le taimi ma le aso, ma e faapefea fo'i ona faataunuu)?	Adding clarification that we are asking for a specific plan (i.e., a specific idea of the what, when, and how).	No changes made to the professional translation.	Answer: n<br>Strongly disagree (total nonsense): 0<br>Disagree: 0<br>Neutral: 0<br>Agree: 2<br>Strongly agree (totally understandable): 3<br>Did not answer due to technical issues: 1	Answer: n<br>Strongly disagree (totally dishonest): 0<br>Disagree: 0<br>Neutral: 0<br>Agree: 3<br>Strongly agree (totally honest/truthful): 2<br>Did not answer due to technical issues: 1	Not discussed in the focus group.
14	Not applicable.	None; this question was developed by our team	Not applicable.	How many times did you try to kill yourself or make a suicide attempt?	E faafia ona e taumafai e fasioti oe, pe taumafai foi e pule i lou ola?	No changes made.	No changes made to the professional translation.	Answer: n<br>Strongly disagree (total nonsense): 0<br>Disagree: 0<br>Neutral: 1<br>Agree: 2<br>Strongly agree (totally understandable): 2<br>Did not answer due to technical issues: 1	Answer: n<br>Strongly disagree (totally dishonest): 0<br>Disagree: 0<br>Neutral: 1<br>Agree: 2<br>Strongly agree (totally honest/truthful): 2<br>Did not answer due to technical issues: 1	Not discussed in the focus group.
15	Not applicable.	None; this question was developed by our team	Not applicable.	What age(s) were you when you made your suicide attempt(s)?	O le fia o ou tausaga na e taumafai ai e te pule i lou ola?	No changes made.	No changes made to the professional translation.	Answer: n<br>Strongly disagree (total nonsense): 0<br>Disagree: 0<br>Neutral: 1<br>Agree: 2	Answer: n<br>Strongly disagree (totally dishonest): 0<br>Disagree: 0<br>Neutral: 1<br>Agree: 2	Not discussed in the focus group.

								Strongly agree (totally understandable): 2<br>Did not answer due to technical issues: 1	Strongly agree (totally honest/truthful): 2<br>Did not answer due to technical issues: 1	
HELP-SEEKING BEHAVIORS										
16	The last time you hurt yourself in any way (e.g. by taking an overdose of pills, or by cutting yourself) did you seek medical help/first aid from any of the following?<br><br>Please mark all boxes that apply.<br><br>GP (family doctor)/Hospital causality/Emergency department/Other health professional -> Please say what the job was	ALSPAC (14)	The last time you hurt yourself in any way (e.g. by taking an overdose of pills, or by cutting yourself) did you seek medical help/first aid from any of the following?<br><br>Please mark all boxes that apply.<br><br>Crisis phone line/Reaching out to a friend/Reaching out to a trusted adult/GP (family doctor)/Mental health professional/Hospital ~~causality/~~Emergency department/~~Other health professional~~ > Other -> Please say who this was	The last time you hurt yourself in any way (e.g. by taking an overdose of pills, or by cutting yourself) did you seek medical help/first aid from any of the following?<br><br>Please mark all boxes that apply.<br><br>Crisis phone line/Reaching out to a friend/Reaching out to a trusted adult/GP (family doctor)/Mental health professional/Hospital emergency department/Other -> Please say who this was	I le taimi mulimuli na e faamanu'alia ai oe lava i soo se auala (faataitaiga soona inu fualaau, poo le tatipi o lou tino, na e saili mo se fesoasoani i le falema'i, poo se isi lava pei ona lisi atu i lalo?<br><br>Faamolemole maka pusa e talafeagai.<br><br>Telefoni/laina fesoasoani/Fesoota'i i se uo/Fesootai i se tagata matua faatuatuaina/Foma'i (a le aiga)/Tagata tomai i le maloloina o le mafaufau/Matagaluega o ma'i faafuase'i i le Falema'i/Isi -> Faamolemole fa'ailoa mai poo ai lea	Adjusted options to better align with common points of initial contact for help-seeking behaviors, as identified in the adult qualitative interviews.	Minor semantical changes to the Samoan translation.	Answer: n<br>Strongly disagree (total nonsense): 0<br>Disagree: 0<br>Neutral: 1<br>Agree: 2<br>Strongly agree (totally understandable): 2<br>Did not answer due to technical issues: 1	Answer: n<br>Strongly disagree (totally dishonest): 0<br>Disagree: 0<br>Neutral: 2<br>Agree: 1<br>Strongly agree (totally honest/truthful): 2<br>Did not answer due to technical issues: 1	Not discussed in focus groups.
17	Have you ever tried to get help from someone or somewhere about hurting yourself on purpose, or about wanting to kill yourself?<br><br>Yes/No	ALSPAC (14)	No changes.	Have you ever tried to get help from someone or somewhere about hurting yourself on purpose, or about wanting to kill yourself?<br><br>Yes/No	Na iai se taimi na e taumafai e saili se fesoasoani mai se isi po o se nofoaga e uiga i lou faamanu'alia o oe lava, poo le fia pule fo'i i le ola?<br><br>Ioe/Leai	No changes made.	No changes made to the professional translation.	Answer: n<br>Strongly disagree (total nonsense): 0<br>Disagree: 0<br>Neutral: 0<br>Agree: 2<br>Strongly agree (totally understandable): 3<br>Did not answer due to technical issues: 1	Answer: n<br>Strongly disagree (totally dishonest): 0<br>Disagree: 0<br>Neutral: 0<br>Agree: 2<br>Strongly agree (totally honest/truthful): 3<br>Did not answer due to technical issues: 1	Not discussed in the focus group.
18	Who have you been to for help?<br><br>Please mark all boxes that apply.<br><br>Mum or Dad/Brother or sister/Someone else in your family/A friend/A teacher/A school counselor/Peer supporter/mediator at school/A GP	ALSPAC (14)	Who have you been to for help?<br><br>Please mark all boxes that apply.<br><br>~~Mum or Dad~~Mother/Father/~~Brother or sister~~/Brother/Sister/Someone else in your family/A friend/A teacher/A ~~High~~ school counselor/Peer	Who have you been to for help?<br><br>Please mark all boxes that apply.<br><br>Mother/Father/Brother/Sister/Someone else in your family/A friend/A teacher/High school counselor/Peer mentor/A GP (family doctor)/A social worker/A psychologist or psychiatrist/A telephone	O ai na e o'o i ai mo se fesoasoani?<br><br>Faamolemole maka uma pusa e talafeagai.<br><br>Tina/Tama/Tuagane/Tuafafine/Se isi o lou aiga/Uo/Faia'oga/Faufautua Aoga/Maualuga/Fesoasoani i augatupulaga/Foma'i a le aiga/Tagata i le galuega tauagafesootai/Tagata tomai tau saikolo ma le foma'i o le mafaufau/Laina	Adjusted options to better align with common points of initial contact for help-seeking behaviors, as identified in the adult qualitative interviews (1).	No changes made to the professional translation.	Answer: n<br>Strongly disagree (total nonsense): 0<br>Disagree: 0<br>Neutral: 0<br>Agree: 1<br>Strongly agree (totally understandable): 4<br>Did not answer due to technical issues: 1	Answer: n<br>Strongly disagree (totally dishonest): 0<br>Disagree: 0<br>Neutral: 0<br>Agree: 1<br>Strongly agree (totally honest/truthful): 4<br>Did not answer due to technical issues: 1	Not discussed in the focus group.

	(family doctor)/A social worker/A psychologist or psychiatrist/A telephone help line/Somewhere else (e.g. internet, book, magazine, other person, etc.) -> Please say what or who		supporter/mediator or at school mentor/A GP (family doctor)/A social worker/A psychologist or psychiatrist/A telephone ~~help~~crisis line/Drug dealer/Social media/Sports coach/Somewhere else (e.g. internet, book, magazine, other person, etc.)/Other trusted adult -> Please say what or who	crisis line/Drug dealer/Social media/Sports coach/Somewhere else (e.g. internet, book, magazine, other person, etc.)/Other trusted adult -> Please say what or who	telefoni Fesoasoani/Tagata faatau fualaau faasaina/Ala o faasalalauga a tagata faitele/Faiaoga tau taaloga/Se isi fesoasoani (faataitaiga Initaneti, tusi, mekasini, isi)/Se isi tagata matua Faatuatuaina -> Faamolemole faailoa mai poo le a poo ai					

Table I. Summary of the cross-culturally developed suicide questionnaire for Samoan adolescents, drawing on questions from the Self-Injurious Thoughts and Behaviors Interview (SITBI) (15) and Avon Longitudinal Study of Parents and Children (ALSPAC) self-harm questionnaire (14). When adolescents completed multiple entries for the same answer (for example, if the adolescent completed the survey twice due to technical issues), we reported the Likert Scale answer for their most recent survey attempt but reported the qualitative quotations across all attempts.

Source	Quotation	In reference to specific item
General barriers		
Focus group	So I believe that kids here won't want to put themselves on the spot or make them or anyone else feel like, 'I need help', cause some of them may feel like [their] problems are a burden to anyone else so they may not answer those questions honestly, but maybe if…the questions itself weren't as deep…we would all be able to answer the question honestly.	Prompted from a general discussion related to questions about sensitive topics such as sexual trauma, physical punishment, violence at home, and feelings of sadness, irritation, shame related to family.
PHQ-9M		
Survey	They feel as they can't quite express their feelings to anyone or even a stranger.	PHQ-9M; in general
Survey	Some of them have gone through in those kind of matters but doesn't have the confiden[ce] to share about it.	PHQ-9M #2: Not really interested in doing things or talking to people (such as not wanting to spend time with friends or participate in family or church activities)?
Survey	They don't want to be seen as weak or vulnerable.	
Survey	Sometimes they don't want to say the truth about the things that are going on.	PHQ-9M #7: Trouble concentrating on things like schoolwork, reading or watching TV?
Survey	They're going to lie so that they are positive about themselves but in reality they're not.	PHQ-9M 6: Feeling down about yourself — or feeling that you are a failure, or that you have disappointed yourself or your family?
Survey	Sometimes they can feel very embarrassed that they are telling people how they are feeling very insecure about themselves.	
Survey	Adolescents have a tendency of trying to hide the pain or hide their feelings.	PHQ-9M #9: Thoughts or feelings that you would be better off dead or hurting yourself in some way?
Survey	They feel as there is nothing they can do or there is nothing that person could do to fix what is wrong with them at all.	
Survey	They don't want to be bother at times.	PHQ-9M #10: In the past year have you felt depressed or sad most days, even if you felt okay sometimes?
Survey	Again they don't want the truth to come out.	PHQ-9M #12: Has there been a time in the past month when you have had serious thoughts about ending your life?
Survey	I don't think it's an easy thing to talk about so I believe most teens would just keep it to themselves.	
Survey	They are scared to admit that they have had a thought.	PHQ-9 #13: Have you ever, in your whole life, tried to kill yourself or made a suicide attempt?
Survey	It could really go either way that teens might lie or tell the truth.	
GAD-7		
Survey	They don't want to be mean and selfish.	GAD-7 #6: Becoming easily annoyed, irritable, or upset
Survey	Also the[y] lack confidence to speak up.	GAD-7 #7: Feeling afraid as if something terrible might happen (for instance, to yourself, your family, or others)
Trauma Screen		
Survey	Still the chances of them getting something worse or bad back.	Trauma Screen #6: Seeing a relative get severely physically hurt or punished, or beaten
Survey	Still trying to gain the courage to speak and share their story.	Trauma Screen #8: Being touched in your sexual/private parts in a way you didn't like or that made you feel uncomfortable by an adult/someone older who should not be touching you there
Survey	It can be hard for some to make sure to speak the truth about things.	
Survey	I think too many people would feel embarrassed or insecure if they talk about how they were sexually harassed.	
Focus group	Yeah I don't think so because you know…not feel very comfortable…share [their] story.	
Survey	Again the feeling of being afraid or fearing something will come back for her.	Trauma Screen #9: Being forced/pressured to have sex at a time when you could not say no
Focus group	Sexual trauma will be the one that they wouldn't be honest with you guys, unless you guys make them feel very very 100% comfortable, maybe they'll share with you guys.	Prompted from a general discussion related to questions about sexual trauma.
CPSS-V		
Survey	Too scared to admit the truth.	CPSS-V; in general

Survey	I feel like most teens would lie so that others would think that they have a healthy relationship with their families.	CPSS-V #16: Not feeling close to your friends or family or not wanting to be around them
Suicide Questions		
Survey	Scared of expressing themselves.	Suicide Questions #11: When was the last time you hurt yourself on purpose?

Table J. Examples of qualitative feedback from adolescents during either the pretesting survey or focus group that explained why adolescents might not report honest answers to specific questions. Six adolescents participated in the pilot testing survey; five adolescents participated in the focus group (n=10 adolescents participated in all activities; one adolescent participated in both methods).

REFERENCES

1. Mew EJ, Blas V, Winschel J, Hunt L, Soliai-Lemusu S, Johansson A, et al. "There are still broken or fragmented systems": Qualitative assessment of needs to strengthen adolescent mental health services in American Samoa. Int J Ment Health Nurs. 2024; 33(1):85-92.
2. National Center for Mental Health Checkups at Columbia University. TeenScreen Primary Care Screening Questionnaire Overview PSC-Y, PHQ-9, CRAFFT. Available from: https://depts.washington.edu/dbpeds/Screening%20Tools/TeenScreen.pdf.
3. PHQ-9: Modified for Teens. 2010. Available from: https://www.aacap.org/App_Themes/AACAP/docs/member_resources/toolbox_for_clinical_practice_and_outcomes/symptoms/GLAD-PC_PHQ-9.pdf
4. Plemmons G. Depression and Suicide Screening. In: Morelli V, editor. Adolescent Health Screening: An Update in the Age of Big Data. New York: Springer; 2019. p. 135-49.
5. Nandakumar AL, Vande Voort JL, Nakonezny PA, Orth SS, Romanowicz M, Sonmez AI, et al. Psychometric Properties of the Patient Health Questionnaire-9 Modified for Major Depressive Disorder in Adolescents. J Child Adolesc Psychopharmacol. 2019;29(1):34-40.
6. Aggarwal S, Taljard L, Wilson Z, Berk M. Evaluation of Modified Patient Health Questionnaire-9 Teen in South African Adolescents. Indian J Psychol Med. 2017;39(2):143-5.
7. Spitzer RL, Kroenke K, Williams JB, Löwe B. A brief measure for assessing generalized anxiety disorder: the GAD-7. Arch Intern Med. 2006;166(10):1092-7.
8. Casares MÁ, Díez-Gómez A, Pérez-Albéniz A, Lucas-Molina B, Fonseca-Pedrero E. Screening for anxiety in adolescents: Validation of the Generalized Anxiety Disorder Assessment-7 in a representative sample of adolescents. J Affect Disord. 2024;354:331-8.
9. Mossman SA, Luft MJ, Schroeder HK, Varney ST, Fleck DE, Barzman DH, et al. The Generalized Anxiety Disorder 7-item (GAD-7) scale in adolescents with generalized anxiety disorder: signal detection and validation. Ann Clin Psychiatry. 2017;29(4):227.
10. Osborn TL, Venturo-Conerly K, Gan J, Rodriguez M, Alemu R, Roe E, et al. Depression and Anxiety Symptoms Amongst Kenyan Adolescents: Psychometric Properties, Prevalence, Sociodemographic Factors, and Psychological Wellbeing. 2022; 50(11):1471-1485.
11. Foa EB, Asnaani A, Zang Y, Capaldi S, Yeh R. Psychometrics of the Child PTSD Symptom Scale for DSM-5 for trauma-exposed children and adolescents. J Clin Child Adolesc Psychol. 2018;47(1):38-46.
12. Meyer RM, Gold JI, Beas VN, Young CM, Kassam-Adams N. Psychometric evaluation of the child PTSD symptom scale in Spanish and English. Child Psychiatry Hum Dev. 2015;46(3):438-44.
13. Kohrt BA, Jordans MJD, Tol WA, Luitel NP, Maharjan SM, Upadhaya N. Validation of cross-cultural child mental health and psychosocial research instruments: adapting the Depression Self-Rating Scale and Child PTSD Symptom Scale in Nepal. BMC Psychiatry. 2011;11:127.
14. Kidger J, Heron J, Lewis G, Evans J, Gunnell D. Adolescent self-harm and suicidal thoughts in the ALSPAC cohort: a self-report survey in England. BMC Psychiatry. 2012;12:69.
15. Nock MK, Holmberg EB, Photos VI, Michel BD. Self-Injurious Thoughts and Behaviors Interview: Development, reliability, and validity in an adolescent sample. Psychol Assess. 2007;19(3):309-17.
