# Supplementary material for: Cross-cultural adaptation of mental health screening instruments for Samoan adolescents
Source: PLOS Ment Health. 2025 Feb 11;2(2):e0000106. doi: 10.1371/journal.pmen.0000106 (PMC12798219; doi:10.1371/journal.pmen.0000106)
Supplement: S1 Text — This file presents the adapted PHQ-9M questionnaire for Samoan adolescents, reformatted to align with the original screening instrument to facilitate administration. (PDF) [file pmen.0000106.s002.pdf]

Administering the PHQ-9M For Samoan Adolescents:

Samoan adolescents might not answer sensitive topics in questionnaires honestly. To promote honesty, before administering this questionnaire, please make an effort to build trust and create a safe space for the adolescent. Suggestions include:

- It is ideal if the questionnaire is administered by a person the adolescent does not already know (such as a stranger) and also speaks and understands the questionnaire in both English and Samoan to assist the adolescent to answer survey completely and truthfully.
- Administer the questionnaire in a private space, with either just you and the adolescent, or the adolescent alone.
- Before administering the questionnaire, take time to build trust and connection. This could include:
    - Asking the adolescent questions about their interests and actively listening to their answers
    - Employing a non-judgmental and warm demeanor
    - Being present, which includes giving the adolescent your full attention
    - Explaining that any information shared will be held confidential, and clearly communicating which instances under mandatory reporting requirements (if any) would require reporting information to their families
- Clearly communicate the intention behind the questionnaire (such as, to understand how common certain mental health problems are, or to understand what you are going through to help you feel better). Clearly communicate that the goal of asking them these questions is not to get them or anyone they know in trouble.
- Provide the adolescent the opportunity to ask questions before they begin the questionnaire.

E ono lē tali sa'o e tupulaga Samoa ni mataupu ma'ale'ale i pepa fesili. Ina ia u'unaia ona tali mai ma le faamaoni, faamolemole taumafai i se faiga e faatuatuaina ai ma iloa ai e tupulaga e saogalemu a latou faamatalaga ia te oe.
E mafai ona aofia ai fautuaga nei:

- *E pito sili pe afai e faatautaia le pepa fesili e se isi latou te lē iloa (e pei o se tagata ese), ma e tautala ma malamalama i le Pepa Fesili i le Igilisi ma le faa-Samoa, ina ia fesoasoani i le talavou ia atoatoa ma faamaoni a latou tali.*
- *Ia faatautaia le taliga o le pepa fesili i se nofoaga e le o tatalaina i le lautele, e na 'o oulua ma le talavou, pe na o ia fo'i*
- *A o le'i faatumua le pepa fesili, fai se lua taimi ia tupu ai lona faatuatuaina o oe ma fesoota'i lelei atu. E mafai ona aofia ai:*
    - *Lou fesili i ai i mea latou te fiafia i ai ma matuā faalologo lelei i a latou tali.*
    - *Ia faaalia lou lē faamasino tagata ma ni ou uiga mafanafana.*
    - *Ia iai ma latou, e aofia ai ma le tuu atoa i ai o lou loto i lou taimi ma le talavou*
    - *Faamalamalama i ai o soo se faamatalaga e tuu atu e le faailoā i se isi, ma ia manino lelei ni taimi (pe a iai) e ono lipoti ai ia faamatalaga i lona aiga*
- *Ia faailoa manino le mafuaaga o le pepa fesili (e pei o le fia malamalama poo le a le taatele o nisi o faafitauli tau le maloloina o le mafaufau o alia'e, poo le malamalama i se tulaga o e iai ina ia iloa le auala sili e fesoasoani atu ai ia suia i le lelei ou lagona). Ia manino ona faailoa atu, o le faamoemoe o fesili e lē ina ia aafia ai ia poo se isi latou te iloa.*
- *Tuu se avanoa i le talavou e fai mai ni fesili ae le'i amata ona tali le pepa fesili.*

The following pages were adapted from the original PHQ-9 Modified for Teens. The layout, scoring, and administrative guidelines are taken verbatim from the original instrument; questions and prompts were adapted and translated for Samoan adolescents. For more information on the adaptation process, please see Mew et al., 2024 (peer-reviewed publication in PLOS Mental Health).

A Survey From Your Healthcare Provider —
PHQ-9 Modified for Teens – Samoan Version

Name____Clinician____

Medical Record or ID Number_____________________________Date____

Instructions: How often have you been bothered by each of the following symptoms during the past two weeks? *I le lua vaiaso talu ai, e fa'afia ona e a'afia i auga ta'itasi nei?* For each symptom put an "X" in the box beneath the answer that best describes how you have been feeling. *Tusi se "X" i le pusa i lalo ane o le tali pito talafeagai e faamatala ai lou lagona.*

	(0) Not at all / *E leai ni auga*	(1) Several days / *Ni nai aso*	(2) More than half the days / *Sili atu ma le afa o aso*	(3) Nearly every day / *Toeitiiti lava o aso uma*
1. Feeling sad, irritable (for example, easily annoyed), or hopeless? *Lagona le faanoanoa, maitaita, (mo se faataitaiga, ita gofie) po ua leai se faamoemoe?*				
2. Not really interested in doing things or talking to people (such as not wanting to spend time with friends or participate in family or church activities)? *E faalefiafia tele i mea e fai poo le talanoa foi i tagata (e pei o le lē fia mafuta ma uo pe auai i mea e fai a le aiga poo le ekalesia fo'i)?*				
3. Trouble falling asleep, staying asleep, or sleeping too much? *Faigata ona moe, faigata ona faaauau le moe, pe moe so'o?*				
4. Poor appetite, weight loss, or overeating? *Tau le manogi se ai, alu le tino (lusi); po'o le soona'ai?*				
5. Feeling tired, or having little energy? *Lagona le lē lava, faapalupē/le lava le malosi?*				
6. Feeling down about yourself — or feeling that you are a failure, or that you have disappointed yourself or your family? *Faanoanoa ona o oe lava ia — poo lagona o oe o se tagata toi'lalo, pe ua e faalumaina oe ma lou aiga?*				
7. Trouble concentrating on things like schoolwork, reading or watching TV? *Faafaigata ona tuu atoa lou mafaufau i au meaaoga, faitaugatusi poo le matamata o le TV?*				
8. Moving or speaking so slowly that other people could have noticed? *Gaoioi poo le tautala lemu lava e ono amata ai ona iloa mai e isi?* Or the opposite — being so fidgety (for example, can't sit still) or restless that you were moving around a lot more than usual? *Poo le lē mafai ona nofo filemu pe gaoiā ma ua fealuai solo e sili atu nai lo le mea e masani ai?*				
9. Thoughts or feelings that you would be better off dead or hurting yourself in some way? *Mafaufauga poo faalogona e sili ai le oti poo le faia o nisi tulaga e faao'o ai le tiga o le tino ia te oe?*				

10. In the ***past year*** have you felt depressed or sad most days, even if you felt okay sometimes? *I le **tausaga ua mavae**, na e lagona ai le faanoanoa loloto poo le faanoanoa i le tele o aso, tusa pe na iai nisi taimi na lelei ai?*<br>☐ Yes / *Ioe* ☐ No / *Leai*
11. If you are experiencing any of the problems discussed/listed above, how **difficult** have these problems made it for you to do your schoolwork, take care of chores at home or get along with other people? *Afai o e lagonaina se faafitauli o fa'atalanoaina i lenei pepa, o le a se **faigata** na oo i ai i le faiga o au meaaoga i le fale, faatinoga o feau i le fale, poo le galulue faatasi ma isi?*<br>☐ Not difficult at all / *Leai se faigata* ☐ Somewhat difficult / *Faigata laitiiti*<br>☐ Very difficult / *Faigata tele* ☐ Extremely difficult / *Matuā faigata*
12. Has there been a time in the past month when you have had serious thoughts about ending your life? *Na iai se taimi i le masina ua tuanai na e manatu toto'a ai e te pule i lou ola?*<br>☐ Yes / *Ioe* ☐ No / *Leai*
13. Have you ***ever***, in your ***whole life***, tried to kill yourself or made a suicide attempt? *Na iai se taimi i lou **olaga atoa**, na e taumafai ai e pule i lou ola?*<br>☐ Yes / *Ioe* ☐ No / *Leai*

FOR OFFICE USE ONLY **Score** ___________

Q.12 and Q.13 = Y or TS = ≥11

Administering, Scoring, and Interpreting the PHQ-9 Screening Questionnaire

Administering

- The Patient Health Questionnaire Modified for Teens (PHQ-Modified) can be used with patients between the ages of 12 and 18 and takes less than five minutes to complete and score.
- The PHQ-9 Modified can be administered and scored by a nurse, medical technician, physical assistant, physician or other office staff.
- Patients should be left alone to complete the PHQ-9 Modified in a private area, such as an exam room or private area of the waiting room.
- Patients should be informed of their confidentiality rights before the PHQ-9 Modified is administered.
- The American Academy of Pediatrics and U.S. Preventive Service Task Force recommend that depression screening be conducted annually.

Scoring

- **For every X:**
  Not all = 0
  Several days = 1
  More than half the days = 2
  Nearly every day = 3
  Add up all “X” ed boxes on the screen
- **Defining a Positive Screen on the PHQ-9 Modified:**
  Total scores >/=11 are positive
- **Suicidality:**
  Regardless of the PHQ-9 Modified total score, endorsement of serious suicidal ideation OR past suicide attempt (question 12 and 13 on the screen) should be considered a positive screen.

Interpreting the Screening Results

- Patients that score positively on the questionnaire should be evaluated by their primary care provider (PCP) to determine if the depression symptoms they endorsed on the screen are significant, causing impairment and/or warrant a referral to a mental health specialist or follow-up treatment by the PCP.
- It is recommended that the PCP inquire about suicidal thoughts and previous suicide attempts with all patients that score positive, regardless of how they answered these items on the PDQ-9 Modified.
- For patients who score negative on the PHQ-9 Modified, it Is recommended that the PCP briefly review the symptoms marked as “more than half days” and “nearly every day” with the patient.
- The questionnaire indicates only the likelihood that a youth is at risk for depression or suicide; its results are not a diagnosis or a substitute for clinical evaluation.

Depression Severity

- The overall score on the PHQ-9 Modified provides information about the severity of depression, from minimal depression to severe depression.
- The interview with the patient should focus on their answers to the screen and the specific symptoms with which they are having difficulties.
- Additional questions on the PHQ-9 Modified also explore persistent depressive disorder, impairment of depressive symptoms, recent suicide ideation and previous suicide attempts.
- Interpretation of Total Score

**Total Score**	**Depression Severity**
1-4	Minimal depression
5-9	Mild depression
10-14	Moderate depression
15-19	Moderately severe depression
20-27	Severe depression
