# Supplementary material for: Cross-cultural adaptation of mental health screening instruments for Samoan adolescents
Source: PLOS Ment Health. 2025 Feb 11;2(2):e0000106. doi: 10.1371/journal.pmen.0000106 (PMC12798219; doi:10.1371/journal.pmen.0000106)
Supplement: S2 Text — This file presents the adapted GAD-7 questionnaire for Samoan adolescents, reformatted to align with the original screening instrument to facilitate administration. (PDF) [file pmen.0000106.s003.pdf]

Administering the GAD-7 for Samoan adolescents:

Samoan adolescents might not answer sensitive topics in questionnaires honestly. To promote honesty, before administering this questionnaire, please make an effort to build trust and create a safe space for the adolescent. Suggestions include:

- It is ideal if the questionnaire is administered by a person the adolescent does not already know (such as a stranger) and also speaks and understands the questionnaire in both English and Samoan to assist the adolescent to answer survey completely and truthfully.
- Administer the questionnaire in a private space, with either just you and the adolescent, or the adolescent alone.
- Before administering the questionnaire, take time to build trust and connection. This could include:
  - Asking the adolescent questions about their interests and actively listening to their answers
  - Employing a non-judgmental and warm demeanor
  - Being present, which includes giving the adolescent your full attention
  - Explaining that any information shared will be held confidential, and clearly communicating which instances under mandatory reporting requirements (if any) would require reporting information to their families
- Clearly communicate the intention behind the questionnaire (such as, to understand how common certain mental health problems are, or to understand what you are going through to help you feel better). Clearly communicate that the goal of asking them these questions is not to get them or anyone they know in trouble.
- Provide the adolescent the opportunity to ask questions before they begin the questionnaire.

E ono lē tali sa'o e tupulaga Samoa ni mataupu ma'ale'ale i pepa fesili. Ina ia u'unaia ona tali mai ma le faamaoni, faamolemole taumafai i se faiga e faatuatuaina ai ma iloa ai e tupulaga e saogalemu a latou faamatalaga ia te oe.
E mafai ona aofia ai fautuaga nei:

- *E pito sili pe afai e faatautaia le pepa fesili e se isi latou te lē iloa (e pei o se tagata ese), ma e tautala ma malamalama i le Pepa Fesili i le Igilisi ma le faa-Samoa, ina ia fesoasoani i le talavou ia atoatoa ma faamaoni a latou tali.*
- *Ia faatautaia le taliga o le pepa fesili i se nofoaga e le o tatalaina i le lautele, e na 'o oulua ma le talavou, pe na o ia fo'i.*
- *A o le'i faatumua le pepa fesili, fai se lua taimi ia tupu ai lona faatuatuaina o oe ma fesoota'i lelei atu. E mafai ona aofia ai:*
  - *Lou fesili i ai i mea latou te fiafia i ai ma matuā faalologo lelei i a latou tali.*
  - *Ia faaalia lou lē faamasino tagata ma ni ou uiga mafanafana.*
  - *Ia iai ma latou, e aofia ai ma le tuu atoa i ai o lou loto i lou taimi ma le talavou*
  - *Faamalamalama i ai o soo se faamatalaga e tuu atu e le faailoā i se isi, ma ia manino lelei ni taimi (pe a iai) e ono lipoti ai ia faamatalaga i lona aiga*
- *Ia faailoa manino le mafuaaga o le pepa fesili (e pei o le fia malamalama poo le a le taatele o nisi o faafitauli tau le maloloina o le mafaufau o alia'e, poo le malamalama i se tulaga o e iai ina ia iloa le auala sili e fesoasoani atu ai ia suia i le lelei ou lagona). Ia manino ona faailoa atu, o le faamoemoe o fesili e lē ina ia aafia ai ia poo se isi latou te iloa.*
- *Tuu se avanoa i le talavou e fai mai ni fesili ae le'i amata ona tali le pepa fesili.*

The following pages were adapted from the original Generalized Anxiety Disorder 7-item (GAD-7). The layout, scoring, and administrative guidelines are taken verbatim from the original instrument; questions and prompts were adapted and translated for Samoan adolescents. For more information on the adaptation process, please see Mew et al., 2024 (peer-reviewed publication in PLOS Mental Health).

GAD-7 Anxiety – Samoan Version

Over the <u>last two weeks</u>, how often have you been bothered by the following problems? *I le lua vaiaso talu ai, e fa'afia ona faapopoleina oe i fa'afitauli o loo sosoo atu?*	Not at all / *Leai ma se taimi*	Several days / *Mo ni nai aso*	More than half the days / *Sili atu i le afa o aso atoa*	Nearly every day / *Toeitiiti aso uma*
1. Feeling nervous, anxious, stressed or on edge<br>*Lagona lē to'a, popole; mamafa se mea i le mafaufau/atuatuvale*	0	1	2	3
2. Not being able to stop or control worrying<br>*Lē mafai ona taofi pe faatonutonu le lagona popole*	0	1	2	3
3. Worrying too much about different things (such as your future, disappointing your family/how other people might judge you, your responsibilities at home, school, and/or church, etc.)<br>*Soona popole i mea eseese (faapei o lou lumanai, faanoanoa i le faamasino tagata o lou aiga poo isi tagata i au matafaioi i lou aiga, aoga, ma/poo le ekalesia, ma isi.)*	0	1	2	3
4. Trouble relaxing<br>*Faafaigata ona faato'afilemu*	0	1	2	3
5. Being so restless that it is hard to sit still<br>*Ua matua lē to'afimalie ma ua i'u ina faigata ai ona mau pea le nofo*	0	1	2	3
6. Becoming easily annoyed, irritable, or upset<br>*Ua maitaita, itagofie poo le lē fiafia*	0	1	2	3
7. Feeling afraid as if something terrible might happen (for instance, to yourself, your family, or others)<br>*Lagona le fefe i se mea matautia e ono tula'i mai (fa'ataitaiga, ia te oe, lou aiga, po'o nisi tagata)*	0	1	2	3

Column totals _____ + ______ + ______ + ______ =

Total score ______

If you checked any of the problems discussed/listed above, how difficult have they made it for you to do your schoolwork, take care of chores at home, or get along with other people?
Afai o e lagonaina se faafitauli o fa'atalanoaina i lenei pepa, o le a se faigata na oo i ai i le faiga o au meaaoga i le fale, faatinoga o feau i le fale, poo le galulue faatasi ma isi?

Not difficult at all / *Leai se faigata*	Somewhat difficult / *Faigata laitiiti*	Very difficult / *Faigata tele*	Extremely difficult / *Matuā faigata*
☐	☐	☐	☐

Source: Primary Care Evaluation of Mental Disorders Patient Health Questionnaire (PRIME-MD-PHQ). The PHQ was developed by Drs. Robert L. Spitzer, Janet B.W. Williams, Kurt Kroenke, and colleagues. For research information, contact Dr. Spitzer at ris8@columbia.edu. PRIME-MD® is a trademark of Pfizer Inc. Copyright© 1999 Pfizer Inc. All rights reserved. Reproduced with permission.

Scoring GAD-7 Anxiety Severity

This is calculated by assigning scores of 0, 1, 2, and 3 to the response categories, respectively, of “not at all,” “several days,” “more than half the days,” and “nearly every day.”
GAD-7 total score for the seven items ranges from 0 to 21.

0–4: minimal anxiety

5–9: mild anxiety

10–14: moderate anxiety

15–21: severe anxiety
