# Supplementary material for: Cross-cultural adaptation of mental health screening instruments for Samoan adolescents
Source: PLOS Ment Health. 2025 Feb 11;2(2):e0000106. doi: 10.1371/journal.pmen.0000106 (PMC12798219; doi:10.1371/journal.pmen.0000106)
Supplement: S3 Text — This file presents the adapted CPSS-5 and Trauma Screen questionnaire for Samoan adolescents, reformatted to align with the original screening instrument to facilitate administration. (PDF) [file pmen.0000106.s004.pdf]

Administering the CPSS-5 and Trauma Screen for Samoan adolescents:

Samoan adolescents might not answer sensitive topics in questionnaires honestly. To promote honesty, before administering this questionnaire, please make an effort to build trust and create a safe space for the adolescent. Suggestions include:

- It is ideal if the questionnaire is administered by a person the adolescent does not already know (such as a stranger) and also speaks and understands the questionnaire in both English and Samoan to assist the adolescent to answer survey completely and truthfully.
- Administer the questionnaire in a private space, with either just you and the adolescent, or the adolescent alone.
- Before administering the questionnaire, take time to build trust and connection. This could include:
    - Asking the adolescent questions about their interests and actively listening to their answers
    - Employing a non-judgmental and warm demeanor
    - Being present, which includes giving the adolescent your full attention
    - Explaining that any information shared will be held confidential, and clearly communicating which instances under mandatory reporting requirements (if any) would require reporting information to their families
- Clearly communicate the intention behind the questionnaire (such as, to understand how common certain mental health problems are, or to understand what you are going through to help you feel better). Clearly communicate that the goal of asking them these questions is not to get them or anyone they know in trouble.
- Provide the adolescent the opportunity to ask questions before they begin the questionnaire.

E ono lē tali sa'o e tupulaga Samoa ni mataupu ma'ale'ale i pepa fesili. Ina ia u'unaia ona tali mai ma le faamaoni, faamolemole taumafai i se faiga e faatuatuaina ai ma iloa ai e tupulaga e saogalemu a latou faamatalaga ia te oe. E mafai ona aofia ai fautuaga nei:

- *E pito sili pe afai e faatautaia le pepa fesili e se isi latou te lē iloa (e pei o se tagata ese), ma e tautala ma malamalama i le Pepa Fesili i le Igilisi ma le faa-Samoa, ina ia fesoasoani i le talavou ia atoatoa ma faamaoni a latou tali.*
- *Ia faatautaia le taliga o le pepa fesili i se nofoaga e le o tatalaina i le lautele, e na 'o oulua ma le talavou, pe na o ia fo'i.*
- *A o le'i faatumua le pepa fesili, fai se lua taimi ia tupu ai lona faatuatuaina o oe ma fesoota'i lelei atu. E mafai ona aofia ai:*
    - *Lou fesili i ai i mea latou te fiafia i ai ma matuā faalologo lelei i a latou tali.*
    - *Ia faaalia lou lē faamasino tagata ma ni ou uiga mafanafana.*
    - *Ia iai ma latou, e aofia ai ma le tuu atoa i ai o lou loto i lou taimi ma le talavou*
    - *Faamalamalama i ai o soo se faamatalaga e tuu atu e le faailoā i se isi, ma ia manino lelei ni taimi (pe a iai) e ono lipoti ai ia faamatalaga i lona aiga*
- *Ia faailoa manino le mafuaaga o le pepa fesili (e pei o le fia malamalama poo le a le taatele o nisi o faafitauli tau le maloloina o le mafaufau o alia'e, poo le malamalama i se tulaga o e iai ina ia iloa le auala sili e fesoasoani atu ai ia suia i le lelei ou lagona). Ia manino ona faailoa atu, o le faamoemoe o fesili e lē ina ia aafia ai ia poo se isi latou te iloa.*
- *Tuu se avanoa i le talavou e fai mai ni fesili ae le'i amata ona tali le pepa fesili.*

The following pages were adapted from the original CPSS-V and its corresponding Trauma Screen. The layout, scoring, and administrative guidelines are taken verbatim from the original instrument; questions and prompts were adapted and translated for Samoan adolescents. For more information on the adaptation process, please see Mew et al., 2024 (peer-reviewed publication in PLOS Mental Health).

THE CHILD PTSD SYMPTOM SCALE FOR DSM-5 – Samoan Version

(CPSS-5 SR)

PSYCHOMETRIC PROPERTIES SUMMARY

The CPSS-SR-5 is a modified version of Child PTSD Symptom Scale self-report (CPSS-SR) for DSM-5. The 20 PTSD symptom items are rated on a 5-point scale of frequency and severity from 0 (not at all) to 4 (6 or more times a week /severe). The 7 functioning items are rated on yes/no.

Use the 20 symptom items to calculate a total symptom severity score. The CPSS-SR-5 has excellent internal consistency for total symptom severity (Cronbach's alpha = .924) and good test-retest reliability (r = .800). The CPSS-SR-5 also demonstrates convergent validity with CPSS-I-5 (r = .904), and discriminant validity with the Multidimensional Anxiety Scale (MASC) for Children and Child Depression Inventory (CDI). A cut off score of 31 can be used for identifying a probable PTSD diagnosis in children. In sum, the CPSS- SR-5 is a valid and reliable self-report instrument for assessing DSM-5 PTSD diagnosis and severity for children and adolescents.

CPSS SYMPTOM SEVERITY RANGES

Symptom Severity	Range
Minimal	0-10
Mild	11-20
Moderate	21-20
Severe	41-60
Very Severe	61-80

Note: We have included on the following page a trauma screen checklist in the event the clinician would find this helpful prior to doing the CPSS-V-SR. Completing it is optional.

TRAUMA SCREEN – Samoan Version (OPTIONAL – IF NEEDED)

Name: ____ Date: __________________________

INSTRUCTIONS

Many children and adolescents go through frightening or stressful events. Below is a list of frightening or stressful events that can happen. *O le toatele o tamaiti ma talavou (laiti), e a'afia i ni tulaga e mafua ai ona fefefe ma atuatuvale e o'o i ni tulaga e fefefe ma atuatuvale ai.* Mark YES if you have experienced any of these events. Mark NO if you have not experienced these events. *O loo i lalo atu le lisi o ni mea e tutupu e ono fefefe ma atuatuvale ai. Faailoga le IOE pe afai na e o'o i nisi o nei faafitauli. Faailoga le LEAI pe afai e te le'i o'o i ai.*

	Yes / *Ioe*	No / *Leai*
1. A severe natural disaster such as a tsunami, hurricane, or fire<br>*O se fa'alavelave fa'alenatura mata'utia e pei o galulolo/sunami, afā, po'o se mū*	☐	☐
2. Serious accident or injury caused by a car or bike crash, being seriously injured by a dog, or serious injury from sports<br>*Fa'alavelave mata'utia po'o se manu'a e mafua mai i le lavea i se ta'avale po o se uila vili vae, pe se manu'a tigaina ona o se maile, pe mafua fo'i i ni ta'aloga*	☐	☐
3. Being robbed by threat, force, or weapon<br>*Na faoa ni au mea totino i le tau faamata'u, ave fa'amalosi pe i se auupega malosi*	☐	☐
4. Being severely physically hurt or punished, or beaten by a relative<br>*Na matuā afaina, fa'asala, pe na fasi fo'i e se isi o le aiga*	☐	☐
5. Being severely physically hurt, knifed, or beaten by a stranger<br>*Na matuā afaina, po, afaina i se naifi/polo , pe fasi e se tagata ese*	☐	☐
6. Seeing a relative get severely physically hurt, punished, or beaten<br>*Vaai i se isi o lona auaiga o matuā afaina, fa'asala pe fasi*	☐	☐
7. Seeing somebody in your community being severely hurt, severely physically punished, or beaten<br>*Vaai i se isi o lona nuu o matuā afaina, fa'asala pe fasi*	☐	☐
8. Being touched in your sexual/private parts in a way you didn't like or that made you feel uncomfortable by an adult/someone older who should not be touching you there<br>*Tagofia e se isi po'o se tagata matua ou itūtino sa e le tatau ona ia tagofia*	☐	☐
9. Being forced/pressured to have sex at a time when you could not say no	☐	☐

Fa'amalosia e faia faigā aiga fa'amalosi i le taimi ua le mafai ona e fai atu i ai e leai		
10. A family member or somebody close dying suddenly or in a violent way<br>*Se isi o le auaiga ua oti fa'afuase'i pe i se auala saua*	☐	☐
11. Being attacked, shot, stabbed, or seriously injured<br>*Osofai'a, fana, tui po'o le manu'a tigāina*	☐	☐
12. Seeing someone be attacked, shot, stabbed, or seriously injured or killed<br>*Vaai i se isi o osofai'a, fana, tui pe manu'a tigaina pe ua fasiotia*	☐	☐
13. Having a stressful or frightening medical procedure (e.g., heart or brain surgery)<br>*Fefe po'o le atuatuvale i fa'agasologa o talavai a le falema'i (fa'ataitaiga taotoga o le fatu poo le fai'ai)*	☐	☐
14. Being around a war, meaning a state of armed conflict that can result in death (which is not the same as inter-village conflicts)<br>*Sa e auai i se taua e pei o tulaga o feteenaiga faaauupegaina e mafai ona i'u ai i le oti (e ese mai i feeseeseaiga i totonu o nuu)*	☐	☐
15. Any other stressful or frightening event that has not been included above<br>*So'o se isi mea na tupu e ono mafua ai le atuatuvale ma le fefe e le'o aofia i luga*<br><br>Describe / *Fa'amatala*:<br>____	☐	☐

Which of these events listed above bothers you most?
le fea o vaega nei o lisi atu i luga e pito sili ona fa'alavelave ia te oe?

____

If you answered **NO** to all of the above questions, **STOP**. If you answered **YES** to any of the above questions, please answer the following questions. Afai na e tali **LEAI** i fesili uma o lo'o i luga, **TAOFI**. Afai na e tali **IOE** i so'o se fesili lava o lo'o i luga, fa'amolemole tali fesili o lo'o mulimuli mai.

When the event happened, did you feel:<br>*I le taimi na tupu ai le fa'alavelave, na e lagonaina:*	Yes<br>*/ Ioe*	No<br>*/ Leai*
Fear that you were going to die or be seriously injured?<br>*Fefe ona o le a e oti pe manu'a tigaina?*		
Fear that some else was seriously hurt?<br>*Fefe e iai se isi na manu'a tigaina?*		
Unable to help yourself?<br>*Le mafai ona e fesoasoani ia te oe lava?*		
Shame or disgust?<br>*Māasiasi po'o le inoino?*		

CPSS – 5 – Samoan Version

Name or ID: ____ Date: ___________________

Sometimes scary or upsetting things happen in your life. It might be something like getting beaten up, living through a tsunami, witnessing violence at home, being touched in a way you didn't like or that made you feel uncomfortable, having a parent get hurt or killed, or some other very upsetting event. *E iai taimi e tutupu ai mea e fefe ai pe e te faanoanoa ai i lou olaga. E pei o le fasi o oe, sao mai se galulolo sa tupu, molimauina o sauaga i totonu o le aiga, tagofia o oe i se auala na e le mana'o ai ma e le filemu ai, fa'amanu'alia o se matua pe ua maliu foi, poo se isi lava fa'alavelave matuia.*

Please write down the scary or upsetting thing that bothers you the most when you think about it or the thing that you try not to think about (this should be the event you listed in the Trauma Screen): *Fa'amolemole tusi i lalo le fa'afitauli na pito sili ona e fefe ai, pe sa tele ina fa'afatu'ulu (e.g. fa'alavelave) ia te oe, pe a e mafaufau iai, poo se mea oloo e taumafai e aua e te mafaufau iai (o le fa'alavelave lea na 'e lisia i le Iloiloga o ni a'afiaga talu ai se fa'afitauli matuia, pe afai na faaaoga Trauma Screen):*

When did it happen? *O anafea na tupu ai?*

____

0	1	2	3	4
Not at all / *E leai lava*	Once a week or less/a little / *E faatasi i le vaiaso pe le tele fo'i*	2 to 3 times a week/somewhat / *E faalua pe faatolu i le vaiaso/e feoloolo lava*	4 to 5 times a week/a lot / *E faafa pe faalima i le vaiaso/e tele taimi*	6 or more times a week/almost always / *E faaono pe sili atu i le vaiaso/toe lava o taimi uma*

These questions ask about how you feel about the upsetting thing you wrote down. Read each question carefully. Then circle the number (0-4) that best describes how often that problem has bothered you IN THE LAST MONTH. *O fesili nei o lo'o fesiligia ai ou fa'alogona ina ua e tusia le mea sa e faanoanoa ai. Faitau lelei fesili taitasi. Li'o le fuainumera (0-4) e te iloa o lo'o faamatala lelei mai ai pe na faafia ona faafatu'ulu ia te oe lena faalavelave na tupu I LE MASINA UA TE'A.*

1. Having upsetting* thoughts or pictures about it that came into your head when you didn't want them to (*Upsetting means that they made you feel unhappy or worried)<br>*Iai fa'alogona le fiafia* po'o ni ata fa'atatau i ia fa'alogona e o'o mai i lou mafaufau ae e te le'i mana'o ai (*O faalogona le fiafia ua faauigaina na o'o ai ina e fa'anoanoa pe popole)*	0	1	2	3	4
2. Having bad dreams or nightmares<br>*Faia ni miti lē lelei poo miti taufaafefe*	0	1	2	3	4
3. Acting or feeling as if it was happening again (seeing or hearing something and feeling as if you are there again)<br>*Fa'atinoga po'o ni fa'alogona pei ua toe tupu fo'i (vaaia pe lagona se mea ma fa'alogona pei ua toe tupu fo'i)*	0	1	2	3	4
4. Feeling upset when you remember what happened (for example, feeling scared, angry, sad, guilty, confused)<br>*Fa'alogona lē fiafia pe a e toe manatua se mea na tupu (fa'ata'ita'iga, fa'alogona fefe, ita, fa'anoanoa, ta'usala, lē mautonu)*	0	1	2	3	4
5. Having feelings in your body when you remember what happened (for example, sweating, heart beating fast, stomach or head hurting)<br>*Iai fa'alogona o lou tino pe a toe manatua se mea na tupu (fa'ata'ita'iga, afu, vave le tātā o le fatu, tigā le manava po'o le ulu)*	0	1	2	3	4
6. Trying not to think about it or have feelings about it<br>*O loo taumafai e aua le mafaufau i ai, pe iai ni lagona e faatatau i ai*	0	1	2	3	4
7. Trying to stay away from anything that reminds you of what happened (for example, people, places, or conversations about it)<br>*Taumafai e 'alo ese mai so'o se mea e toe fa'amanatu atu ai ia te oe le mea na tupu (fa'ata'ita'iga, tagata, nofoaga, po'o se talanoaga fa'atatau iai)*	0	1	2	3	4
8. Not being able to remember an important part of what happened<br>*Lē mafai ona toe manatua se vaega taua o le mea na tupu*	0	1	2	3	4
9. Having bad thoughts about yourself, other people, or the world (for example, "I can't do anything right", "All people are bad", "The world is a scary place")<br>*Fa'alogona leaga fa'atatau ia te oe lava, isi tagata, po'o le lalolagi (fa'ata'ita'iga, "E leai se mea sa'o ou te faia", "O tagata uma e leaga", "O le lalolagi o se nofoaga taufa'afefe")*	0	1	2	3	4

0	1	2	3	4
Not at all / *E leai lava*	Once a week or less/a little / *E faatasi i le vaiaso pe le tele fo'i*	2 to 3 times a week/somewhat / *E faalua pe faatolu i le vaiaso/e feoloolo lava*	4 to 5 times a week/a lot / *E faafa pe faalima i le vaiaso/e tele taimi*	6 or more times a week/almost always / *E faaono pe sili atu i le vaiaso/toe lava o taimi uma*

10. Thinking that what happened is your fault (for example, "I should have known better", "I shouldn't have done that", "I deserved it")<br>*Mafaufauga o oe e mafua ai le mea na tupu (fa'ata'ita'iga, "Sa tatau ona ou iloa lelei", "Sa lē tatau ona ou faia ", "O le mea lena ou te maua")*	0	1	2	3	4
11. Having strong bad feelings (like fear, anger, guilt, or shame)<br>*Malosi ni lagona lē lelei (pei o le fefe, ita, lagona le sesē, po'o le maasiasi)*	0	1	2	3	4
12. Having much less interest in doing things you used to do (for example, spending time with friends, playing games, etc.)<br>*Ua itiiti atu le fiafia e fa'atino ni galuega sa masani ai (fa'ata'itaiga: evaga ma uo, ta'aloga)*	0	1	2	3	4
13. Not feeling close to your friends or family or not wanting to be around them<br>*Lē lagonaina le vavalalata i uo ma aiga pe le fiafia fo'i e fa'atasi ma i latou*	0	1	2	3	4
14. Trouble having good feelings (like happiness or love) or trouble having any feelings at all<br>*Faigatā ona maua ni lagona lelei (pei o le fiafia po'o le alofa) pe faigatā ona maua ni lagona*	0	1	2	3	4
15. Getting angry easily (for example, yelling, hitting others, throwing things)<br>*Maitaita gofie (fa'ata'ita'iga, e'ē, pa'ilima i isi, taua'i solo ni mea)*	0	1	2	3	4
16. Doing things that might hurt yourself (for example, taking drugs, drinking alcohol, running away, cutting yourself)<br>*Faia o ni mea e ono a'afia ai oe (fa'ata'ita'iga, tagofia o fuala'au fa'asaina, inu ava malosi, sola ese, tasele pe fa'amanu'aina oe lava)*	0	1	2	3	4
17. Being very careful or on the lookout for danger (for example, checking to see who is around you and what is around you)<br>*Fa'aete'ete tele po'o le va'ava'ai toto'a pe iai se tulaga faigata poo se faalavelave (e ono tupu) (fa'ata'ita'iga, siakiina po'o ai o i ou autafa, ae po'o a ni mea o siomia ai oe)*	0	1	2	3	4
18. Being jumpy or easily scared (for example, when someone walks up behind you, when you hear a loud noise)<br>*Mate'ite'i pe fefe gofie (fa'ata'ita'iga, pe a savali atu i ou tua se tagata, pe a lagonaina se pa'ō leotele)*	0	1	2	3	4
19. Having trouble paying attention (for example, losing track of a story on TV, forgetting what you read, unable to pay attention in class)<br>*Faafaigata ona ua'i le mafaufau (fa'ata'ita'iga, lē mulimuli lelei i se tala (o matamata ai) i le TV, galo se mea na e faitau iai, le mafai ona ua'i le fa'alogo i le aoga)*	0	1	2	3	4
20. Having trouble falling or staying asleep<br>*Faigatā ona moe pe faaauau le moe*	0	1	2	3	4

Have the problems above been getting in the way of these parts of your life IN THE PAST MONTH?
Faamata na avea faafitauli (o ta'ua) i luga atu ma faalavelave i vaega nei o lou olaga i le MASINA UA TUANA'I?

YES / *IOE*	NO / *LEAI*	21. Fun things you want to do<br>*O mea faafiafia (loto) e te mana'o e fai*
YES / *IOE*	NO / *LEAI*	22. Doing your chores<br>*Faiga o au feau*
YES / *IOE*	NO / *LEAI*	23. Relationships with your friends<br>*Va ma au uo*
YES / *IOE*	NO / *LEAI*	24. Praying<br>*Tatalo*
YES / *IOE*	NO / *LEAI*	25. Schoolwork<br>*Meaaoga*
YES / *IOE*	NO / *LEAI*	26.Relationships with your family<br>*Va ma lou aiga*
YES / *IOE*	NO / *LEAI*	27.Being happy with your life<br>*Fiafia i lou olaga*
