# Supplementary material for: Cross-cultural adaptation of mental health screening instruments for Samoan adolescents
Source: PLOS Ment Health. 2025 Feb 11;2(2):e0000106. doi: 10.1371/journal.pmen.0000106 (PMC12798219; doi:10.1371/journal.pmen.0000106)
Supplement: S4 Text — This file presents the adapted deliberate self-harm questionnaire for Samoan adolescents, reformatted to align with the original screening instrument to facilitate administration. (PDF) [file pmen.0000106.s005.pdf]

Administering the deliberate self-harm questionnaire for Samoan adolescents:

Samoan adolescents might not answer sensitive topics in questionnaires honestly. To promote honesty, before administering this questionnaire, please make an effort to build trust and create a safe space for the adolescent. Suggestions include:

- It is ideal if the questionnaire is administered by a person the adolescent does not already know (such as a stranger) and also speaks and understands the questionnaire in both English and Samoan to assist the adolescent to answer survey completely and truthfully.
- Administer the questionnaire in a private space, with either just you and the adolescent, or the adolescent alone.
- Before administering the questionnaire, take time to build trust and connection. This could include:
    - Asking the adolescent questions about their interests and actively listening to their answers
    - Employing a non-judgmental and warm demeanor
    - Being present, which includes giving the adolescent your full attention
    - Explaining that any information shared will be held confidential, and clearly communicating which instances under mandatory reporting requirements (if any) would require reporting information to their families
- Clearly communicate the intention behind the questionnaire (such as, to understand how common certain mental health problems are, or to understand what you are going through to help you feel better). Clearly communicate that the goal of asking them these questions is not to get them or anyone they know in trouble.
- Provide the adolescent the opportunity to ask questions before they begin the questionnaire.

E ono lē tali sa'o e tupulaga Samoa ni mataupu ma'ale'ale i pepa fesili. Ina ia u'unaia ona tali mai ma le faamaoni, faamolemole taumafai i se faiga e faatuatuaina ai ma iloa ai e tupulaga e saogalemu a latou faamatalaga ia te oe.
E mafai ona aofia ai fautuaga nei:

- *E pito sili pe afai e faatautaia le pepa fesili e se isi latou te lē iloa (e pei o se tagata ese), ma e tautala ma malamalama i le Pepa Fesili i le Igilisi ma le faa-Samoa, ina ia fesoasoani i le talavou ia atoatoa ma faamaoni a latou tali.*
- *Ia faatautaia le taliga o le pepa fesili i se nofoaga e le o tatalaina i le lautele, e na 'o oulua ma le talavou, pe na o ia fo'i.*
- *A o le'i faatumua le pepa fesili, fai se lua taimi ia tupu ai lona faatuatuaina o oe ma fesoota'i lelei atu. E mafai ona aofia ai:*
    - *Lou fesili i ai i mea latou te fiafia i ai ma matuā faalologo lelei i a latou tali.*
    - *Ia faaalia lou lē faamasino tagata ma ni ou uiga mafanafana.*
    - *Ia iai ma latou, e aofia ai ma le tuu atoa i ai o lou loto i lou taimi ma le talavou*
    - *Faamalamalama i ai o soo se faamatalaga e tuu atu e le faailoā i se isi, ma ia manino lelei ni taimi (pe a iai) e ono lipoti ai ia faamatalaga i lona aiga*
- *Ia faailoa manino le mafuaaga o le pepa fesili (e pei o le fia malamalama poo le a le taatele o nisi o faafitauli tau le maloloina o le mafaufau o alia'e, poo le malamalama i se tulaga o e iai ina ia iloa le auala sili e fesoasoani atu ai ia suia i le lelei ou lagona). Ia manino ona faailoa atu, o le faamoemoe o fesili e lē ina ia aafia ai ia poo se isi latou te iloa.*
- *Tuu se avanoa i le talavou e fai mai ni fesili ae le'i amata ona tali le pepa fesili.*

The following pages were adapted from the original Avon Longitudinal Study of Parents and Children (ALSPAC) questionnaire and the Self-Injurious Thoughts and Behaviors Interview (SITBI). The layout, scoring, and administrative guidelines are taken verbatim from the original instrument; questions and prompts were adapted and translated for Samoan adolescents. For more information on the adaptation process, please see Mew et al., 2024 (peer-reviewed publication in PLOS Mental Health).

DELIBERATE SELF-HARM QUESTIONNAIRE
FOR SAMOAN ADOLESCENTS

Life has many ups and downs. Sometimes people feel upset. These feelings can be so bad that people who have them may feel suicidal or want to self-harm. The following questions ask you about your feelings and the feelings of people close to you. We know this is a sensitive subject, but it is important to ask about it now, as it is not uncommon. By finding out about self-harm we can find ways of helping people.

E tele taimi e lūga lālo ai le olaga. O isi taimi e lagona ai e tagata le lē fiafia. O nei lagona e iai taimi e matuā tugā ai, ma o'o ai i se tulaga e lagona ai e e ua aafia ai, le fia pule i le ola, pe faamanu'alia o ia lava. O fesili o i lalo, o le a fesiligia ai ou faalagona, ma lagona o tagata e latalata ia te oe. Matou te iloa e ma'ale'ale lenei mataupu ae taua pe a tatou fa'asoa i ai,āua e le o se mea fou. I le suesueina o le mataupu i le faamanu'alia o lou lava tagata, e mafai ai ona tatou maua ni auala e fesoasoani ai i ia tagata.

SOCIAL NETWORKS

1. Has anyone in your family (not including yourself) ever hurt themselves on purpose (e.g. by taking an overdose of pills, or by cutting themselves)?
Sa iai se isi o le tou aiga (e le faitauina ai oe) na faamanu'alia e ia ia lava (faataitaiga, so'ona inu fuala'au po o le selei/tatipi o le tino)?

Yes / *Ioe* ☐ 1   No / *Leai* ☐ 2

2. Have any of your close friends ever hurt themselves on purpose?
E iai ni au uo mamae na faamanu'alia ma le mautinoa ia latou lava?

Yes / *Ioe* ☐ 1   No / *Leai* ☐ 2

3. a. Has anyone you have ever known died by suicide?
E iai se isi e te iloa na oti ona o le pule I le ola?

Yes / *Ioe* ☐ 1   No / *Leai* ☐ 2

b. If so, how many? *Afai e iai, e to'afia?* ________

SUICIDAL THOUGHTS

4. How old were you the first time you had thoughts of killing yourself?
E fia ou tausaga i le uluai taimi na e mafaufau ai e te pule i lou ola?

____

5. How old were you the last time?
E fia ou tausaga i le taimi mulimuli na tupu ai?

____

SELF-HARMING BEHAVIORS

6. Have you ever hurt yourself on purpose in any way (e.g. by taking an overdose of pills, or by cutting yourself)?
Na iai se taimi na e faamanu'alia ai ma le mautinoa oe lava i so'o se auala (pei o le so'ona inu o fualaau po'o le tatipi o lou tino)?

Yes / *Ioe* ☐ 1 No / *Leai* ☐ 2

If yes:
Afai e ioe:

7. How many times have you done this in the last year? Please mark one box only.
E faafia ona e faia lea tulaga i le tausaga ua tuana'i? Faamolemole, maka na'o le pusa e tasi.

Once / *Faatasi* ☐ 1 2-5 times ☐ 2

6-10 times ☐ 3 More than 10 times / *Sili atu ma le 10* ☐ 4

8. When was the last time you hurt yourself on purpose? Please mark one box only.
O le a le taimi mulimuli na e faamanu'alia ai ma le mautinoa oe lava? Faamolemole, maka na'o le pusa e tasi.

In the last week / *Vaiaso ua te'a* ☐ 1 More than a week ago but in the last year / *Ova atu ma le vaiaso ua tuana'i* ☐ 2 More than a year ago / *Sili atu ma le tausaga* ☐ 3

9. How old were you the last time you hurt yourself on purpose?
E fia ou tausaga i le taimi mulimuli na e fuafuaina ai e faamanu'alia oe lava?

____

10. The last time you hurt yourself on purpose, which of the actions below best describes what you did? Please mark all boxes that apply.
I le taimi mulimuli na e faamanu'alia ai ma le mautinoa oe lava, o a gaoioiga o loo lisi atu i lalo e faamatalā lelei le mea sa e faia? Faamolemole maka uma pusa e fetaui.

i) Swallowed pills or something poisonous *| Inu ni fualaau poo se mea oona* ☐ 1

ii) Cut or carved skin *| Tatipi le pa'u o le tino* ☐ 1

iii) Burned your skin (i.e., with a cigarette, match or other hot object) *| Susunu lou tino (i se sikaleti, afitusi, poo se isi mea vevela)* ☐ 1

iv) Inserted sharp objects into your skin or nails *| Sulu ni mea maai i le pa'u o lou tino poo atigilima* ☐ 1

v) Picked areas of your body to the point of drawing blood *| Iini vaega o lou tino se'ia maligi le toto* ☐ 1

vi) Hit yourself on purpose *| Ta oe lava ma le mautinoa* ☐ 1

vii) Scraped your skin to the point of drawing blood *| Vavalu ese le pa'u o le tino se'ia maligi le toto* ☐ 1

viii) Something else *| Se isi mea* ☐ 1

Please say what:
| Faamolemole faailoa mai poo le a:
……………………………………………………………

11. Do any of the following reasons help to explain why you hurt yourself on that occasion? Please mark all boxes that apply.
E iai se mafuaaga o ta'ua i lalo e fesoasoani e faamatala le mafuaaga na e faamanu'alia ai oe lava i lena taimi? Faamolemole maka le pusa e talafeagai.

i) I wanted to show how desperate I was feeling *Na ou mana'o e faaali o'u lagona fia maua se lavea'i* ☐ 1

ii) I wanted to die */ Na ou fia oti* ☐ 1

iii) I wanted to punish myself */ Na ou mana'o e fa'asala a'u* ☐ 1

iv) I wanted to frighten someone */ Na ou mana'o e faafefe se isi* ☐ 1

v) I wanted to get relief from a terrible state of mind */ Na ou mana'o ou te mapu mai mafaufauga le lelei* ☐ 1

vi) Some other reason */ Nisi mafuaaga* ☐ 1

Please say what:
/ Faamolemole faailoa mai:

……………………………………………………………

12. After you had hurt yourself on that occasion, how did you feel? Please mark **one** box only.
Ina ua mae'a ona faamanu'alia oe lava i lena taimi, o le a sou lagona na i ai? Faamolemole maka le pusa e tasi.

Better than before */ Sili atu nai lo le taimi muamua* ☐ 1	The same as before */ Tutusa ma le taimi muamua* ☐ 2	Worse than before */ Leaga atu i lo le taimi muamua* ☐ 3

SUICIDE PLANS AND ATTEMPTS

13. Have you ever made plans to kill yourself (for example, a specific plan towards where, when, and how)?
Na iai se taimi na e fuafua ai e te pule i lou ola (mo se faataitaiga, o le a sau fuafuaga na tapena i le nofoaga, le taimi ma le aso, ma e faapefea fo'i ona faataunuu)?

Yes / *Ioe* ☐ 1   No / *Leai* ☐ 2

14. How many times did you try to kill yourself or make a suicide attempt?
E faafia ona e taumafai e fasioti oe, pe taumafai foi e pule i lou ola?

15. What age(s) were you when you made your suicide attempt(s)?
O le fia o ou tausaga na e taumafai ai e te pule i lou ola?

HELP-SEEKING BEHAVIORS

16. The last time you hurt yourself in any way (e.g. by taking an overdose of pills, or by cutting yourself) did you seek medical help/first aid from any of the following? Please mark all boxes that apply.
I le taimi mulimuli na e faamanu'alia ai oe lava i soo se auala (faataitaiga soona inu fualaau, poo le tatipi o lou tino, na e saili mo se fesoasoani i le falema'i, poo se isi lava pei ona lisi atu i lalo? Faamolemole maka pusa e talafeagai.

i) Crisis phone line / *Telefoni/laina fesoasoani* ☐ 1

ii) Reaching out to a friend / *Fesoota'i i se uo* ☐ 1

iii) Reaching out to a trusted adult / *Fesootai i se tagata matua faatuatuaina* ☐ 1

iv) GP (family doctor) / *Foma'i (a le aiga)* ☐ 1

v) Mental health professional / *Tagata tomai i le maloloina o le mafaufau* ☐ 1

vi) Hospital causality/emergency department / *Matagaluega o ma'i faafuase'i i le Falema'i* ☐ 1

vii) Other / *Isi* ☐ 1

Please say who this was:
Faamolemole fa'ailoa mai poo ai lea:

……………………………………………..

17. Have you ever tried to get help from someone or somewhere about hurting yourself on purpose, or about wanting to kill yourself?
Na iai se taimi na e taumafai e saili se fesoasoani mai se isi po o se nofoaga e uiga i lou faamanu'alia o oe lava, poo le fia pule fo'i i le ola?

Yes / *Ioe* ☐ 1 No / *Leai* ☐ 2

If yes:
Afai e ioe:

18. Who have you been to for help? Please mark all boxes that apply.
O ai na e o'o i ai mo se fesoasoani? Faamolemole maka uma pusa e talafeagai.

i)	Mother / *Tina*	☐ 1
ii)	Father / *Tama*	☐ 1
iii)	Brother / *Tuagane*	☐ 1
iv)	Sister / *Tuafafine*	☐ 1
v)	Someone else in your family / *Se isi o lou aiga*	☐ 1
vi)	A friend / *Uo*	☐ 1
vii)	A teacher / *Faia'oga*	☐ 1
viii)	High school counsellor / *Faufautua Aoga/Maualuga*	☐ 1
ix)	Peer mentor / *Maualuga*	☐ 1
x)	A GP (family doctor) / *Foma'i a le aiga*	☐ 1

xi) A social worker
/ Tagata i le galuega tauagafesootai 1

xii) A psychologist or psychiatrist
/ Tagata tomai tau saikolo ma le foma'i o le mafaufau 1

xiii) A telephone crisis line
/ Laina telefoni Fesoasoani 1

xiv) Drug dealer
/ Tagata faatau fualaau faasaina 1

xv) Social media
/ Ala o faasalalauga a tagata faitele 1

xvi) Sports coach
/ Faiaoga tau taaloga 1

xvii) Somewhere else (e.g. internet, book, magazine, other person, etc.)
/ Se isi fesoasoani (faataitaiga Initaneti, tusi, mekasini, isi) 1

xviii) Other trusted adult
/ Se isi tagata matua Faatuatuaina 1

Please say what or who:
/ Faamolemole faailoa mai poo le a poo ai:

…………………………………………………..
